# Non-contact friction in ultracoherent nanomechanical resonators near dielectric materials

**DOI:** 10.1038/s41567-026-03350-z

**Published:** 2026-07-07

**Authors:** Amirali Arabmoheghi, Alessio Zicoschi, Guillermo Arregui, Mohammad J. Bereyhi, Yi Xia, Nils J. Engelsen, Tobias J. Kippenberg

**Affiliations:** 1 Swiss Federal Institute of Technology Lausanne (EPFL), Lausanne, Switzerland; 2 Department of Microtechnology and Nanoscience (MC2), Chalmers University of Technology, Göteborg, Sweden

**Keywords:** Optomechanics, Sensors

## Abstract

Micro- and nanomechanical resonators are emerging as promising platforms for quantum technologies, precision sensors and fundamental science experiments. To utilize these devices for force sensing or quantum optomechanics, they must be brought in close proximity with other systems for functionalization or efficient readout. Improved understanding of the loss mechanisms in nanomechanical resonators, specifically the advent of dissipation dilution, has led to the development of ultracohorent devices with mechanical quality factors exceeding one billion at room temperature, setting their force sensitivities surpassing those of the state-of-the-art atomic force microscopes. Given this new regime of sensitivity, an intriguing question is whether the proximity of other materials hinders mechanical coherence. Here we report a dissipation mechanism that occurs in ultracoherent nanomechanical oscillators caused by the presence of nearby dielectrics. By studying the parameter scaling of the effect, we show that the mechanism is more severe for low-frequency mechanical modes. This is due to dielectric loss within the materials caused by the motion of a resonator carrying static charges. These observations are consistent with the non-contact friction observed in atomic force microscopes. Our findings provide insights into limitations on the integration of ultracoherent nanomechanical resonators and highlight the adverse effects of charged defects in these systems.

## Abstract (web summary)

Understanding loss mechanisms in nanomechanical resonators has enabled ultracoherent devices with improved sensitivity. Now these resonators are found to be sensitive to non-contact friction from nearby materials, thus limiting mechanical coherence.

## Main

Micro- and nanomechanical oscillators have long been used in science and technology alike, as they can couple to a wide range of other degrees of freedom. However, their miniaturization renders them more susceptible to dissipative mechanisms. Reaching quality factors beyond 10^9^, comparable to those in bulk systems^1^, was not achieved in micro- and nanomechanical resonators until recently^2–7^. A large family of ultrahigh-*Q* resonators is based on flexural modes of tensioned and high-aspect-ratio structures (that is, membranes and strings)^8^. In these resonators, dissipation dilution, a phenomenon first introduced in the gravitational wave detector community^9^, enhances the quality factor of the modes by many orders of magnitude beyond the material’s intrinsic quality factor^10^. String-like resonators with soft-clamped modes^4–6,11,12^ are particularly promising force sensors as they exhibit both low mass (*m*, in the sub-nanogram range) and low damping rate (*Γ*_*m*_ < 1 mHz). The thermal-limited force sensitivity of mechanical oscillators at temperature *T* is commonly defined as the single-sided spectrum of the thermal force, that is, $\sqrt{{S}_{{\rm{F}}}^{\mathrm{th}}}=\sqrt{4{k}_{{\rm{B}}}Tm{\varGamma }_{m}}$, where *k*_B_ is the Boltzmann constant. For these ultracoherent devices, it reaches values below $1\,\mathrm{aN}/\sqrt{\mathrm{Hz}}$ at room temperature^4^, surpassing the sensitivity level of the state-of-the-art atomic force microscope (AFM) cantilevers^13^.

**Fig. 1:**
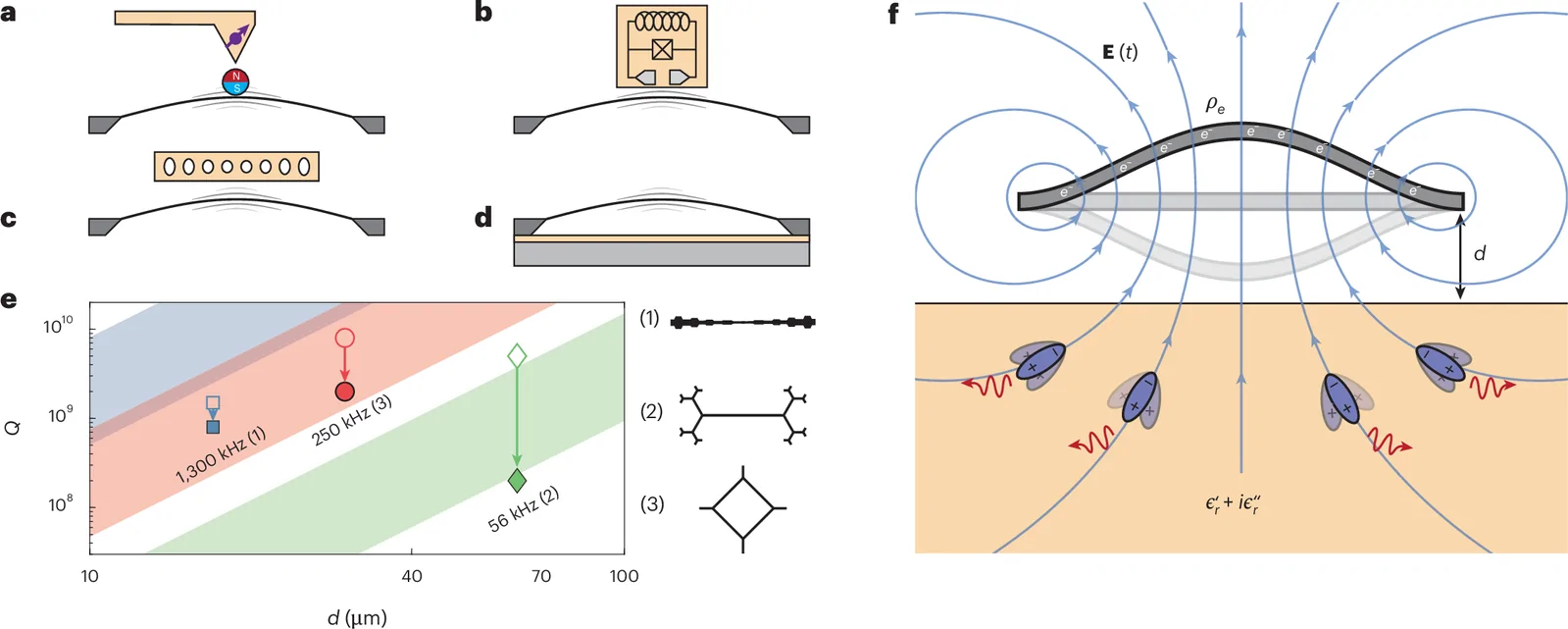
NCF in nanomechanical resonators near dielectrics. **a–c**, Schematics of platforms for coupling nanomechanical resonators to spins in solids^18^ (**a**), superconducting circuits^20^ (**b**) and nanophotonics cavities^23^ (**c**). In these platforms there are dielectric materials close to the resonator. **d**, Even in the absence of any external objects, the dielectrics on the substrate can also introduce loss. **e**, Dielectric loss within the substrate limiting the quality factor of ultracoherent strings as a function of the string-substrate distance *d* (shown in **f**). The colours blue, red and green correspond to (1) strained-engineered^11^, (2) hierarchical^12^ and (3) polygon^4^ designs, shown in the right hand side. These modes have effective masses of 5, 46 and 24 pg, respectively. The ribbons correspond to the estimated NCF-limited quality factor for the frequency and geometry of each design, suspended above a silicon substrate with a 4-nm native SiO_2_ layer. The filled and empty markers correspond to the measured and simulated quality factors adapted from the references. **f**, Concept of dielectric-induced mechanical loss. The charged nanomechanical resonator makes an electric field, which polarizes the dielectrics nearby. This field couples the motion of the resonator to the lossy polarization of the dielectric, which results in dissipation of the mechanical energy within the dielectic. Source data

Virtually all applications that require low mechanical dissipation rely on short-range interactions and require placing other objects at sub-micron distances from the nanomechanical resonator. For example, measurements of Casimir forces^14^, tests of gravity at short distances^15^ and precision scanning force microscopy experiments^16,17^ all occur at short distances. Moreover, as illustrated in Fig. 1a–c, several platforms for coupling nanomechanical resonators to other degrees of freedom, such as spin qubits^18,19^, superconducting circuits^20^ and optical microcavities^21–25^ also rely on near-field interactions. The latter is of particular interest as the optomechanical coupling, which enables readout of the motion of the resonator with quantum-limited precision^26^, scales exponentially with the distance between optical and mechanical resonators. So far, the devices used in these applications^16–19,21–25^ do not exhibit the force sensitivities, ${S}_{{\rm{F}}}^{\mathrm{th}}$, as low as those of the stand-alone ultrahigh-*Q* devices discussed previously, which are suspended tens of micrometres away from the closest object. This naturally raises the question whether ultracoherent mechanical devices, with force sensitivity below that of AFMs, can retain their coherence properties near other materials.

In this work, we show that nearby dielectrics do perturb nanomechanical resonators. We report on a loss mechanism occurring when ultracoherent string resonators are integrated in the near-field of a photonic crystal (PhC) cavity. We observe that the quality factor of the high-*Q* modes of a binary-tree-shaped resonator^12^ is reduced for smaller cavity-resonator separations. Moreover, for millimetre-long strings, even the presence of the substrate supporting the structures can cause additional loss in the low-frequency modes. By identifying that the added loss is inversely proportional to the mechanical mode’s frequency, we realize that it is due to an electrical interaction between the resonator and the dielectrics in its proximity (that is, the optical cavity or the substrate) mediated by static charges. This phenomenon, also known as non-contact friction (NCF), has only been observed in AFM cantilevers^27–30^, where a functionalized tip is brought in close distance from a sample to measure surface properties. In nanomechanical systems, outside the context of AFMs, these effects have been largely overlooked due to larger inter-body distances and the fact that most resonators are not intentionally charged. However, the presence of trapped charges on microfabricated devices, in particular with amorphous materials, has been commonly reported^31–35^. We have developed a theoretical and numerical framework for studying NCF in nanomechanical systems in the presence of static charges and we show that they explain our observations very well. Intriguingly, isolated ultracoherent devices have long shown a persistent discrepancy between measured and predicted *Q* for their low-frequency modes^4,11,12^ (Fig. 1d,e). We show that this discrepancy is most likely due to NCF from the substrate, located tens of microns away.

## Dielectric-induced mechanical loss

NCF has been modelled in AFM systems as the interaction of a moving point charge with a lossy dielectric^28,30^. This model can be readily generalized to nanomechanical resonators with distributed electric charges. As shown in Fig. 1f, a nanomechanical resonator (for example, a doubly clamped string), with a volume charge density ${\rho }_{e}({\bf{r}})$, is placed near a rigid dielectric body with the complex frequency-dependent permittivity ${\epsilon }_{r}({\bf{r}},\omega )={\epsilon }_{r}^{{\prime} }({\bf{r}},\omega )+i{\epsilon }_{r}^{{\prime\prime} }({\bf{r}},\omega )$. ${\epsilon }_{r}^{{\prime\prime} }({\bf{r}},\omega )$ characterizes the material’s losses (which generally include both dielectric and conductive components). In the equilibrium position, the electric field in the space is ${{\bf{E}}}_{0}({\bf{r}})$. Consider a vibrational eigenmode of the resonator with frequency *Ω*_*m*_ and mode shape ${{\bf{U}}}_{m}({\bf{r}})$. Displacements in this mode, $u(t)=x{{\rm{cos}}}({{\Omega }_{m}}{t})$ with the amplitude *x*, modify the electric field as

1 $${\bf{E}}({\bf{r}},t)={{\bf{E}}}_{0}({\bf{r}})+{\bf{g}}({\bf{r}},{\varOmega }_{m};{{\bf{U}}}_{m}({\bf{r}}))u(t),$$

where ${\bf{g}}({\bf{r}},{\varOmega }_{m};{{\bf{U}}}_{m}({\bf{r}}))=\frac{{\rm{\delta }}{{\bf{E}}}_{0}({\bf{r}})}{{\rm{\delta }}u}$ is the electric field variations under the displacements of the mode ${{\bf{U}}}_{m}$ (see Supplementary Information for theoretical details). Due to the imaginary part of *ϵ*_*r*_, the motion of the resonator results in dissipation within the dielectric with the period-averaged dissipated power density ${\bar{p}}_{d}({\bf{r}})=\frac{{\varOmega }_{m}}{2}{\epsilon }_{0}{\epsilon }_{r}^{{\prime\prime} }({\bf{r}},{\varOmega }_{m})| {\bf{g}}{| }^{2}| x{| }^{2}$. Integrating over all dielectrics gives the period-averaged total power dissipated by the oscillator. Referring the loss to a viscous damping force of the form $-{\gamma }_{{\rm{NCF}}^{{\dot{u}}(t)}}$, the dissipated power would be ${\bar{P}}_{d}=\frac{{\varOmega }_{{m}}^{{2}}}{2}{\gamma }_{\mathrm{NCF}}| x{| }^{2}$. Comparing these expressions yields the NCF damping coefficient

2 $${\gamma }_{\mathrm{NCF}}=\int \,{d}^{\,3}r\,{\epsilon }_{0}| {\bf{g}}({\bf{r}},{\varOmega }_{m};{{\bf{U}}}_{m}({\bf{r}})){| }^{2}\frac{{\epsilon }_{r}^{{\prime\prime} }({\bf{r}},{\varOmega }_{m})}{{\varOmega }_{m}}.$$

For insulators with frequency-independent losses, the inverse-frequency scaling (*γ*_NCF_ ∝ 1/*Ω*_*m*_) provides a clear experimental signature of NCF in nanomechanics.

**Extended Data Fig. 1:**
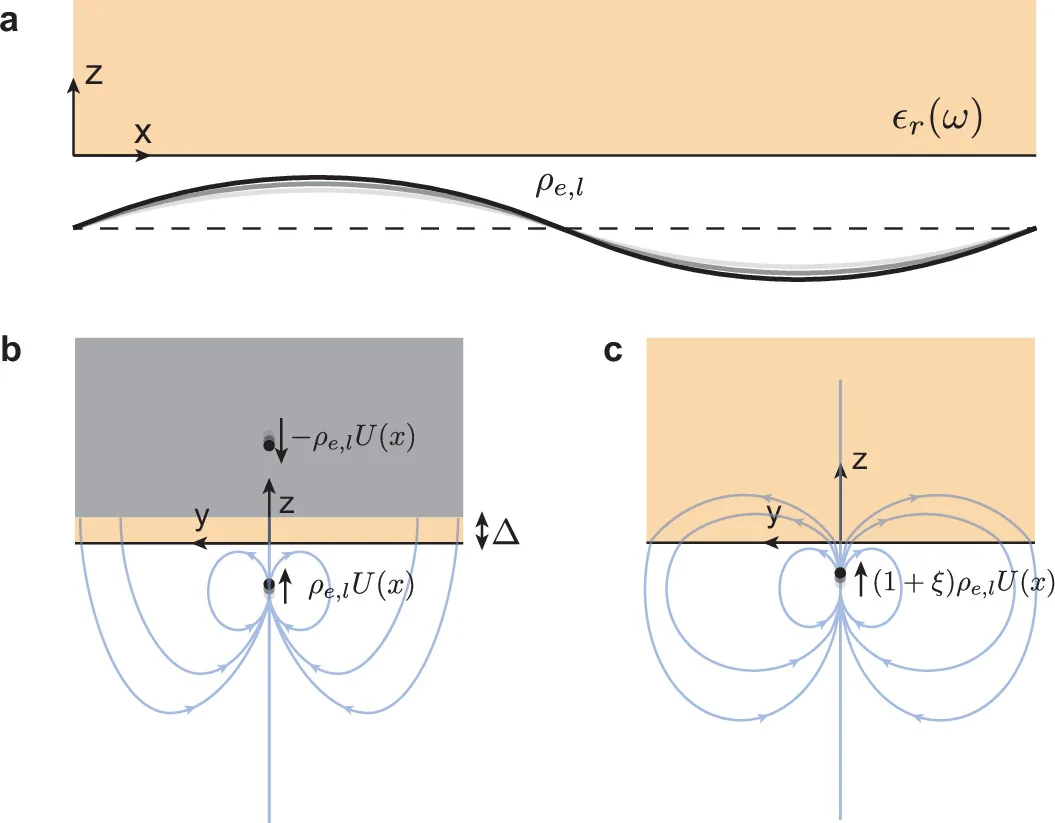
Analytical modeling of NCF for an infinitesimally thin string above a substrate. **a** The string is along the x-axis and it oscillates along the z-axis. It is located in air, with distance *d* from the substrate **b** View of **a** in the yz plane, for the situation where the substrate is conductive with a thin dielectric layer. We show the motion of the string and the electric field variation (blue streamlines). **c** Same as **b**, but for a semi-infinite dielectric substrate with complex relative permittivity *ϵ*_*r*_(*ω*).

To test whether the *Q* discrepancy in isolated ultracoherent devices (Fig. 1d,e) can be attributed to NCF, we apply equation (2) to ultracoherent Si_3_N_4_ strings suspended above silicon substrates, estimating the NCF-limited *Q* from dielectric loss in the native oxide layer (Methods and Extended Data Fig. 1). As shown in Fig. 1e, assuming surface charge densities in the 1.45 × 10^11^ to 5.8 × 10^11^ e cm^−^^2^ range^33^, the model yields estimates with large variations, yet they are sufficient to account for the measured *Q* reduction across three previously reported devices—with elastic strain engineering^11^, hierarchical^12^ and polygon^4^ designs.

## NCF in strings suspended above a dielectric substrate

**Fig. 2:**
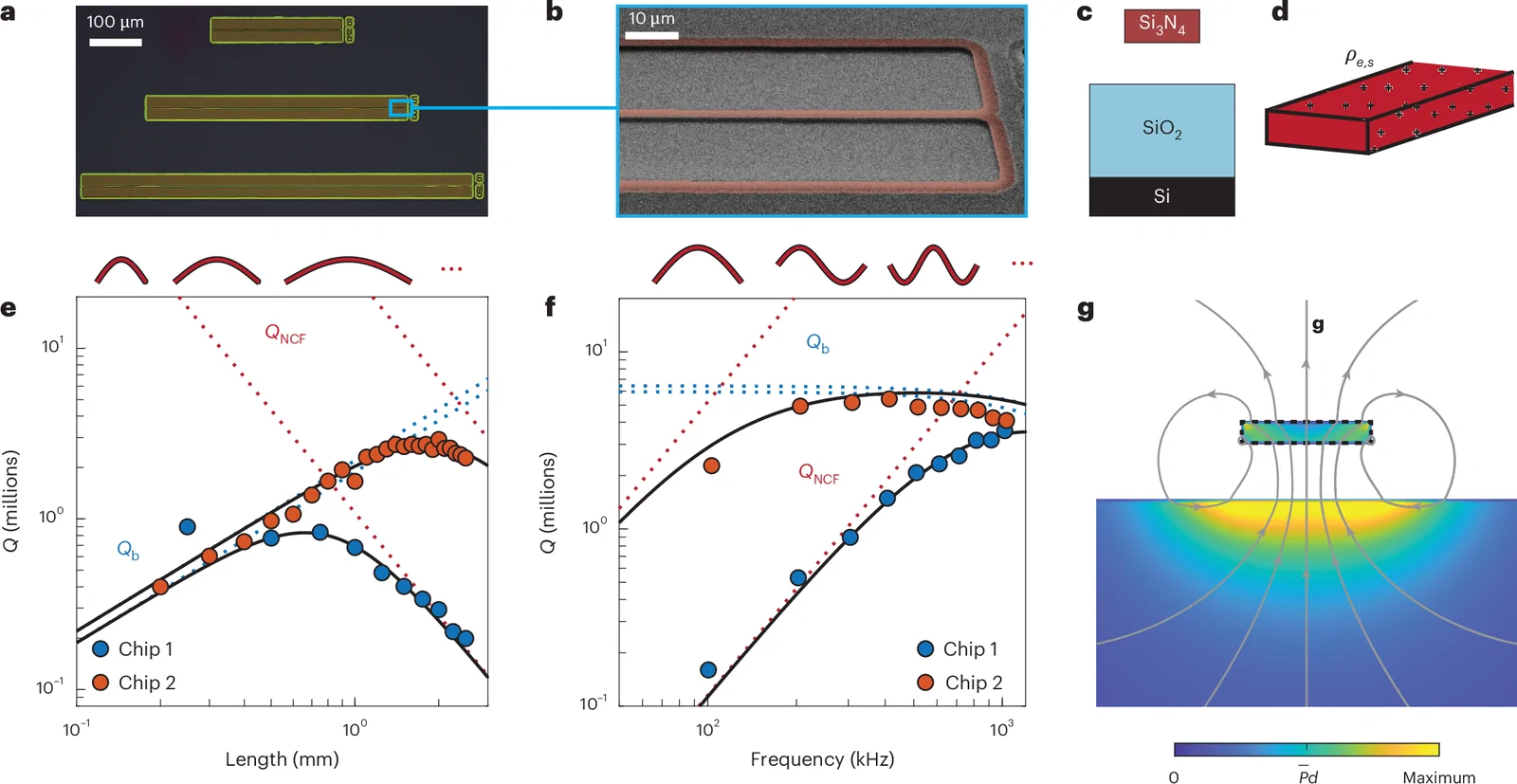
NCF in strings suspended above a dielectric surface. **a**, Optical microscope image of a few uniform doubly clamped beams with lengths 250, 500 and 750 μm, suspended above the SiO_2_ layer. **b**, False-coloured scanning electron microscope micrograph of a suspended nano-beam taken close to its clamping point (on the right hand side) where red corresponds to suspended Si_3_N_4_. **c**, Full stack of the structures after the release. Si_3_N_4_ is suspended over a thermally oxidized Si substrate. **d**, Rectangular cross-section of the strings. For FEM modeling, we assume a uniform surface charge density *ρ*_*e*,*s*_ on all the surfaces. **e**, Measured *Q* for the fundamental modes of nano-beams with different lengths. Blue and orange circles correspond to experimental data from chips 1 and 2, respectively. The solid black lines show fits from the *Q*_tot,*n*_ model. The dotted blue and red lines correspond to the bending and NCF components of the fits, respectively. **f**, Measured *Q* for the higher-order modes of the 2.5-mm-long beams. The markers, lines and colours denote the same as in **e**. **g**, FEM simulation of NCF for the stack shown in **c**. The dashed lines on the edges of the string indicate the charged surfaces. The string is 1.5 μm wide and 227 nm thick. The gap is 680 nm. The grey lines correspond to the streamlines of the **g** field. The colour map shows the dissipated power density. Source data

**Extended Data Fig. 2:**
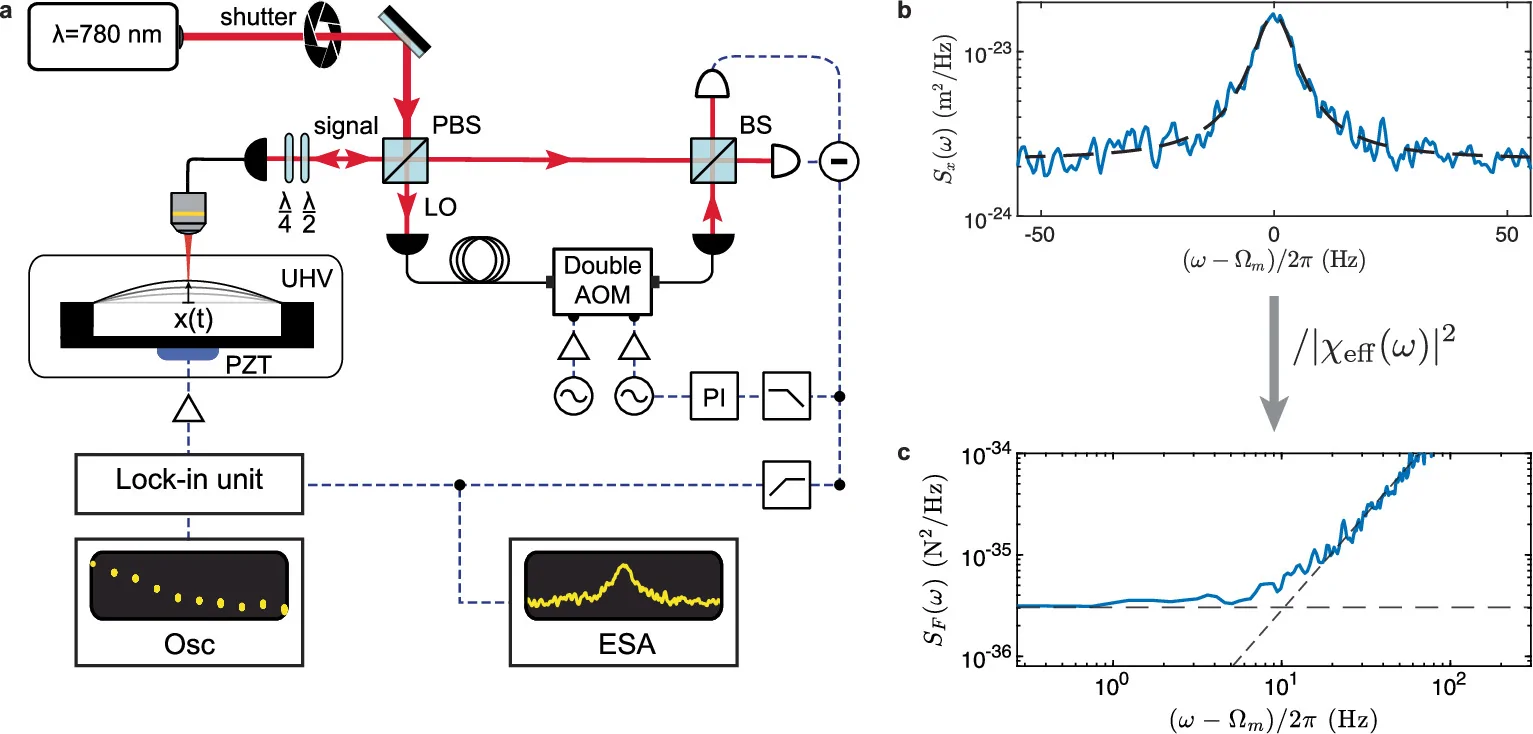
Experimental setup and measurement techniques. **a** Schematic of the measurement setup. Red lines correspond to free space optical paths, black lines correspond to optical fibers and blue dashed lines correspond to electrical connections. The sample is placed in an ultra-high vacuum chamber (HUV) with optical access. A piezoelectric actuator (PZT) is installed for exciting the mechanical modes. Linearly polarized light from a 780 nm diode laser is split using a polarizing beam splitter (PBS) into the signal and local oscillator (LO) paths. On the signal path light is focused on the sample using a microscope objective and the reflected light, which is separated from the incoming light using quarter and half waveplates (*λ*/4 and *λ*/2), is guided towards the homodyne interferometer. The LO path contains a fiber spool, for length balancing the paths, and the double acousto-optic modulator (AOM), which contains two AOMs in series, one shifting the optical frequency up by 100 MHz and the other shifting it down by the same ammount. The LO and signal are compined on a beam splitter (BS) and detected using a balanced photodetector. The detector signal is separated into low and high frequency components using low-pass and high-pass filters. One of the AOMs is driven by an electrical oscillator with a fixed frequency and the other is driven by a voltage-controlled oscillator whose frequency is modulated by the low frequency component of the homodyne signal through a feedback controller (PI). This mechanism enables stabilizing the optical path length difference and homodyne phase. The high frequency component is directly detected using an electrical spectrum analyzer (ESA) and it is directed to a lock-in amplifier unit for ringdown measurements. **b** Measured thermomechanical spectrum of the fundamental mode of the nano-optomechanical device shown in Fig. fig:NCF_in_OMd under feedback cooling. The blue line is the experimental data and the dashed black line is a Lorentzian fit to the data. **c** Measured thermal force spectrum obtained from division of the the spectrum in **b** by the susceptibility of the feedback cooled mode. The blue line is the experimental data and the dashed black lines correspond to the thermal force noise floor and the imprecision noise branch.

For the simple system shown in Fig. 1f, we can find an analytical expression for the NCF damping coefficient. The string is infinitesimally thin and carries linear charge density *ρ*_*e*,l_. For this system, we can find the electric field variation using the elementary method of image charges (see Methods and Extended Data Fig. 1 for details). The NCF-limited quality factor for mode order *n* with frequency *Ω*_*n*_ is given by

3 $${Q}_{\mathrm{NCF},n}=\frac{8{\uppi }{\epsilon }_{0}{\rho }_{m,{\rm{l}}}{d}^{\,2}}{{\rho }_{e,{\rm{l}}}^{2}}\frac{{\varOmega }_{n}^{2}}{\mathrm{Im}[\xi ({\varOmega }_{n})]},$$

where $\xi (\omega )=\frac{{\epsilon }_{r}(\omega )-1}{{\epsilon }_{r}(\omega )+1}$ and *ρ*_*m*,l_ is the linear mass density. To experimentally study this model, we fabricated uniform high-stress (~1 GPa) Si_3_N_4_ strings with a 200-nm-thick rectangular cross-section, suspended *d* = 500 nm above a SiO_2_-on-silicon substrate (Fig. 2a–d), with lengths ranging from 200 to 2,500 μm (see Supplementary Information for fabrication details). We measured the quality factor of their out-of-plane (OP) modes under vacuum (*P* < 10^−8^ mbar) via ringdown measurements using an optical interferometer (Methods and Extended Data Fig. 2). For these modes, when no dielectrics are nearby, the quality factor is limited by bending loss. For a string of thickness *h* and length *L*, it is given by the dissipation dilution theory^10^

4 $${Q}_{{\rm{b}},n}=\frac{{Q}_{\mathrm{int}}}{2\lambda +{n}^{2}{\uppi }^{2}{\lambda }^{2}},$$

where *Q*_int_ is the intrinsic mechanical *Q* of the material, and $\lambda =\sqrt{\frac{E}{12\sigma }}\frac{h}{L}$, with *E* and *σ* being the Young’s modulus and stress, respectively. For strings with high aspect ratio and high stress, *λ* ≪ 1, and the quality factor of the fundamental mode is approximately *Q*_*b*,1_ ≈ *Q*_int_/2*λ*, which has a linear dependence on the string length, *L*. We measure the *Q* of the fundamental modes of strings with varying lengths on two different chips from separate fabrication runs. As shown in Fig. 2e, the quality factors markedly deviate below the linear scaling predicted by the bending loss model of equation (4). We also measure the *Q* of the first ten OP modes of the 2,500-μm-long beams from both chips. According to equation (4), because *λ* ≪ 1, we expect a weak descending dependence on the mode frequency. However, the data shown in Fig. 2f indicate that modes with lower frequencies experience substantially more damping.

**Extended Data Fig. 3:**
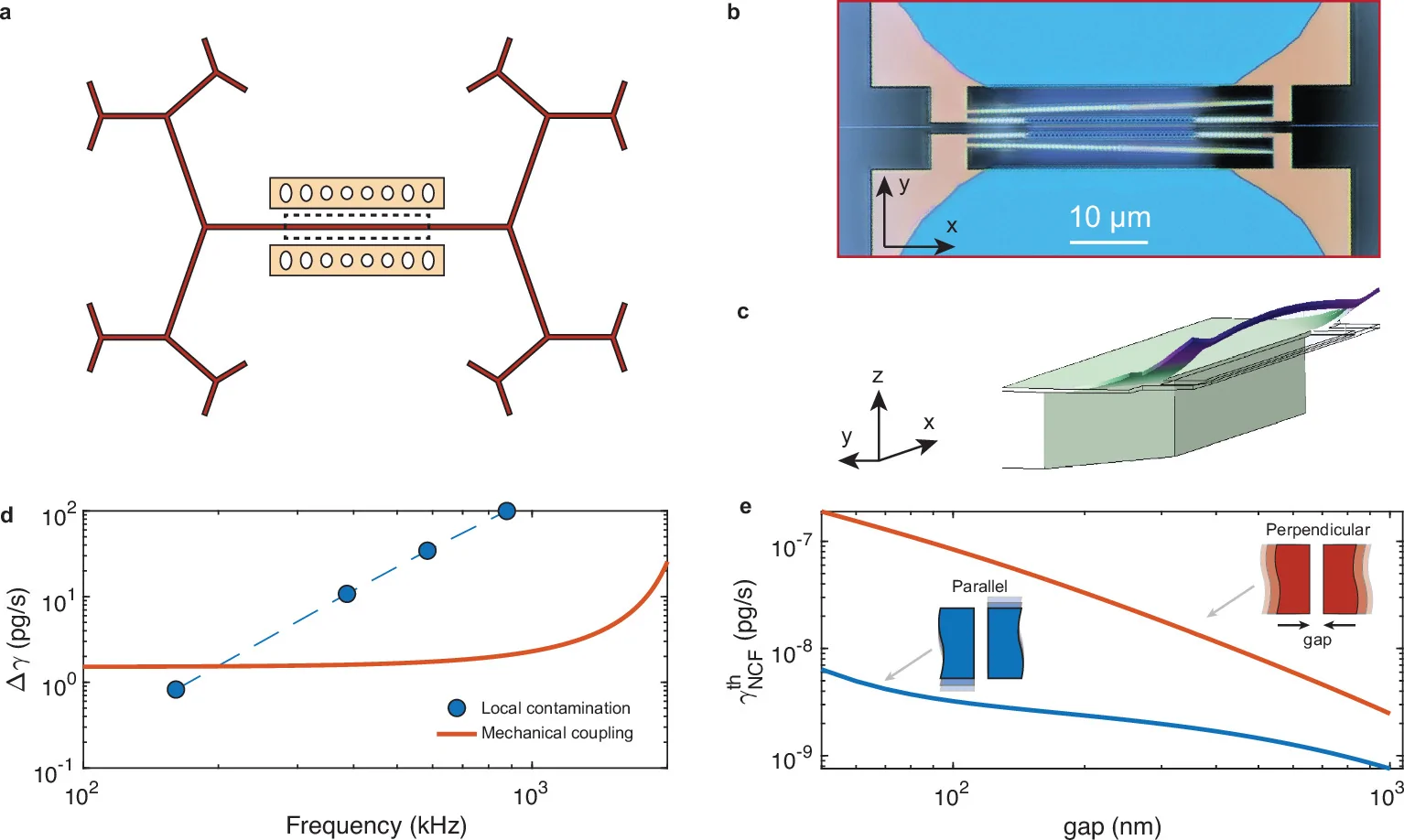
Ruling out other possible damping mechanisms. **a** Schematic of the nano-optomechanical device. The area marked by the dashed box is prone to local reduction of the intrinsic Q. **b** Optical microscope image of coupling region in the nano-optomechanical device. The horizontal thin line in the center is the string and the symmetric structures on the sides are the photonic crystal cavities and their supports. The orange regions are fully undercut Si_3_N_4_ thin films and the blue regions are where the Si_3_N_4_ is still supported by the Si substrate. **c** FEM simulation of the fundamental mechanical mode of the PhC cavity at 2.1 MHz. **d** Additional damping coefficients caused by local contamination (blue circles) and mechanical coupling to the PhC cavity (orange line) versus frequency. **e** Estimation of the fundamental thermal-electrodynamic damping coefficient for two silicon nitride semi-infinite bodies in parallel (blue) and perpendicular (orange) relative motion versus their distance.

The observations are well explained by combining bending loss and NCF: ${Q}_{\mathrm{tot},n}^{-1}={Q}_{{\rm{b}},n}^{-1}+{Q}_{\mathrm{NCF},n}^{-1}$. We fit the data in Fig. 2e,f using *Q*_int_ and ${\rho }_{e,{\rm{l}}}^{2}\mathrm{Im}[\xi ]$ as free parameters, defining an effective linear charge density ${\rho }_{e,{\rm{l}}}^{\mathrm{eff}}$ for the rectangular cross-section (Supplementary Information). Using literature values for SiO_2_ permittivity and loss tangent, we extract ${\rho }_{e,{\rm{l}}}^{\mathrm{eff}}=180\,{\rm{e}}\,{\upmu{\mathrm{m}}}^{-1}\;{{\rm{and}}}\;40\,{\rm{e}}\,{\upmu{\mathrm{m}}}^{-1}$ for chips 1 and 2 from the fundamental modes, consistent within 15% with values from higher-order modes (210 e μm^−1^ and 42 e μm^−1^). The correction due to the conductive silicon substrate is less than 10% for oxide thicknesses >1 μm (Supplementary Information). Notably, while bending contributions are consistent across chips, NCF contributions differ drastically, indicating that charge density is highly fabrication-run dependent. The ${\varOmega }_{m}^{2}$ scaling in *Q* is strong evidence for NCF as the additional loss channel, with no other known mechanism accounting for this behaviour (Methods and Extended Data Fig. 3).

To go beyond the infinitesimally thin string approximation, we developed an finite element method (FEM) model to compute **g** and the NCF coefficient for arbitrary geometries (Supplementary Information). As shown in Fig. 2g, as the mode shape varies slowly compared with the gap size, the simulation is performed on the two-dimensional cross-section of the geometry and the result is extended analytically to the full geometry. For simplicity, as shown in Fig. 2d, we assume a uniform charge distribution on all surfaces. Converting the effective linear charge densities to surface values using our simulation results yields 8.3 × 10^9^ and 1.2 × 10^9^ e cm^−^^2^ for chips 1 and 2, respectively—below the typically reported range of 10^11^ to 10^13^ e cm^−^^2^ (refs. ^33–35^), though reported values vary widely with chemical treatment^35,36^. Additional data suggest a higher charge density on the vertical sidewalls, hinting at plasma etching as a contributing factor (Supplementary Information).

## Observation of NCF in a nano-optomechanical system

**Fig. 3:**
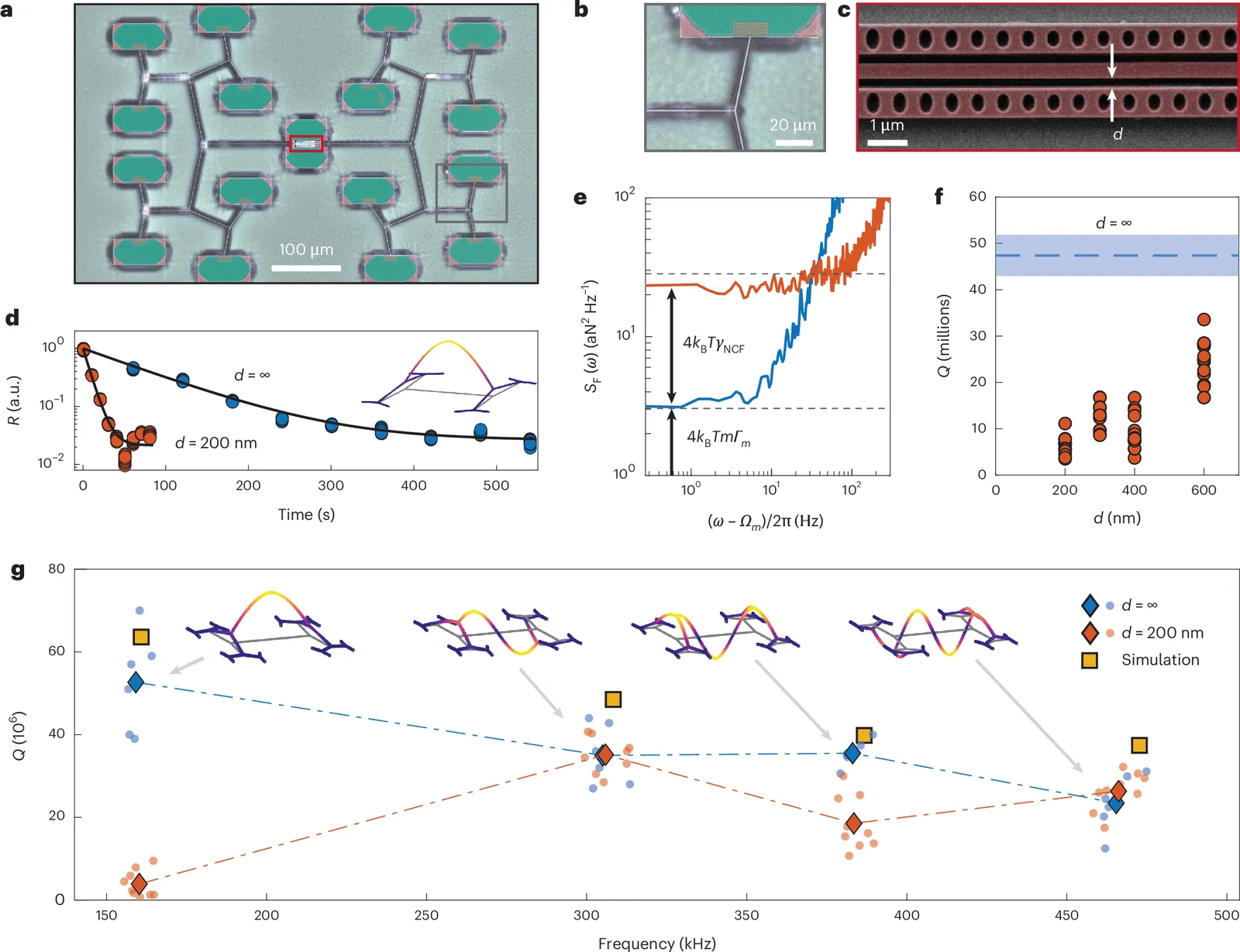
NCF in a nano-optomechanical system. **a**, Optical microscope image of the nano-optomechanical system. **b**, A zoom-out of the section marked with the grey rectangle shows the resonator segments and the clamping points. The PhC cavity is located at the centre of the device, marked with the red rectangle. **c**, False-coloured scanning electron microscope micrograph of the section marked by the red rectangle in **a**, taken before the release, showing the PhC cavities and the string in between them. *d* is the string PhC distance. **d**, Gated ringdown measurements of the mode with the mode shape shown in the inset for a reference device (blue) and an integrated device with gap of 200 nm (orange). *R* is the measured amplitude of motion, normalized to its maximum value. **e**, Measured force noise power spectral density for the reference (orange) and integrated (blue) devices in **d**. The dashed lines correspond to the theoretical value. **f**, A compilation of the measured *Q*s for integrated devices with varying gaps (orange circles) as well as the average of the reference devices (dashed blue line). The blue shade corresponds to the standard deviation of the reference *Q*s. Each circle corresponds to the measurement from one device. **g**, Measured *Q* for the first four OP modes with the mode shapes shown above, for reference (blue markers) and integrated (orange markers) devices with the gap of 200 nm. The circles show single devices and diamonds the average value, and the yellow squares correspond to FEM simulation values. The dash-dotted lines are guides to the eye. Source data

We have implemented on-chip integration of a Si_3_N_4_ PhC microcavity with a binary-tree string resonator^12^ (see Supplementary Information for the fabrication process). A representative device and details of its different elements are shown in Fig. 3a–c. The string is suspended in the gap between two identical PhC cavities; the coupling of the string’s motion to the optical mode enables efficient on-chip displacement readout. The binary-tree geometry gives rise to soft-clamped modes^37^, which exhibit exceptionally high quality factors. Although this integrated platform is well-suited for optomechanical readout, it is not exploited for that purpose here. Instead, we focus on the NCF introduced by the proximity of the PhC cavities and characterize the *Q* of the OP modes using a free-space interferometer, as in the previous section; no light is coupled into the PhC cavities to avoid any optomechanical back action.

We fabricate two families of devices: ‘integrated’ devices in which the string is flanked by PhC cavities at a gap *d* ranging from 150 to 600 nm and ‘reference’ devices without any PhC cavities (*d* = ∞). The devices studied throughout this work feature binary-tree resonators of varying designs, whose geometric parameters are listed in Supplementary Table 2. Figure 3d shows ringdown measurements for the fundamental modes of two devices fabricated on the same chip with identical binary-tree string designs: an integrated device and a reference device. According to the simulations, this mode is expected to occur at 160 kHz, with *Q* = 5 × 10^7^ and effective mass of *m*_eff_ = 7.6 pg. Although good agreement is observed for the reference device, the integrated device with *d* = 200 nm has about ten times lower *Q*, 4.7 × 10^6^. As shown in Fig. 3e, a calibrated measurement of the thermal force power spectral density, ${S}_{{\rm{F}}}^{\mathrm{th}}$ (see Methods and Extended Data Fig. 2 for the experimental details) also shows an increase in ${S}_{{\rm{F}}}^{\mathrm{th}}$ from 3 to 21 aN^2^ Hz^−1^, consistent with the increase in the mechanical linewidth according to the ringdown results. We further characterize an ensemble of 77 reference and integrated devices with varying gaps, all on the same chip. The data shown in Fig. 3f show a systematic reduction of the *Q* for shorter gaps. In binary-tree resonators, in addition to the fundamental mode, the higher-order OP modes are also soft-clamped and possess high quality factors. We study reference and integrated devices with *d* = 200 nm to compare the first four OP modes. FEM simulations of the mode shapes and the outcome of the measurements are shown in Fig. 3g. For the fundamental mode the contrast between the reference and integrated devices is reproduced. Although the third-order mode has a similar behaviour, even-order modes that have a node at the centre experience no considerable *Q* reduction. This observation confirms that the additional loss channel arises from a local interaction with the PhC cavity.

**Fig. 4:**
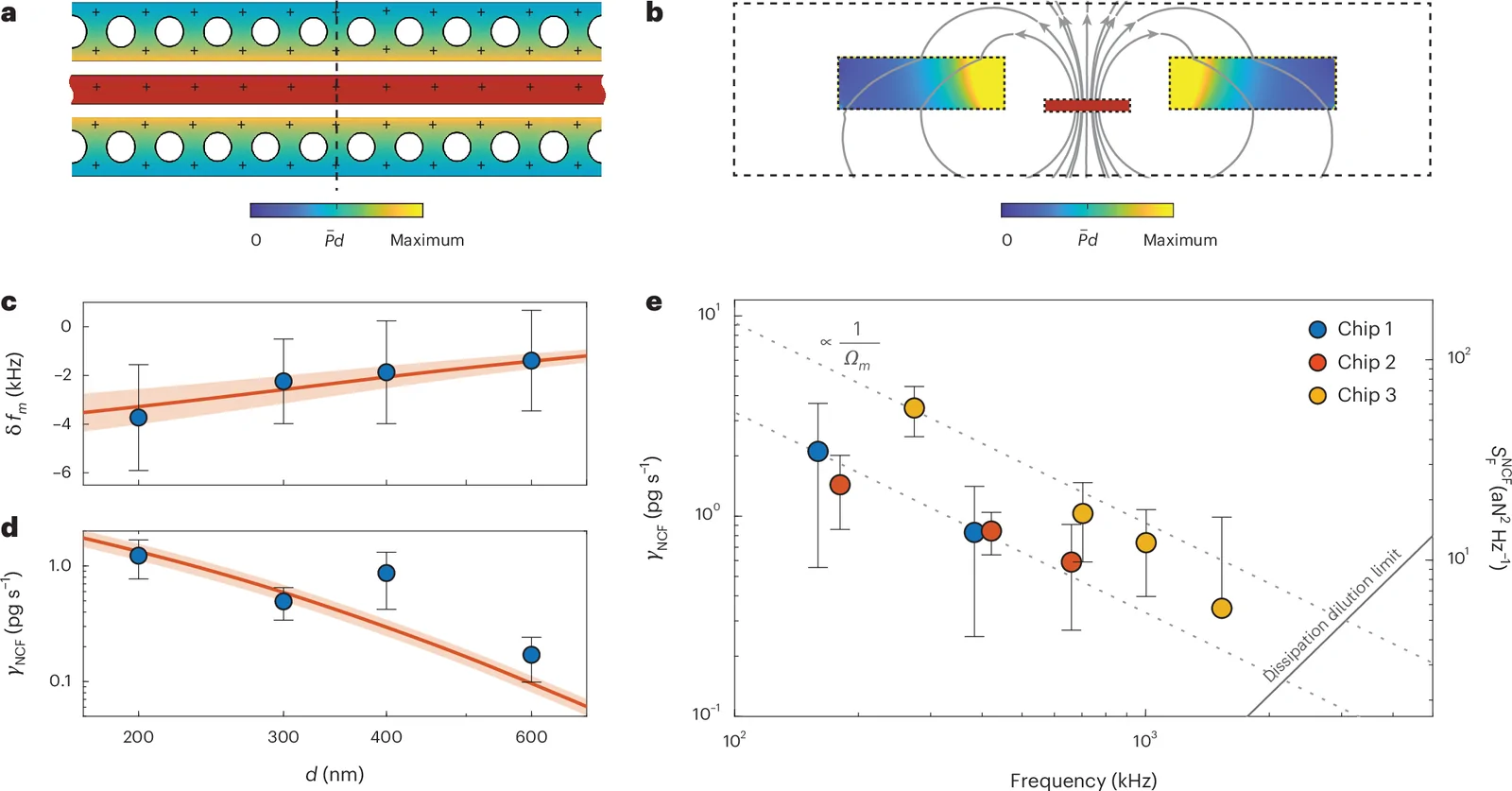
Modelling NCF in a nano-optomechanical system. **a**, Simulation of the dissipated power density, ${\bar{p}}_{{\rm{d}}}$, in the PhC cavities due to NCF. The simulation region shown here is a 7-μm-long segment of the central part of Fig. 3c. The plus marks indicate the static charge distribution. The colour map shows the dissipated power density. The dashed line is where the cross-section in **b** is shown from. **b**, Dissipated power density in the PhC cavities shown in the cross-section of the geometry in **a**. The solid grey lines show the field lines corresponding to the electric field variation **g**. The dashed outlines indicate the charged surfaces. **c**, Frequency shift of the fundamental modes of integrated devices with varying gaps with respect to the average values for the reference devices. The blue circles show the average value for devices with same gap. The error bars correspond to the standard deviation of the ensemble. The solid orange line shows the FEM simulations with the charge density and loss tangent obtained from the fits. Shaded area corresponds to the 95% confidence interval of these values. **d**, NCF-limited damping coefficients for the same devices in **c**. **e**, Inferred NCF damping coefficient for odd-order modes of integrated devices with different mechanical designs on three different chips. The circles correspond to the ensemble average value for modes with identical parameters, with colours corresponding to the chip number. The error bars correspond to the standard deviation. The dotted grey lines are guides to the eye for 1/*Ω*_*m*_ scaling. The right axis corresponds to the same values calibrated in the force noise power spectral density units. The grey solid line is the lower limit of ${S}_{{\rm{F}}}^{\mathrm{th}}$ for soft-clamped modes. Source data

To model NCF in this system, we consider a uniform charge distribution on all the surfaces of the device. This includes the surfaces of both the string and the PhC cavities (Fig. 4a,b). A key observation supporting the idea that the string and PhCs carry charges of the same sign is the gap-dependent frequency shift seen in the devices of Fig. 3f. In Fig. 4c, we show this frequency shift relative to the average of the reference devices. The observed negative frequency shift at smaller gaps arises from Coulomb repulsion between similar charges on the string and the PhC. We calculate the frequency shift as a function of the gap using an FEM model (Supplementary Information) and fit the model to the data, using the surface charge density as the only free parameter. The fit, shown in Fig. 4c, yields *ρ*_*e*,s_ = 8.8 ± 0.9 × 10^9^ e cm^−^^2^, in good agreement with the value obtained in the previous section. Using this charge density, we use our NCF FEM model to compute the dissipated power density in the PhC cavities (Fig. 4a,b). For better data analysis and comparison with this model, for every mechanical mode we introduce the experimentally inferred NCF damping coefficient as

5 $${\widetilde{\gamma }}_{\mathrm{NCF}}={m}_{\mathrm{eff}}\left({\varGamma }_{m}^{d}-\langle {\varGamma }_{m}^{\infty }\rangle \right),$$

where *m*_eff_ is the effective mass of the mode, given by the FEM simulation, ${\varGamma }_{{m}}^{{d}}$ is the damping rate of the mode for the integrated device with gap *d* and $\langle {\varGamma }_{{m}}^{{\infty }}\rangle$ is the average value of the damping rates of the same mode in the reference devices of the same chip. For the data in Fig. 3f, we plot ${\widetilde{\gamma }}_{\mathrm{NCF}}$ versus gap in Fig. 4d. Using the charge density extracted from the frequency shift analysis, we fit the FEM model to the data, with the dielectric loss tangent of Si_3_N_4_, $\tan {\delta }_{{\mathrm{Si}}_{3}{{\rm{N}}}_{4}}$, as the only free parameter. The result is shown in Fig. 4d and yields $\tan {\delta }_{{{\rm{Si}}}_{3}{{\rm{N}}}_{4}}=9\pm 3\times 1{0}^{-5}$. We emphasize that making this measurement using electrical circuits is not straightforward due to ohmic losses.

The inferred ${\widetilde{\gamma }}_{\mathrm{NCF}}$ allows us to extract the frequency dependence of NCF using the higher order modes, despite differences in their effective masses. Figure 4e shows ${\widetilde{\gamma }}_{\mathrm{NCF}}$ for the odd-order modes of three different mechanical designs across three chips, all with *d* = 200 nm. All mode shapes vary slowly near the PhCs, allowing direct comparison of modes from different chips. We observe a 1/*Ω*_*m*_ trend for devices on each chip, consistent with theoretical expectations (see Methods and Extended Data Fig. 3 for a discussion). Devices on chip 3 exhibit higher NCF than chips 1 and 2; this chip was processed in a separate fabrication run involving hydrofluoric acid exposure. We attribute this difference to variations in surface charge density, which can depend on exposure to different pH environments^35^. To contextualize the values in Fig. 4e, we convert them to force noise as ${S}_{{\rm{F}}}^{\mathrm{NCF}}=4{k}_{{\rm{B}}}T{\widetilde{\gamma }}_{\mathrm{NCF}}$, representing a limit for force sensing with these devices. Although increasing the mode frequency reduces the impact of NCF, it comes with a trade-off: soft-clamped modes achieve high *Q* through dissipation dilution, but this enhancement has a theoretical upper limit—the clampless limit^10^—that scales as $1/{\varOmega }_{m}^{2}$ with mode frequency, meaning higher-frequency modes are ultimately more constrained by bending loss. Using an assumption for the effective mass (Methods) we obtain a lower bound for the ${S}_{{\rm{F}}}^{\mathrm{th}}$ of soft-clamped modes, also shown in Fig. 4e. Given the amount of charge on the devices, there is an optimal frequency at which the sensor has the best sensitivity.

## Conclusion

We have shown that ultrahigh-*Q* nanomechanical resonators are sensitive to NCF when they are placed near other objects. The presence of static charges on these devices gives rise to NCF-induced dissipation coefficients as low as 10^−16^ kg s^−1^. This value is about two to three orders of magnitude lower than what has been measured previously^27,30^, yet it limits the *Q* of the resonators used in this work. Although we made simplifying assumptions about the charge distribution, our methods allow precise modelling of NCF in arbitrary geometries with arbitrary charge distributions, which can be characterized using standard techniques such as Kelvin probe force microscopy. In a broader context, our study highlights the challenges that static charges pose for microfabricated devices used in sensitive experiments. Effects similar to NCF have been observed in other electric-field-sensitive experiments such as trapped ions^38^ and Ryberg atoms^39^. With growing interest in scaling up hybrid quantum systems for computing, simulation and sensing, microfabricated devices are inevitable. Understanding the limitations imposed by imperfections such as trapped charges is therefore essential. Conversely, our work points to opportunities for new functionalities: static charges can enable nanomechanical resonators to couple to electric fields and other quantum systems with electronic degrees of freedom, such as ions and Rydberg states of neutral atoms. Moreover, the level of dissipation measured here at room temperature is on par with the state-of-the-art cryogenic AFM system^13^. With this regime of sensitivity and further development, our techniques can probe material properties in new ways.

## Methods

### NCF for an infinitesimally thin string

We calculate the NCF damping coefficient for a one-dimensional tensioned string interacting with a substrate. The schematic is shown in Extended Data Fig. 1a. The string has length *L* and a linear charge density of *ρ*_*e*,l_. The motion of the beam is given by a mode shape *U*(*x*) (motion along the *z* axis). Generally, for situations where the electric energy dissipated within the volume of the resonator itself can be neglected, **g** is equivalent to the electric field generated by a polarization density ${\bf{P}}({\bf{r}})={\rho }_{e}({\bf{r}}){{\bf{U}}}_{m}({\bf{r}})$ (Supplementary Information). Due to the zero volume of the string, to calculate the field variation **g** we can use this approximation and replace the linear charge density, with a linear polarization density ${{\bf{P}}}_{{\rm{l}}}(x)={\rho }_{e,{\rm{l}}}U(x){{\widehat{\mathbf{z}}}}$. Moreover, for modes where $d\ll | {U}^{{\prime} }(x){| }^{-1}$ we can approximately reduce the problem to two dimensions in the cross-section and compute the field for an infinite line (Supplementary Information). We consider two different types of substrates and compute the NCF-limited quality factors for each case.

#### NCF due to a conductive substrate with a thin oxide layer

Even for high resistivity silicon, the characteristic time constant for the charges to decay is given by *ϵ*/*σ* ≈ 10^−8^ s, rendering silicon as a conductor for our frequency range. Hence, we have to treat the silicon substrate as a conductor in our models. For modelling the effect of the native silicon oxide layer, the schematic is shown in Extended Data Fig. 1b. For a thin oxide layer (*Δ* ≪ *d*), we can neglect the effect of the dielectric layer on the electric field and compute it as if the string was in front of a conductor. Hence, the electric field variation is given by the field generated by the original dipole density ${{\bf{P}}}_{{\rm{l}}}(x)$ in addition to its image $-{{\bf{P}}}_{{\rm{l}}}(x)$ at *z* = *Δ* + *d*. Moreover, we approximate the field within the layer to be uniform and equal to the field generated by the two dipoles at the boundary, reduced by ${\epsilon }_{r}^{{\prime} }$. The resulting field within the dielectric only has a *z* component and is given by

6 $${\bf{g}}({\bf{r}},\omega ;U(x))\approx \frac{{\rho }_{e,{\rm{l}}}}{{\uppi }{\epsilon }_{0}{\epsilon }_{r}}\frac{\left({y}^{2}-{d}^{\,2}\right){{\widehat{\mathbf{z}}}}}{{\left({y}^{2}+{d}^{2}\right)}^{2}}U(x).$$

To calculate *γ*_NCF_, the NCF damping for a harmonic mode of the string with frequency *Ω*_*m*_, we use equation (2) and integrate $| {\bf{g}}{| }^{2}$ within the oxide layer

7 $${\gamma }_{\mathrm{NCF}}=\frac{{\rho }_{e,{\rm{l}}}^{2}{\epsilon }_{r}^{{\prime\prime} }}{| {\uppi }{\epsilon }_{0}{\epsilon }_{r}{| }^{2}{\Omega }_{m}}\times {\int }_{0}^{\varDelta }{\int }_{-\infty }^{\infty }{\int }_{0}^{L}{\rm{d}}x{\rm{d}}y{\rm{d}}z\frac{{\left({y}^{2}-{d}^{\,2}\right)}^{2}}{{\left({y}^{2}+{d}^{2}\right)}^{4}}| U(x){| }^{2}.$$

The integration along the *x* axis is evaluated using the definition of the effective mass. Given that the mode *U*_*n*_(*x*) is normalized to its maximum the effective mass is defined as ${m}_{\mathrm{eff}}={\rho }_{m,{\rm{l}}}{\int }_{0}^{L}{\rm{d}}x| U(x){| }^{2}$, where *ρ*_*m*,l_ is the linear mass density. Carrying out the integration in the *yz* plain yields

8 $${\gamma }_{\mathrm{NCF}}=\frac{{m}_{\mathrm{eff}}{\rho }_{e,{\rm{l}}}^{2}\varDelta }{4{\uppi }{\epsilon }_{0}{d}^{3}{\rho }_{m,{\rm{l}}}}\frac{\tan \delta ({\varOmega }_{m})}{{\epsilon }_{r}^{{\prime} }({\varOmega }_{m}){\varOmega }_{m}}.$$

Calculating the damping rate, *Γ*_NCF_ = *γ*_NCF_/*m*_eff_, and the quality factor yields

9 $${Q}_{\mathrm{NCF}}=\frac{4{\uppi }{\epsilon }_{0}{d}^{\,3}{\rho }_{m,{\rm{l}}}}{{\rho }_{e,{\rm{l}}}^{2}\varDelta }\frac{{\epsilon }_{r}^{{\prime} }({\varOmega }_{m}){\varOmega }_{m}^{2}}{\tan \delta ({\varOmega }_{m})}.$$

To obtain the numerical estimates for *Q*_NCF_ shown in Fig. 1e, we have considered the dielectric permittivity and loss tangent of the SiO_2_ substrate to be 3.9 and 10^−3^, respectively^40^. We have also retrieved the designs and the chips from refs. ^4,11,12^, measured the distance *d* using an optical microscope and computed the corresponding *Q*_NCF_ for each geometry.

#### NCF due to a semi-infinite dielectric substrate

The schematic for the semi-infinite lossy dielectric substrate is shown in Extended Data Fig. 1c. The *z* > 0 space is filled by the dielectric with relative permittivity *ϵ*_*r*_(*ω*). Using an elementary method of image charges, we calculate the field within the dielectric (*z* > 0) by replacing ${{\bf{P}}}_{l}(x)$ with $(1+\xi ){{\bf{P}}}_{l}(x)$. Following these steps, we find the electric field variation for *z* > 0 as

10 $${\bf{g}}({\bf{r}},\omega ;U(x))=\frac{(1+\xi \,){\rho }_{e,{\rm{l}}}}{2{\uppi }{\epsilon }_{0}{\epsilon }_{r}}\times \frac{2y(z+d){{\widehat{\mathbf{y}}}}+\left[{(z+d)}^{2}-{y}^{2}\right]{{\widehat{\mathbf{z}}}}}{{\left[{y}^{2}+{(z+d)}^{2}\right]}^{2}}U(x).$$

To calculate *γ*_NCF,*n*_, the NCF damping for a harmonic mode of the string *U*_*n*_, we use equation (2).

11 $${\gamma }_{\mathrm{NCF},n}=\frac{| 1+\xi {| }^{2}{\rho }_{e,{\rm{l}}}^{2}}{4{{\uppi }}^{2}{\epsilon }_{0}| {\epsilon }_{r}{| }^{2}}\frac{{\epsilon }_{r}^{{\prime\prime} }}{{\varOmega }_{n}}\times {\int }_{0}^{\infty }{\int }_{-\infty }^{\infty }{\int }_{0}^{L}{\rm{d}}x{\rm{d}}y{\rm{d}}z\frac{| {U}_{n}(x){| }^{2}}{{\left[{y}^{2}+{(z+d)}^{2}\right]}^{2}}.$$

Carrying out the integrations similar to the previous case, one obtains

12 $${\gamma }_{\mathrm{NCF},n}=\frac{{m}_{\mathrm{eff},n}{\rho }_{e,{\rm{l}}}^{2}}{8{\uppi }{\epsilon }_{0}{d}^{2}{\rho }_{m,{\rm{l}}}}\frac{\mathrm{Im}[\xi ({\varOmega }_{n})]}{{\varOmega }_{n}},$$

where we have used the relation $\mathrm{Im}[\xi ({\varOmega }_{n})]\approx \frac{2{\epsilon }_{r}^{{\prime\prime} }}{{(1+{\epsilon }_{r}^{{\prime} })}^{2}}$ with the assumption ${\epsilon }_{r}^{{\prime\prime} }\ll {\epsilon }_{r}^{{\prime} }$. Calculating the damping rate, *Γ*_NCF,*n*_ = *γ*_NCF,*n*_/*m*_eff,*n*_, and the quality factor yields equation (3).

### Measurement and calibration techniques

The measurement apparatus used to perform the ringdown and thermal noise measurements is the same as the one used in refs. ^4,12^. For completeness, we show a simplified schematic of the setup in Extended Data Fig. 2a. The sample is placed in a vacuum chamber with *P* < 10^−8^ mbar and room temperature. The mechanical motion is read out using a balanced homodyne interferometer, where laser light from a 780-nm diode laser is focused on the sample using a microscope objective. The reflected light is then interfered with a local oscillator and detected using a balanced photodetector. The optical path length difference and conequently the local oscillator–signal phase difference is stabilized by feeding back the low frequency (that is, <300 Hz) component of the homodyne signal to cascaded double acoustic–optic modulators (AOMs) in the local oscillator path, using a technique described in ref. ^12^. The high frequency component contains the mechanical motion signal and is used for the ringdown and thermal noise measurements. Using a lock-in amplifier unit (Zurich Instruments UHFLI), the signal is demodulated at the mechanical frequency and the amplitude of motion (that is, *R* in Fig. 3d) is obtained which is used for the ringdown measurements. The mechanical mode is excited using a piezoelectric actuator attached to the sample holder. To avoid optical back-action effects, the ringdowns are done stroboscopically using a shutter placed in the optical path. In our measurements the typical open to close duty cycle of the shutter is about 1:10. The calibrated thermal force measurement, shown in Fig. 3e, is performed using the technique described in ref. ^4^. The power spectral density of the homodyne signal is obtained using a spectrum analyser and it is calibrated in units of displacement spectral density, that is, m^2^ Hz^−1^ using the method described in ref. ^4^. To avoid the distortion of the spectrum due to slow drifts in the mechanical frequency, the mechanical mode is feedback cooled using the piezoelectric actuator by broadenning the mechanical linewidth from its intrinsic value of *Γ*_*m*_ to a value of *Γ*_eff_. The thermomechanical spectrum shown in Extended Data Fig. 2b is acquired under this condition. The measured spectrum is given by ${S}_{x}^{\mathrm{meas}}(\omega )={S}_{{\rm{F}}}^{\mathrm{th}}| {\chi }_{\mathrm{eff}}(\omega ){| }^{2}+{S}_{x}^{\mathrm{imp}}$, where

13 $${\chi }_{\mathrm{eff}}(\omega )=\frac{1}{{m}_{\mathrm{eff}}\left({\varOmega }_{m}^{2}-{\omega }^{2}-i\omega {\varGamma }_{\mathrm{eff}}\right)},$$

is the effective mechanical susceptibility under feedback cooling, which is fully known since the effective mass is also known from the FEM simulations, and *Γ*_eff_ is obtained from a Lorentzian fit to the measured spectrum. ${S}_{x}^{{\rm{imp}}}$ is the imprecision noise floor of the measurement. We obtain the measured thermal force spectrum by deviding the measured displacement spectrum by the effective susceptibility, which yields ${S}_{{\rm{F}}}^{\mathrm{meas}}={S}_{{\rm{F}}}^{\mathrm{th}}+{S}_{{\rm{F}}}^{\mathrm{imp}}$. The resulting spectra are shown in Extended Data Figs. 2c and 3e, in all of which the flat component corresponds to ${S}_{{\rm{F}}}^{\mathrm{th}}$, and the rising branch corresponds to ${S}_{{\rm{F}}}^{\mathrm{imp}}$.

### Ruling out other damping mechanisms

We discuss five mechanisms that could explain a distance-dependent damping in both the uniform strings and the nano-optomechanical system. We show that none of them can fully explain our observations.

#### Gas and squeeze-film damping

Although the samples are tested under high vacuum (*P* < 10^−8^ mbar), the small gaps can result in enhancement of gas damping due to squeeze-film damping. However, squeeze-film gas damping results in a frequency-independent damping rate^41,42^, different from our observations.

#### Local surface contamination

Local surface contamination means reduction of the intrinsic mechanical *Q* in a local area on the nano-beam. For example, this could be a result of the etching of the PhCs in the vicinity of the beam. Such a mechanism could in principle explain the different behaviour of modes with different parities. To study this effect, we use a modified version of the FEM simulations used for estimating the dilution factor for tensioned and high-aspect-ratio resonators^43^. These simulations are based on evaluating the kinetic and bending energies, *W*_kin_ and *W*_bend_, for a given mode and then computing the quality factor as *Q* = *W*_kin_/*W*_bend_. For a two-dimensional resonator (in the *xy* plane) and an OP mode with frequency *Ω* and mode shape *U*(*x*, *y*), these energies are given by

14 $${W}_{\mathrm{kin}}=\frac{1}{2}{\rho }_{m}h{\varOmega }^{2}{\int }_{S}{\rm{d}}x{\rm{d}}yU{(x,y)}^{2},$$

15 $$\begin{array}{l}{W}_{\mathrm{bend}}={\frac{E{h}^{3}}{24(1-{\nu }^{2})}}\displaystyle \int _{S}{\rm{d}}x{\rm{d}}y\phi (x,y)\\ \times \left[{({U}_{xx}+{U}_{yy})}^{2}+2(1-\nu )\left({U}_{xy}^{2}-{U}_{xx}{U}_{yy}\right)\right],\end{array}$$

where *ρ*_*m*_ is the mass density, *h* is the thickness, *E* is the Young’s modulus, *ν* is the Poisson’s ratio of the resonator and *U*_*ij*_ corresponds to partial derivatives of *U*(*x*,*y*). The integrations are carried over *S*, the area of the resonator. *ϕ*(*x*, *y*) defines the local mechanical loss angle of the resonator where in the usual cases, it is constant and given by the inverse of the material’s intrinsic quality factor $\phi (x,y)={Q}_{{\rm{int}}}^{-1}$.

For the resonator design used to take the data in Fig. 3g, we use this FEM simulation to estimate the effect of local contamination. In a region on the resonator nearby the PhC’s location (Extended Data Fig. 3a), we set *ϕ* higher than the usual value so that we reproduce the observed reduced quality factor. We compute the *Q* for the first few OP modes and compute the added damping coefficient compared to the fully clean case (similar to equation (5)). We show these values for the modes with an anti-node in the centre in Extended Data Fig. 3d. We observe an increasing frequency dependence for the added damping coefficient which is unable to explain the experimental observations.

#### Mechanical coupling to the PhC

Another mechanism that can introduce a distance-dependent loss in the nano- optomechanical system is coupling of the high-*Q* mode to the low-*Q* mechanical modes of the PhC cavity^22^. Extended Data Fig. 3b shows an optical microscope image of the PhC cavities and the string suspended between them. One can clearly see that the cavities are also suspended and can support mechanical modes. We show an FEM simulation of the fundamental mode of the PhC cavity in Extended Data Fig. 3c. This mode has the frequency of 2.1 MHz and we also observe it in the experiments. It can in principle couple to the modes of the binary-tree resonator through multiple mechanisms such as electrostatic or van der Waals forces. Generally, for two mechanical modes with frequencies *Ω*_1(2)_, damping rates *Γ*_1(2)_ and effective masses *m*_1(2)_, that are coupled by a spring constant *k*_c_, the additional damping for mode 1 is given by

16 $$\delta {\varGamma }_{1}=\frac{{k}_{{\rm{c}}}^{2}}{{m}_{1}{m}_{2}}\frac{{\varGamma }_{2}}{{\left({\varOmega }_{2}^{2}-{\varOmega }_{1}^{2}\right)}^{2}+{\varGamma }_{2}^{2}{\varOmega }_{1}^{2}},$$

having a resonance-like frequency dependence. For the measured parameters for the two mechanical modes, we set *k*_c_ to a value to reproduce the additional damping for the fundamental mode of the binary-tree resonator at 160 kHz. The result is shown in Extended Data Fig. 3d, clearly inconsistent with our observed frequency scaling.

#### Finite electrical conductivity

Throughout our work, we have assumed that the dissipation within the dielectrics (SiO_2_ and Si_3_N_4_) is purely due to the dielectric loss (that is the imaginary part of the permittivity). However, having a finite charge mobility and consequently electrical conductivity of *σ* ≠ 0, can also introduce NCF. For a conductivity, small enough that does not disturb the electric field distribution at the mechanical frequency (that is, *σ* ≪ *ϵ*_0_*ϵ*_*r*_/*Ω*_*m*_) it can be shown that the NCF coefficient arising from conductive losses is given by (Supplementary Information)

17 $${\gamma }_{\mathrm{NCF}}^{\mathrm{cond}}=\int \,{d}^{\,3}r\,| {\bf{g}}({\bf{r}},{{\varOmega }_{m}};{{\bf{U}}}_{m}({\bf{r}})){| }^{2}\frac{\sigma ({\bf{r}},{\varOmega }_{m})}{{\varOmega }_{m}^{2}}.$$

For a frequency-independent conductivity, evidently this form of NCF cannot explain our observed frequency scaling of 1/*Ω*_*m*_. The only possible way in which the conductive losses give rise to 1/*Ω*_*m*_ is that the conductivity has a strictly linear frequency dependence in the 100 kHz to 1 MHz range. For SiO_2_, previous measurements of the conductivity of thermally grown oxide on silicon^44^, demonstrate ${\sigma }_{{\mathrm{SiO}}_{2}}\approx 1{0}^{-14}\,{\Omega }^{-1}\,{{\rm{m}}}^{-1}$ at d.c. and a weak frequency dependence. For stoichiometric silicon nitride a similar value has been reported^45^. With these values for the conductivity, the contribution to the loss tangent is on the order of 10^−9^, much smaller than our estimated values.

#### Fundamental thermal-electrodynamic damping

In the absence of static charges, the finite thermal fluctuations of the electromagnetic field gives rise to the ultimate form of NCF^46^. This mechanism that often goes by different names (for example, vacuum friction, van der Waals friction and Casimir friction) is a direct consequence of the fluctuation–dissipation theorem for the electromagnetic field in the presence of lossy dielectrics. When two dielectrics are in relative motion, the retardation of the fluctuating electromagnetic field in conjunction with the force exerted on the dielectrics through Maxwell’s stress tensor, gives rise to friction forces. Estimating the damping force for arbitrary geometries is complicated, but there is an analytical solution for the case of two semi-infinite bodies^47^. We estimate the damping coefficients, ${\gamma }_{{\rm{NCF}}}^{{\rm{th}}}$, for silicon nitride bodies for both parallel and perpendicular relative motions. As shown in Extended Data Fig. 3e, even for distances of tens of nanometres, ${\gamma }_{{\rm{NCF}}}^{{\rm{th}}}$ is orders of magnitude smaller than our observed values. Moreover, it has been shown that for a mechanical oscillator, this form of damping does not depend on the harmonic potential of the oscillator (that is, ${\gamma }_{{\rm{NCF}}}^{{\rm{th}}}$ is frequency independent)^48^.

### Dissipation–dilution limit to force sensitivity

For a string with thickness *h*, stress *σ* and mass density *ρ*_*m*_, the ultimate limit for the *Q* at a given frequency *Ω* from dissipation–dilution (also known as the clampless limit) is given by^10^

18 $$\frac{{Q}_{m}^{\mathrm{cl}}}{{Q}_{\mathrm{int}}}\le \frac{12{\sigma }^{2}}{{\rho }_{m}E{h}^{2}{\varOmega }^{2}},$$

where *E* is the Young’s modulus. To obtain a limit for the force sensitivity at a given string width, *w*, we set the effective mass to the value for the fundamental mode of a uniform string with frequency *Ω*, ${m}_{\mathrm{eff}}^{\mathrm{cl}}=\frac{{\rho }_{m}wh}{2\varOmega }\sqrt{\frac{\sigma }{{\rho }_{m}}}$. This is the minimal value for a given thickness and width of the string, and for soft-clamped modes, this value is up to a factor 2 larger. Together, the limit of force sensitivity is given by

19 $${S}_{{\rm{F}}}^{\mathrm{th},\mathrm{cl}}=4{k}_{{\rm{B}}}T\frac{E{h}^{3}w}{24{Q}_{\mathrm{int}}}{\left(\frac{{\rho }_{m}}{\sigma }\right)}^{\frac{3}{2}}{\varOmega }^{2}.$$

## Online content

Any methods, additional references, Nature Portfolio reporting summaries, source data, extended data, supplementary information, acknowledgements, peer review information; details of author contributions and competing interests; and statements of data and code availability are available at https://doi.org/10.1038/s41567-026-03350-z.

## Supplementary information

Supplementary Information Supplementary Sections 1–10, Figs. 1–14, and Tables 1 and 2, including details of the theory, numerical simulations and experimental techniques.

Peer Review File

## Source data

Source Data Fig. 1 Zip file containing data plotted in the subpanels of the figure in either excel or .txt format.

Source Data Fig. 2 Zip file containing data plotted in the subpanels of the figure in either excel or .txt format.

Source Data Fig. 3 Zip file containing data plotted in the subpanels of the figure in either excel or .txt format.

Source Data Fig. 4 Zip file containing data plotted in the subpanels of the figure in either excel or .txt format.

## Data Availability

All the raw data used in this work, including the ringdown measurements and thermomechanical spectra, and the codes used for the analysis, running the FEM simulations and generating Figs. 1–4, are available via Zenodo at https://doi.org/10.5281/zenodo.18741382 (ref. ^49^). Source data are provided with this paper.
