# Supplementary material for: Non-contact friction in ultracoherent nanomechanical resonators near dielectric materials
Source: Nat Phys. 2026 Jul 7;22(9):1506–12. doi: 10.1038/s41567-026-03350-z (PMC13558075; doi:10.1038/s41567-026-03350-z)
Supplement: Supplementary file 1 [file 41567_2026_3350_MOESM1_ESM.pdf]

nature physics

Supplementary information https://doi.org/10.1038/s41567-026-03350-z

Non-contact friction in ultracoherent nanomechanical resonators near dielectric materials

In the format provided by the authors and unedited

CONTENTS

I. Detailed theory of noncontact friction in deformed geometries 2
A. General properties of $\vec{g}$ 2
B. Green's function method for moving charges 6
C. Dissipated power and stored energy 7
D. Effect of finite conductivity 9
E. Forces on the moving bodies 10

II. Noncontact friction for an infinitesimally thin string 11
A. Semi-infinite dielectric slab: explicit approach 11
B. Semi-infinite dielectric slab: out-of-phase force 13
C. A finite dielectric slab on a conductor 13
D. NCF damping in ultracoherent resonators above Si substrates 15

III. General 3D to 2D reduction 16

IV. Finite element model for computation of NCF coefficient 17
A. Uniform beams above a dielectric surface 19
B. Integrated nano-optomechanical system 20

V. Fabrication processes 22
A. Uniform strings on oxide samples 22
B. Integrated nano-optomechanical devices 24

VI. Supplementary data for the width dependence 24

VII. Signatures of slow charge dynamics 26
A. Slow drifts of the NCF-limited damping in strings 26
B. Mechanical frequency fluctuations 27

VIII. Geometry parameters for the binary-tree resonators 29

IX. Overview of force sensitivity and dissipation of nanomechanical resonators. 29

X. Attempts for mitigation of NCF 31

A. In situ thermal annealing 31
B. Ultraviolet annealing 31

References 33

I. DETAILED THEORY OF NONCONTACT FRICTION IN DEFORMED GEOMETRIES

The mathematical details of the computation of the NCF damping are discussed in this section. In particular, the properties of the electric field variation $\vec{g}$ and the methods for calculating it are discussed. We compute the dynamical effects on the moving bodies, meaning both frequency shifts and damping coefficients using two methods. The first is the same as the one discussed in the main text, i.e. computing the energy within the dielectrics. The second is to calculate the forces on the moving bodies.

The previous theories developed for computing the NCF coefficient for AFM cantilevers, are based on modeling the interaction of a moving point charge with a lossy semi-infinite dielectric [1, 2]. The approach taken in these works is based on using the fluctuation-dissipation theorem to find the damping coefficient via finding the linear response between the position of the point charge and electric field induced by the dielectric [1]. In our work, we generalize these theories to arbitrary geometries and charge distributions and computing the damping coefficient through direct calculation of the dissipated energy.

A. General properties of $\vec{g}$

First of all, we limit the discussions in this paper to the situations where we can make the quasi-electrostatic approximation (i.e. size of the system $\ll \Omega/c$). Situations beyond this approximation are irrelevant to nanomechanics. The static electric field, for the undeformed geometry, obeys the equations,

$$\nabla \cdot \left[\epsilon_r(\vec{r})\vec{E}_0(\vec{r})\right] = \frac{\rho_e(\vec{r})}{\epsilon_0}, \tag{1a}$$

$$\nabla \times \vec{E}_0(\vec{r}) = 0, \tag{1b}$$

as well as the usual boundary conditions for dielectric media, given by

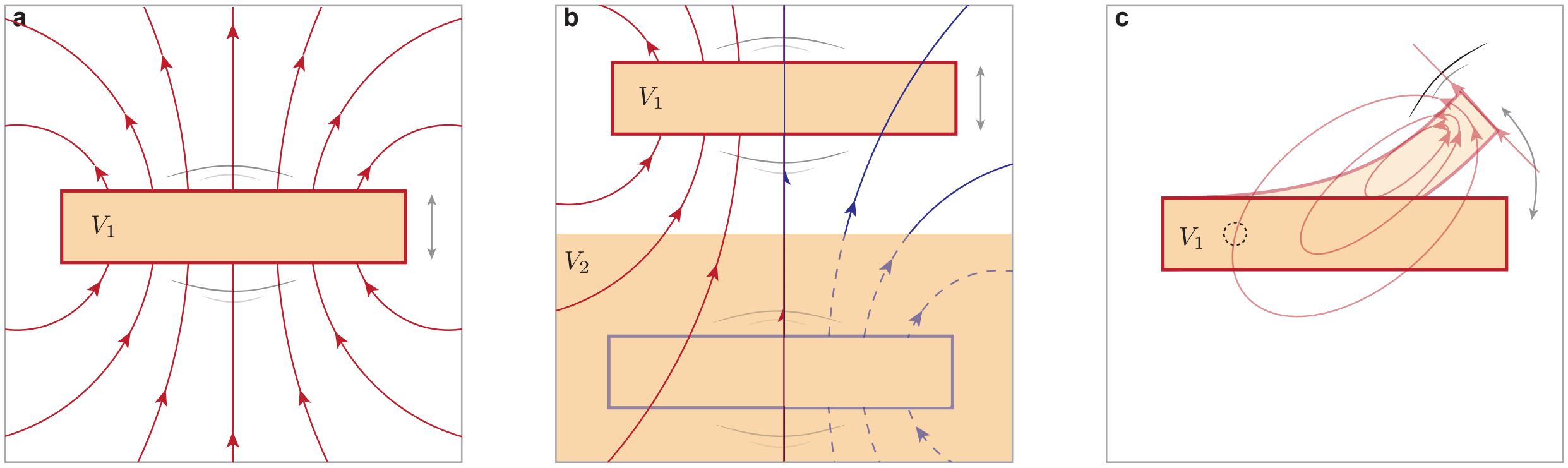

FIG. 1. **Illustration of the impact of relative motion on NCF a** Here an isolated dielectric with volume V_1 with charges on its surface (marked by red lines) has a vertical center of mass motion. In the laboratory frame, the field variation marked by the red streamlines is generated while at the reference frame of the dielectirc the field variation is zero as there is no motion. **b** In this case the charged dielectric V_1 moves above another dielectric with volume V_2. In the reference frame of V_2, the red field is generated, which is the one discussed in the main text and causes dissipation within V_2. However, in the reference frame of V_1, while the field variation due to the charges on the resonator (red charges) is zero, the image charges in V_2 are in motion and generate the field variation shown by the blue lines within V_1. This field causes dissipation within the resonator. **c** Here the resonator is again isolated but instead of a center of mass motion, it is deformed (similar to a cantilever). In this scenario the field variation within the resonator also depends on the point of view. For example at an observation point towards the left side of the resonator (marked by the dashed circle) the motion of the charges on the other side of the resonator generate a field variation which generates loss within the resonator. This type of dissipation is present in any mechanical resonator that is charged, regardless of being isolated or not.

$$\left(\epsilon_r^{(2)}\vec{E}_0^{(2)} - \epsilon_r^{(1)}\vec{E}_0^{(1)}\right) \cdot \vec{n} = \sigma_e, \tag{2a}$$

$$\left(\vec{E}_0^{(2)} - \vec{E}_0^{(1)}\right) \times \vec{n} = 0, \tag{2b}$$

where σ_e is the charge density at the boundary and (1) and (2) denote below and above it with respect to the normal vector $\vec{n}$. A deformation field $\vec{u}(\vec{r},t)$, displaces both the charges and the dielectric boundaries. For small displacements, one can find the variation of $\rho_e(\vec{r})$ and $\epsilon_r(\vec{r})$ perturbatively by taking the transformation $\vec{r} \to \vec{r} + \vec{u}(\vec{r},t)$. However, there is

an important subtlety here. The mechanical-dielectric dynamical effects (both dispersive and dissipative) occur only when there is a relative motion between the charges and the dielectrics (see Fig. 1). In other words, since the origin of the dynamical effects is the frequency dependent response of the dielectric, the field variation has to be calculated in the frame of reference of the dielectric. This makes the calculation of the field variation and eventually the dissipated power more complicated. To calculate the field variation at an observation point $\vec{r}$ in the reference frame of the dielectric at point $\vec{r}'$ we displace ρ_e and ϵ_r using the transformation $\vec{r} \to \vec{r} + \vec{\Delta}(\vec{r}, \vec{r}', t)$, where $\vec{\Delta}(\vec{r}, \vec{r}', t) = \vec{u}(\vec{r}, t) - \vec{u}(\vec{r}', t)$ is the relative displacement field.

In the main text, for simplicity, we have neglected the relative motion effects and defined $\vec{g}$ as the generalized derivative of the static electric field, $\vec{E}_0$, with respect to the motion of a particular mode

$$\vec{g}(\vec{r}, \Omega_m; \vec{U}_m) = \frac{\delta \vec{E}_0}{\delta u}, \tag{3}$$

where u is the generic amplitude of the motion for mode $\vec{U}_m$. Here we use a perturbative approach and derive the equations governing $\vec{g}$.

We decompose the displacement field using the vibrational eigenmodes of the geometry. These modes are the solutions of the relevant elasticity equations and are characterized by the frequencies $\Omega_{m,\alpha}$ and the mode shapes $\vec{U}_{m,\alpha}(\vec{r})$. The mode shapes are normalized to their maximum, i.e., $\max_{\vec{r}} |\vec{U}_{m,\alpha}(\vec{r})| = 1$. The displacement field is given by

$$\vec{u}(\vec{r}, t) = \sum_\alpha x_\alpha \vec{U}_{m,\alpha}(\vec{r}) e^{-i\Omega_{m,\alpha} t} + \text{c.c.}, \tag{4}$$

where $x_\alpha(t)$ is the amplitude of motion for the mode α. The relative displacement field also reads

$$\vec{\Delta}(\vec{r}, \vec{r}', t) = \sum_\alpha x_\alpha \left[\vec{U}_{m,\alpha}(\vec{r}) - \vec{U}_{m,\alpha}(\vec{r}') \right] e^{-i\Omega_{m,\alpha} t} + \text{c.c.}, \tag{5}$$

Now we expand the perturbation in the charge density, permittivity and the electric field to the first order in x_α

$$\rho_e(\vec{r}) \to \rho_e(\vec{r}) + \sum_\alpha \frac{\delta \rho_e}{\delta x_\alpha} x_\alpha e^{-i\Omega_{m,\alpha} t} + \text{c.c.}, \tag{6}$$

$$\epsilon_r(\vec{r}) \to \epsilon_r(\vec{r}) + \sum_\alpha \frac{\delta \epsilon_r}{\delta x_\alpha} x_\alpha e^{-i\Omega_{m,\alpha} t} + \text{c.c.}, \tag{7}$$

$$\vec{E}_0(\vec{r}) \to \vec{E}_0(\vec{r}) + \sum_\alpha \vec{g}(\vec{r}, \vec{r}'; \vec{U}_{m,\alpha}) x_\alpha e^{-i\Omega_{m,\alpha} t} + \text{c.c.}, \tag{8}$$

where

$$\frac{\delta \rho_e}{\delta x_\alpha} = \nabla \rho_e(\vec{r}) \cdot \left[\vec{U}_{m,\alpha}(\vec{r}) - \vec{U}_{m,\alpha}(\vec{r}\,')\right], \tag{9}$$

$$\frac{\delta \epsilon_r}{\delta x_\alpha} = \nabla \epsilon_r(\vec{r}) \cdot \left[\vec{U}_{m,\alpha}(\vec{r}) - \vec{U}_{m,\alpha}(\vec{r}\,')\right], \tag{10}$$

and $\vec{g}(\vec{r}, \vec{r}\,'; \vec{U}_{m,\alpha})$ is the field variation under displacements by mode $\vec{U}_{m,\alpha}$ at coordinate $\vec{r}$, observed in the reference frame moving at coordinate $\vec{r}\,'$. We have not written the dependency on the frequency for the sake of notation simplicity. Plugging these relations in Equations 1

$$\sum_\alpha \left\{ \nabla \cdot \left[\frac{\delta \epsilon_r}{\delta x_\alpha} \vec{E}_0(\vec{r}) + \epsilon_r(\vec{r}) \vec{g}(\vec{r}, \vec{r}\,'; \vec{U}_{m,\alpha}) \right] - \frac{1}{\epsilon_0} \frac{\delta \rho_e}{\delta x_\alpha} \right\} x_\alpha e^{-i\Omega_{m,\alpha} t} + \text{c.c.} = 0 \tag{11a}$$

$$\sum_\alpha \nabla \times \vec{g}(\vec{r}, \vec{r}\,'; \vec{U}_{m,\alpha}) x_\alpha e^{-i\Omega_{m,\alpha} t} + \text{c.c.} = 0, \tag{11b}$$

which yields the equations for the field variation under each eigenmode

$$\nabla \cdot \left[\epsilon_r(\vec{r}) \vec{g}(\vec{r}, \vec{r}\,'; \vec{U}_{m,\alpha}) \right] = \frac{1}{\epsilon_0} \frac{\delta \rho_e}{\delta x_\alpha} - \nabla \cdot \left[\frac{\delta \epsilon_r}{\delta x_\alpha} \vec{E}_0(\vec{r}) \right], \tag{12a}$$

$$\nabla \times \vec{g}(\vec{r}, \vec{r}\,'; \vec{U}_{m,\alpha}) = 0. \tag{12b}$$

As evident from these equations, $\vec{g}$ is equivalent to the electric field generated by two source charges on the right-hand side of Eq. (12a): the first one is a result of displacement of a non-uniform charge density and the second term is a result of deformation of a dielectric medium with non-uniform permittivity. Integration of these terms on the boundaries as well as Eq. (2a) give rise to three types of boundary sources:

1. A discontinuity of the charge density at a boundary gives rise to an equivalent surface charge density for $\vec{g}$. This surface density can be found by integrating Eq. (12a) over a small volume at the boundary. Using the definition Eq. (9) and the fact that $|\vec{U}_m|$ is finite, we find this surface density to be

$$\sigma_\alpha = \left(\rho_e^{(2)} - \rho_e^{(1)}\right) \left[\vec{U}_{m,\alpha}(\vec{r}) - \vec{U}_{m,\alpha}(\vec{r}\,')\right] \cdot \vec{n} \tag{13}$$

2. The second contribution is the displacement of the surface charges, σ_e, introduced in Eq. (2a), which gives rise to equivalent surface distributions of dipoles for $\vec{g}$. The corresponding dipole density at $\vec{r}$ on the boundary is given by

$$\vec{D}_{\alpha,e}(\vec{r}) = \sigma_e(\vec{r}) \left[\vec{U}_{m,\alpha}(\vec{r}) - \vec{U}_{m,\alpha}(\vec{r}\,') \right]. \tag{14}$$

This density means that an element of area on the boundary has the dipole moment of $\vec{D}_{\alpha,e} da$.

3. The third component is due to the discontinuity of the permittivity. In the undeformed geometry wherever that we have a dielectric interface (i.e. discontinuity of the ϵ_r) we have accumulated surface bound charges with the surface density $\sigma_b = (\vec{E}_0^{(2)} - \vec{E}_0^{(1)}) \cdot \vec{n}$. Using Eq. (12a), we find that displacement of these charges gives rise to a dipole density

$$\vec{D}_{\alpha,\epsilon}(\vec{r}) = \left[(\vec{E}_0^{(2)} - \vec{E}_0^{(1)}) \cdot \vec{n} \right] (\epsilon_r^{(2)} - \epsilon_r^{(1)}) \left[\vec{U}_{m,\alpha}(\vec{r}) - \vec{U}_{m,\alpha}(\vec{r}\,') \right]. \tag{15}$$

In summary, one can see $\vec{g}$ as an electric field with the corresponding electric displacement field $\epsilon_0 \epsilon_r \vec{g}$, in the presence a charge density $\delta\rho_e/\delta x_\alpha$ and a permanent polarization vector $\epsilon_0(\delta\epsilon_r/\delta x_\alpha)\vec{E}_0$ in addition to the boundary sources introduced in Equations 13, 14 and 15. In case of homogeneous materials and uniform charge densities, the bulk contributions vanish and we only have to deal with the boundary charges.

B. Green's function method for moving charges

In this section we introduce a formal method for calculating the mechanical-dielectric dynamical effects for systems which can be approximated as moving charges interacting with static dielectrics. We start by assuming the undeformed geometry to have an electrostatic Green's function, $\vec{G}(\vec{r}, \vec{r}\,', \omega)$, in a way that the electric field in the frequency domain is given by

$$\vec{E}(\vec{r}, \omega) = \frac{1}{4\pi\epsilon_0} \int d^3r' \rho_e(\vec{r}\,', \omega) \vec{G}(\vec{r}, \vec{r}\,', \omega). \tag{16}$$

This Green's function is in fact the gradient of the usual scalar Green's function used to compute the scalar potential and it is the solution of equations similar to Eq. (1), with a delta function charge density

$$\nabla \cdot \left[\epsilon_r(\vec{r}, \omega) \vec{G}(\vec{r}, \vec{r}\,', \omega) \right] = 4\pi\delta(\vec{r} - \vec{r}\,'), \tag{17a}$$

$$\nabla \times \vec{G}(\vec{r}, \vec{r}\,', \omega) = 0. \tag{17b}$$

The frequency dependence of $\vec{G}$ comes from the frequency dependence of ϵ_r. Now suppose that the body containing the charges goes under deformation with a displacement field $\vec{u}(\vec{r},\omega)$. When evaluating the electric field, this corresponds to modification of the source coordinates as $\vec{r}' \to \vec{r}' + \vec{u}(\vec{r}',\omega)$. For small oscillations (ie. $|\vec{u}(\vec{r}',\omega)| \ll |\vec{r}'|$), we can expand the electric field to the first order in $\vec{u}$. The higher order terms involve more complicated phenomena. For example the second order term results in frequency jitter [1]. We also assume that the charge density has no explicit frequency dependence. One obtains for the electric field

$$\vec{E}(\vec{r},\omega) = \frac{1}{4\pi\epsilon_0}\int d^3r' \rho_e(\vec{r}')\vec{G}(\vec{r},\vec{r}',\omega) + \frac{1}{4\pi\epsilon_0}\int_{V_r} d^3r' \nabla'\rho_e(\vec{r}') \cdot \vec{u}(\vec{r}',\omega)\vec{G}(\vec{r},\vec{r}',\omega) + \frac{1}{4\pi\epsilon_0}\int_{V_r} d^3r' \rho_e(\vec{r}')\nabla'\vec{G}(\vec{r},\vec{r}',\omega) \cdot \vec{u}(\vec{r}',\omega), \quad (18)$$

where ∇' corresponds to vector derivation with respect to the source coordinate $\vec{r}'$ and $\nabla'\vec{G}(\vec{r},\vec{r}',\omega)$ is a 3×3 tensor with elements $\partial G_i(\vec{r},\vec{r}',\omega)/\partial r'_j$. The first term Eq. (18) is the constant electrostatic field and has no dynamical effects. The second term corresponds to direct modification of the charge density due to its non-uniformity and the third term is a result of charge displacement. The integration for these two terms is over the volume of the resonator, V_r. For the third term, it can be shown that it is equivalent to the field generated by a polarization density field

$$\vec{P}_r(\vec{r},\omega) = \rho_e(\vec{r})\vec{u}(\vec{r},\omega). \quad (19)$$

For uniform charge distribution and simple geometries, this equivalence can be useful for more straightforward calculations. In the Methods section, we have used this equivalence to compute the NCF damping for an infinitesimally thin moving string.

C. Dissipated power and stored energy

Generally, the volume density of power dissipated in a dielectric is given by $p_d(\vec{r},t) = \vec{E}(\vec{r},t)\cdot\partial_t\vec{D}(\vec{r},t)$, where $\vec{D}(\vec{r},t)$ is the electric displacement field. If we plug the field variation from Eq. (8) into this relation, we would get different frequency mixing terms which is results in inter-modal damping. In a situation where the modes are sufficiently separated

in frequency compared to the damping, these effects are negligible and we can treat each mechanical mode separately with a single frequency varying electric field.

In dielectrics with dispersion and loss the relationship between $\vec{D}(\vec{r},t)$ and $\vec{E}(\vec{r},t)$ is not instantaneous and in the frequency domain is given by $\vec{D}(\vec{r},\omega) = \epsilon_0\epsilon_r(\vec{r},\omega)\vec{E}(\vec{r},\omega)$. Here $\epsilon_r(\vec{r},\omega)$ is the material's frequency dependent relative permitivity. For harmonic fields with the oscillation frequency ω, expressed by $\vec{E}(\vec{r},t) = \vec{E}(\vec{r},\omega)e^{-i\omega t} + \text{c.c.}$, the period averaged dissipated power density is given by

$$\bar{p}_d(\vec{r}) = 2\omega\epsilon_0\epsilon_r''(\vec{r},\omega)|\vec{E}(\vec{r},\omega)|^2, \tag{20}$$

where $\epsilon_r''(\vec{r},\omega) = \text{Im}[\epsilon_r(\vec{r},\omega)]$ characterises the material's dielectric loss. The dissipated power by the motion of a mode with displacements similar to Eq. (4) is given by

$$\bar{P}_d = \int d^3r 2\Omega_m\epsilon_0\epsilon_r''(\vec{r},\Omega_m)|\vec{g}(\vec{r},\vec{r};\vec{U}_m)|^2|x_m|^2. \tag{21}$$

Note that the field variation at each point is calculated in its own reference frame to account for the relative motion. For this mode, the motion can be reduced to *point-like* particle with the displacement $u(t) = x_m e^{-i\Omega_m t} + \text{c.c.}$. With a perturbative approach, when this particle is subjected to viscous damping force with the form $F_d = -\gamma_{\text{NCF}}\dot{u}_m(t)$, the power dissipated by this force, average for each period is given by

$$\bar{P}_d = 2\gamma_{\text{NCF}}\Omega_m^2|x_m|^2. \tag{22}$$

Comparing this equation with Eq. (25) yields the result of the main text.

$$\gamma_{\text{NCF}} = \int d^3r\, \epsilon_0|\vec{g}(\vec{r},\vec{r};\vec{U}_m(\vec{r}))|^2\frac{\epsilon_r''(\vec{r},\Omega_m)}{\Omega_m}. \tag{23}$$

On the other hand, the stored energy within the dielectrics, given by the density $\frac{1}{2}\vec{E}\cdot\vec{D}$, also gives rise to an energy term quadratic in the displacements of the mechanical resonator. With a similar approach as above for harmonic motion, the period average of this electrostatic energy is given by

$$\bar{E}_{\text{es}} = \int d^3r\epsilon_0\epsilon_r'(\vec{r},\Omega_m)|\vec{g}(\vec{r},\vec{r};\vec{U}_m)|^2|x_m|^2. \tag{24}$$

Referring this energy to an added spring constant $F_{\text{es}} = -k_{\text{es}}u_m(t)$ yields

$$k_{\text{es}} = \int d^3r\epsilon_0|\vec{g}(\vec{r},\vec{r};\vec{U}_m)|^2\epsilon_r'(\vec{r},\Omega_m). \tag{25}$$

D. Effect of finite conductivity

In addition to the dielectric loss due to ϵ_r'', a finite charge mobility and conduction within the dielectrics can also introduce dissipation. The dissipated power density for this mechanism is given by $p_d^{\mathrm{cond}}(\vec{r},t) = \vec{E}(\vec{r},t)\cdot\vec{J}(\vec{r},t)$, where $\vec{J}(\vec{r},t)$ is the current density. In frequency domain, the current density is given by the Ohm's law as $\vec{J}(\vec{r},\omega) = \sigma(\vec{r},\omega)\vec{E}(\vec{r},\omega)$, where $\sigma(\vec{r},\omega)$ is the material's conductivity that in general is a frequency dependent. Similar to the previous section, for a harmonic electric field with oscillation frequency ω, the period-averaged dissipated power density is given by

$$\bar{p}_d^{\mathrm{cond}}(\vec{r}) = 2\sigma(\vec{r},\omega)|\vec{E}(\vec{r},\omega)|^2. \tag{26}$$

For computing the ensuing NCF coefficient, we follow a similar procedure as in the previous section, yielding

$$\gamma_{\mathrm{NCF}}^{\mathrm{cond}} = \int d^3r\,|\vec{g}(\vec{r},\vec{r};\vec{U}_m(\vec{r}))|^2\frac{\sigma(\vec{r},\Omega_m)}{\Omega_m^2}. \tag{27}$$

For this result to be valid, we need to assume that at the frequency range of the motion the resonator the field variation introduced by the finite conductivity (i.e. correction to $\vec{g}$) is negligible. This means that the field variation due to the motion can be computed by solving the *zeroth order* electrostatic problem (i.e. no loss) and the dissipation is computed perturbatively to the first order of σ. Quantitatively, the validity condition reads

$$\sigma \gg \frac{\epsilon_0\epsilon_r}{\Omega_m}. \tag{28}$$

This condition is a direct outcome of the continuity equation $\nabla\cdot\vec{J} = -\partial_t\rho$, where ρ is the free carrier density. When combined with Gauss' law, one obtains an exponential decay for the carrier density with the time constant $\sigma/\epsilon_0\epsilon_r$. This time constant determines the speed at which the charges move to the boundaries to zero the field in the bulk. For $\sigma \ll \frac{\epsilon_0\epsilon_r}{\Omega_m}$, the charges on the resonator move so slowly that within the conductive materials around them the electric field is completely screened and any possible loss occurs on the boundaries. For $\sigma \gg \frac{\epsilon_0\epsilon_r}{\Omega_m}$, the motion is so fast that the charge carriers oscillate locally and do not modify the field within the material dramatically.

In summary, we can absorb both the dielectric loss and the conductivity into the loss tangent as

$$\tan\delta(\omega) = \frac{\epsilon_r''(\omega) + \frac{\sigma(\omega)}{\epsilon_0\omega}}{\epsilon_r'(\omega)}, \tag{29}$$

and rewrite the expression for the NCF coefficient as

$$\gamma_{\rm NCF} = \int d^3r\, \epsilon_0 \epsilon_r'(\vec{r},\Omega_m)|\vec{g}(\vec{r},\vec{r};\vec{U}_m(\vec{r}))|^2 \frac{\tan\delta(\Omega_m)}{\Omega_m}, \tag{30}$$

where the validity condition is simply $\tan\delta(\omega) \ll 1$. As seen in Eq. (27), having a finite electrical conductivity can modify the dependence of $\gamma_{\rm NCF}$ on the mechanical frequency to deviate from the $1/\Omega_m$ scaling. In particular, even for frequency independent permittivity and conductivity, for $\Omega_m < \sigma/\epsilon_0\epsilon_r''$ the scaling becomes $1/\Omega_m^2$.

E. Forces on the moving bodies

The dynamical effects discussed above can be found via computing the forces on the mechanical resonator. The perturbation due to the displacements give rise to forces with linear dependency on the position. The in-phase and out-of-phase components of these forces are interpreted as the modified spring constant and the damping coefficient. A general approach for computing the forces, that is consistent with the energy conservation, is via the electrostatic Maxwell's stress tensor, $\mathbf{T}$, defined as $T_{ij} = E_iE_j - \delta_{ij}|\vec{E}|^2$, where δ_{ij} is the Kronecker's delta. Applying the perturbation $\vec{E} = \vec{E}_0 + \vec{g}(\vec{r},\vec{r};\vec{U}_m(\vec{r}))u_m$ to $\mathbf{T}$ yields $\mathbf{T} = \mathbf{T}_0 + u_m(\delta\mathbf{T}/\delta u)$ where

$$\left(\frac{\delta\mathbf{T}}{\delta u}\right)_{ij} = \left(E_{0,i}g_j(\vec{r},\vec{r};\vec{U}_m(\vec{r})) + E_{0,j}g_i(\vec{r},\vec{r};\vec{U}_m(\vec{r})) - \delta_{ij}\vec{E}_0\cdot\vec{g}(\vec{r},\vec{r};\vec{U}_m(\vec{r}))\right). \tag{31}$$

Note that, similar to before, the field variation at each point is calculated in its own reference frame to account for the relative motion. The force unit volume applied on the resonator is given by $\nabla\cdot\mathbf{T}$ (note that this object is a vector) and the point-like force exerted on a certain mode, $\vec{U}_m$, is given by

$$F_u = \int_{V_r} d^3r \vec{U}_m \cdot (\nabla\cdot\mathbf{T}), \tag{32}$$

where the integration is over the volume of the resonator. Applying the perturbation field the position dependent force for the mode

$$F(u_m) = u_m \int_{V_r} d^3r \vec{U}_m \cdot \left(\nabla\cdot\frac{\delta\mathbf{T}}{\delta u}\right). \tag{33}$$

Referring this force to a spring constant and damping as $F(u_m) = -k_{\rm es}u_m - \gamma_{\rm NCF}\dot{u}_m$ gives

$$k_{\rm es} = -\int_{V_r} d^3r \,\mathrm{Re}\left[\vec{U}_m\cdot\left(\nabla\cdot\frac{\delta\mathbf{T}}{\delta u}\right)\right], \tag{34}$$

and

$$\gamma_{\rm NCF} = \frac{1}{\Omega_m}\int_{V_r} d^3r {\rm Im}\left[\vec{U}_m \cdot \left(\nabla \cdot \frac{\delta \mathbf{T}}{\delta u}\right)\right]. \tag{35}$$

Note the inverse frequency scaling appearing again. These forces take into account both the Coulomb forces on the charge as well as the dipole forces on the dielectrics. For situations where we can neglect the dipole forces, there is a major simplification and the force density on the resonator is given by $\rho_e \vec{g}(\vec{r}, \vec{r}; \vec{U}_m(\vec{r}))$. The computation in the reference frame of the resonator ensures that the *self-terms* do not appear and there will be no singularities. With this simplification the dynamical effects are given by

$$k_{\rm es} = -\int_{V_r} d^3r \rho_e {\rm Re}\left[\vec{U}_m \cdot \vec{g}(\vec{r}, \vec{r}; \vec{U}_m(\vec{r}))\right], \tag{36}$$

and

$$\gamma_{\rm NCF} = \frac{1}{\Omega_m}\int_{V_r} d^3r \rho_e {\rm Im}\left[\vec{U}_m \cdot \vec{g}(\vec{r}, \vec{r}; \vec{U}_m(\vec{r}))\right]. \tag{37}$$

These expressions are particularly useful for point-like charges where computing the force is significantly simpler than computing the dissipated power. In the AFM community, this approach is more common as the charge on the cantilever is modeled by a point charge [1].

II. NONCONTACT FRICTION FOR AN INFINITESIMALLY THIN STRING

In this section we reproduce the results for the problem a thin string oscillating in front of semi-infinite dielectric slab, as shown in Fig. 2(a) using two methods, other than the one discussed in the Methods section. We also compute NCF for the situation where the dielectric slab is finite and is placed on a conductor.

A. Semi-infinite dielectric slab: explicit approach

We compute the NCF damping coefficient by the explicit method of taking the derivative of the stationary field with respect to the amplitude of motion. The schematic is shown in Fig. 2(b). To find the field in $z > 0$, using the technique of image charges we can simply replace the charge density of the beam with $(1 + \xi)\rho_{e,l}$ (with $\xi = \frac{\epsilon_r - 1}{\epsilon_r + 1}$). When $d \ll L$, the static field is approximately given by the field from an infinitely long line of charge.

$$\vec{E}_0(\vec{r}; d) = \frac{(1 + \xi)\rho_{e,l}}{2\pi\epsilon_0\epsilon_r} \frac{y\hat{y} + (z + d)\hat{z}}{y^2 + (z + d)^2} \tag{38}$$

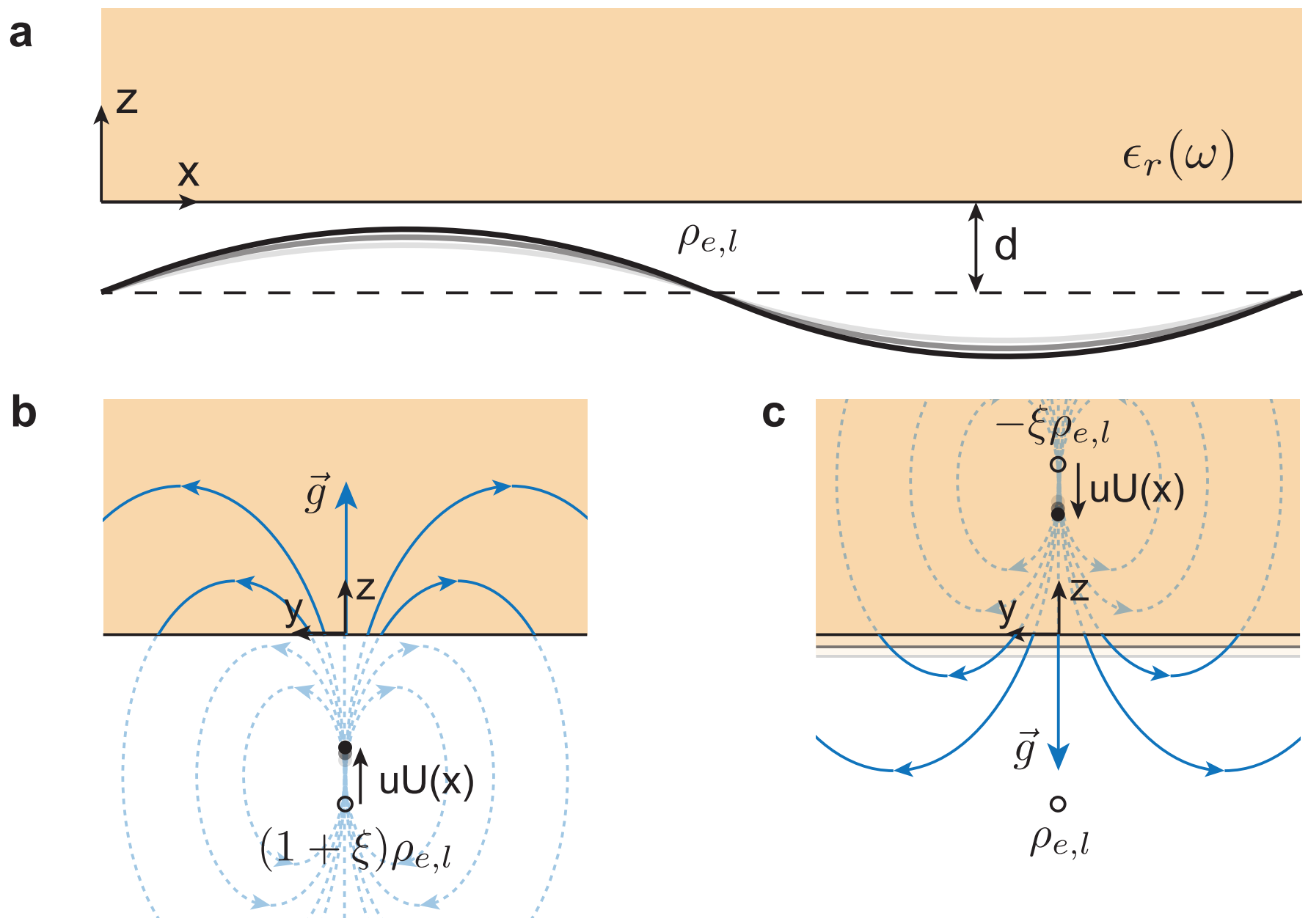

FIG. 2. **Noncontact friction for an infinitesimally thin string a** The string is along the x-axis and it oscillates along the z-axis. It located in air, with distance d from a semi-infinite dielectric slab with complex relative permittivity $\epsilon_r(\omega)$. **b** View of **a** in the yz plane, showing the motion of the string and the electric field variation (blue streamlines).

To calculate the field variation $\vec{g}(\vec{r})$ under the deformations given by a mode shape $U(x)$ (motion along the z-axis), in the frame of reference of the dielectric slab, we consider a displacement with amplitude q for the line of charge. For modes where $d \ll |U'(x)|^{-1}$ we can approximately reduce the problem to 2D in the cross-section. As illustrated in Fig. 2(b), we calculate the field in coordinate x from a line of charge that is displaced by $uU(x)$. The field variation is given by

$$\vec{g}(\vec{r}; U(x), \omega) = \frac{\partial}{\partial u}\left[\vec{E}_0(\vec{r}; d - uU(x))\right]\Big|_{u=0} = \frac{(1+\xi)\rho_{e,l}}{2\pi\epsilon_0\epsilon_r}\frac{2y(z+d)\hat{y} + \left[(z+d)^2 - y^2\right]\hat{z}}{\left[y^2 + (z+d)^2\right]^2}U(x). \quad (39)$$

Which is the same result as derived in the Methods section.

$$Q_{\mathrm{NCF},n} = \frac{8\pi\epsilon_0\rho_{m,l}d^2}{\rho_{e,l}^2}\frac{\Omega_n^2}{\mathrm{Im}[\xi(\Omega_n)]}, \quad (40)$$

B. Semi-infinite dielectric slab: out-of-phase force

To compute the damping coefficient using the out-of-phase force, we have to compute $\vec{g}$ in the frame of reference of the string. As shown in Fig. 2(c), in this frame the string is stationary and the dielectric slab oscillates. As discussed in sectionn I, the source for $\vec{g}$ in this situation is the moving bound surface density given by the discontinuity of $\vec{E}_0$ at $z = 0$. But according to the image charges technique, the field made by these charges in $z < 0$ is equivalent to the one generated by an image charge with density $\xi\rho_{e,l}$ at $z = d$. Hence, similar as the previous section, for displacement of $uU(x)$, the electric field variation in $z < 0$ is given by

$$\vec{g}(\vec{r}; U(x), \omega) = \frac{\partial}{\partial u}\left[\frac{-\xi\rho_{e,l}}{2\pi\epsilon_0}\frac{y\hat{y} + (z - d + uU(x))\hat{z}}{y^2 + (z - d + uU(x))^2}\right]\Bigg|_{u=0} = \frac{\xi\rho_{e,l}}{2\pi\epsilon_0}\frac{2y(z-d)\hat{y} + \left[(z-d)^2 - y^2\right]\hat{z}}{\left[y^2 + (z-d)^2\right]^2}U(x). \quad (41)$$

Using Eq. (37), the NCF damping coefficient is given by

$$\gamma_{\rm NCF} = \frac{1}{\Omega_n}\int_0^L dx\rho_{e,l}{\rm Im}\left[U(x)\hat{z}\cdot\vec{g}(x, 0, -d)\right], \quad (42)$$

which yields

$$\gamma_{\rm NCF} = \frac{\rho_{e,l}^2}{2\pi\epsilon_0(2d)^2}\frac{{\rm Im}\left[\xi\right]}{\Omega_n}\int_0^L |U(x)|^2 dx. \quad (43)$$

Using the definition of the effective mass $m_{\rm eff} = \rho_{m,l}\int_0^L |U(x)|^2 dx$ we obtain the same result as before.

C. A finite dielectric slab on a conductor

The schematic of this problem is shown in Fig. 3. A dielectric layer with the complex permittivity ϵ_r and thickness Δ is located on top of a conductor and the string has a distance d from the dielectric surface. To solve this problem, we first find the appropriate image charges configurations for a point charge Q at $z = d$. These configurations for finding the electric field in $-\Delta < z < 0$ and $z > \Delta$ are shown in Fig. 3(a) and Fig. 3(b) respectively. One can easily show that the boundary conditions at $z = 0$ and $z = -\Delta$ are satisfied with

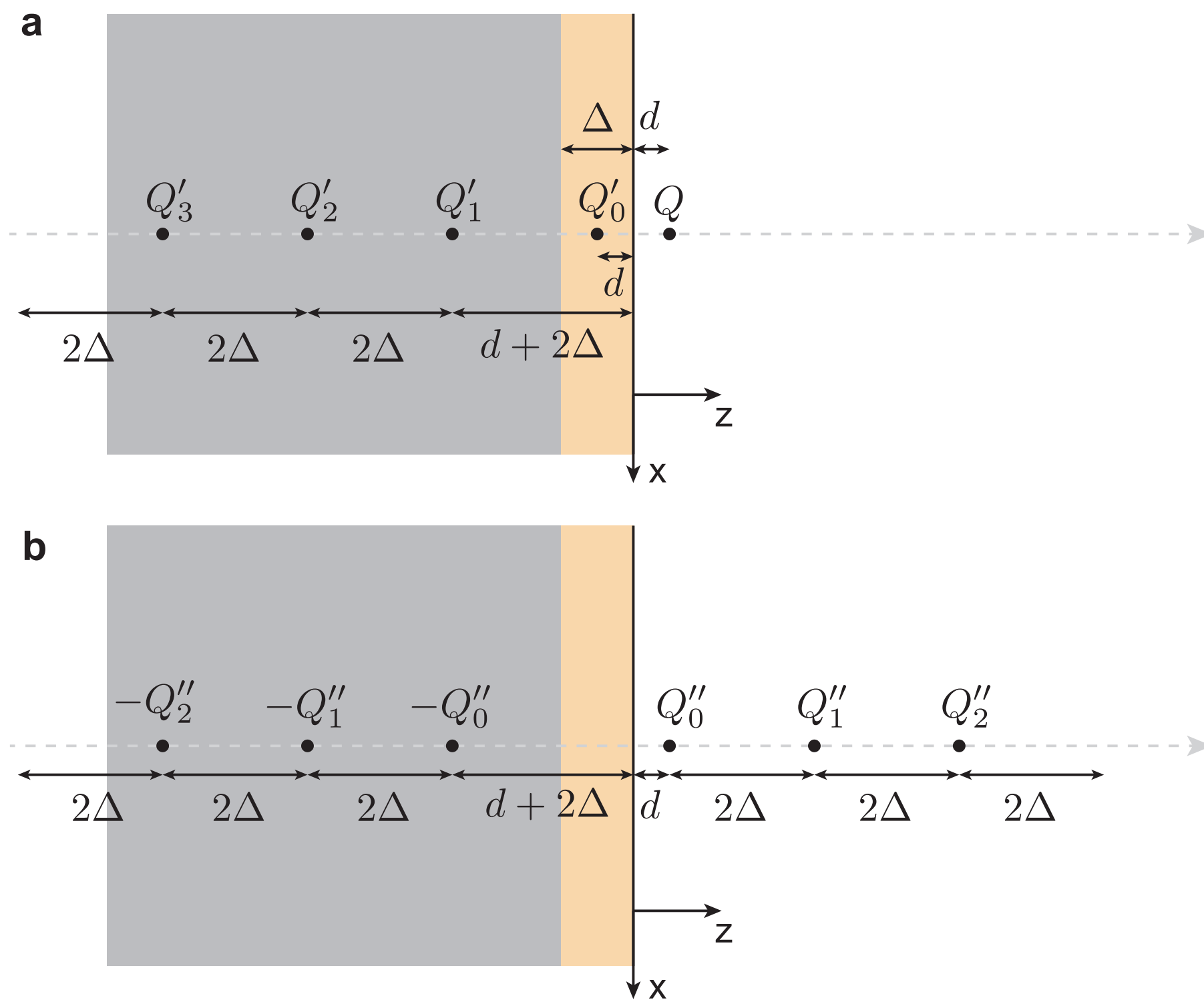

FIG. 3. **Image charges for a finite dielectric slab on a conductor a** The image charges for the solution in $z > 0$. **b** Image charges for the solution in $-\Delta < z < 0/$.

the following values for the image charges. With $Q'_i = \alpha_i Q$ and $Q''_i = \beta_i Q$ we have

$$\alpha_0 = -\xi, \tag{44}$$

$$\alpha_i = \frac{(1-\xi^2)}{\xi}(-\xi)^i \quad (i = 1, 2, ...), \tag{45}$$

$$\beta_i = (1+\xi)(-\xi)^i, \quad (i = 0, 1, ...), \tag{46}$$

where $\xi = \frac{\epsilon_r - 1}{\epsilon_r + 1}$. To find the NCF damping coefficient we use the force calculation method as it is much easier. Similar to the previous section, to find the force on the string at $z = d$ due to the displacements, we place the lines dipole densities with dipole moments $\vec{P}_l^i = \alpha_i \rho_{e,l} U(x) \hat{z}$ at the locations of Q'_i in Fig. 3(a). A calculation similar to the previous section yields

$$\gamma_{\mathrm{NCF}} = \frac{m_{\mathrm{eff}} \rho_{e,l}^2}{8\pi\epsilon_0 \rho_{m,l} d^2} \mathrm{Im} \left[\xi + \frac{1-\xi^2}{\xi} \sum_{i=1}^{\infty} \frac{(-\xi)^i}{\left(1 + i\frac{\Delta}{d}\right)^2} \right]. \tag{47}$$

Similar to the approach in [3], we evaluate the infinite summation by replacing the denominators using the identity $b^{-2} = \int_0^\infty dv v e^{-bv}$

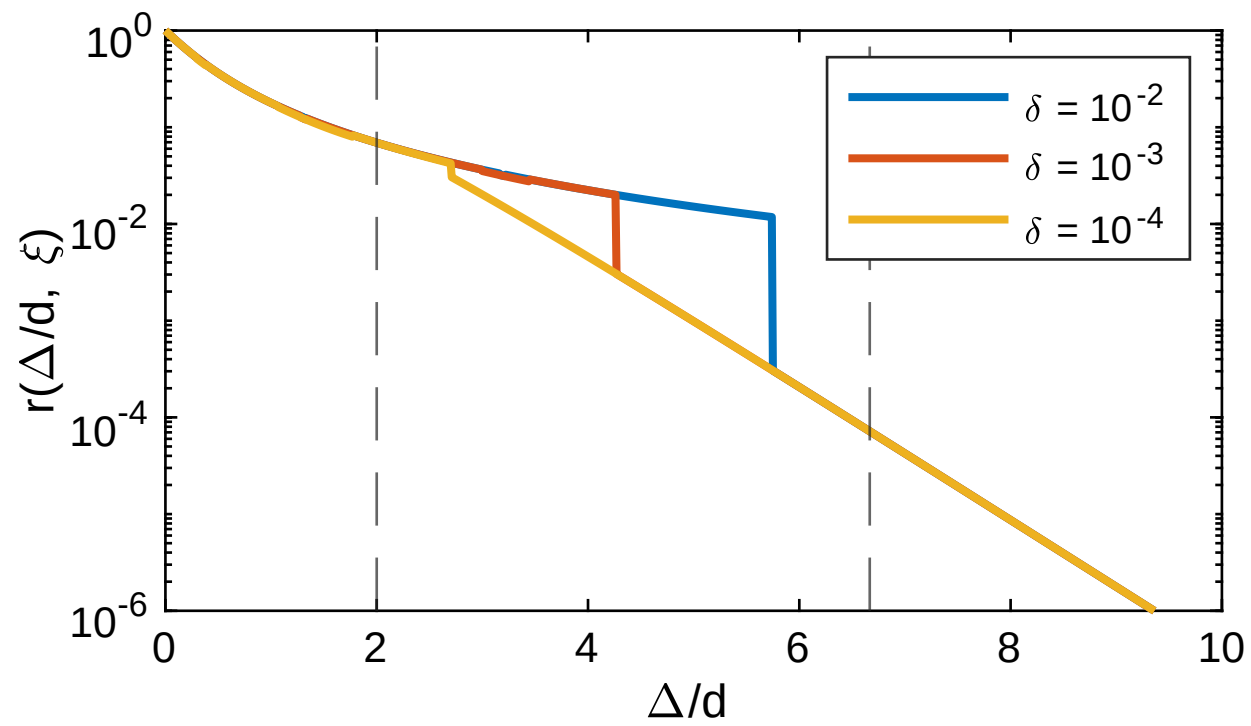

FIG. 4. **Correction to the NCF damping coefficient due to the conductive substrate.** The correction factor, $r(\frac{\Delta}{d}, \xi)$, defined in Eq. (49) as a function of Δ/d. Values are computed for SiO_2 with the permittivity $\epsilon_r = 3.9(1 + \tan\delta)$, for three different values of δ.

$$\gamma_{\mathrm{NCF}} = \frac{m_{\mathrm{eff}}\rho_{e,l}^2}{8\pi\epsilon_0\rho_{m,l}d^2}\frac{\mathrm{Im}\,[\xi]}{\Omega_n}\left(1 - r(\frac{\Delta}{d}, \xi)\right), \tag{48}$$

where

$$r(\frac{\Delta}{d}, \xi) = \frac{\mathrm{Im}\left[(\xi^2 - 1)\int_0^\infty dv \frac{ve^{-(1+\frac{\Delta}{d})v}}{1+\xi e^{\frac{\Delta}{d}v}}\right]}{\mathrm{Im}\,[\xi]}. \tag{49}$$

For SiO_2 with $\epsilon_r = 3.9(1+\tan\delta)$, r is shown in Fig. 4 as a function of Δ/d, for different values of the loss tangent. As one can see, the correction due to the presence of the conductive substrate is smaller for thicker oxide layers and the model converges to the semi-infinite dielectric substrate. For the thicknesses used in our experiments this correction is less than 10%.

D. NCF damping in ultracoherent resonators above Si substrates

In Fig. 1(c) of the main text we have shown the measured and simulated quality factors for three resonators from Ref. [4–6] and we also shown the estimated the NCF-limited Q for these resonators, induced by a thin layer of native oxide on the surface of the Si substrate. For the ease of comparison with other friction measurements, we have shown the corresponding damping coefficients (i.e. $\gamma = m_{\mathrm{eff}}\Gamma_m$) and spring constants (i.e. $k_{\mathrm{eff}} = m_{\mathrm{eff}}\Omega_m^2$) in Fig. 5.

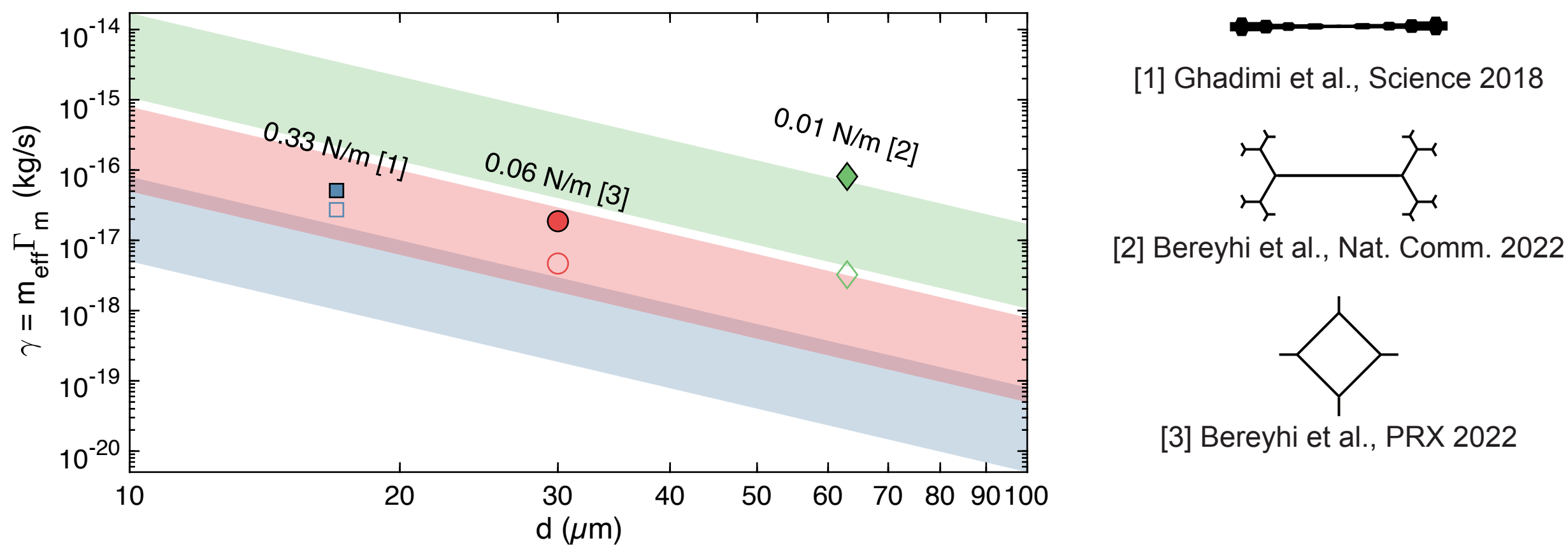

FIG. 5. **NCF in ultracoherent nanomechanical resonators above substrates** NCF damping induced by the substrate, limiting the quality factor of ultracoherent strings from Ref. [4–6]. The colors blue, red and green correspond to strained-engineered, hierarchical and polygon designs, shown in the right hand side. The ribbons correspond to the estimated NCF-induced damping coefficient for the spring constant (noted on the plot) and geometry of each design, suspended above a silicon substrate with a 4 nm native SiO_2 layer. The filled and empty markers correspond to the measured and simulated damping coefficient adapted from the references.

III. GENERAL 3D TO 2D REDUCTION

In this we introduce the mathematical details of the procedure for reducing a problem with translational symmetry from 3D to 2D. This procedure is useful both in analytical solutions and FEM simulations. Consider a geometry where $\vec{E}_0$ does not depend on one of the coordinates (say, z). Moreover, the mode shape also varies slowly with z and can be written as $\vec{U}(x,y,z) = \vec{U}_\perp(x,y)U_\parallel(z)$. We define the mode cross section as

$$S_\perp = \int dxdy|\vec{U}_\perp(x,y)|^2. \tag{50}$$

One can easily show that the effective mass of the mode is given by $m_{\text{eff}} = \rho_m S_\perp \int dz U_\parallel(z)^2$. For such a geometry, the field variation $\vec{g}$ can be approximated as

$$\vec{g}(x,y,z) = \vec{g}_\perp(x,y;\vec{U}_\perp(x,y))U_\parallel(z). \tag{51}$$

Here $\vec{g}_\perp$ is the transverse counterpart of $\vec{g}$. With these definitions, the NCF damping coefficient can be written as

$$\gamma_{\text{NCF}} = \frac{\epsilon_0}{\Omega_m}\int dxdy\epsilon_r''(x,y)|\vec{g}_\perp(x,y)|^2\int dz U_\parallel(z)^2, \tag{52}$$

which allows us to reduce the computations for problems with this symmetry. For the case of the uniform beams above dielectric surfaces, since the length of the dielectric surface coincides with the length of the resonator we can further simplify

$$\gamma_{\rm NCF} = \frac{\epsilon_0 m_{\rm eff}}{\Omega_m \rho_{m,l}} \int dxdy \epsilon_r''(x,y) |\vec{g}_\perp(x,y)|^2, \tag{53}$$

where $\rho_{m,l} = \rho_m S_\perp$. These relations are used for the NCF calculations based on the FEM simulation of the cross section of the devices.

IV. FINITE ELEMENT MODEL FOR COMPUTATION OF NCF COEFFICIENT

Our method is based on the definition of the field variation noted in the main text

$$\vec{g} = \frac{\partial \vec{E}_0}{\partial x}, \tag{54}$$

where $\vec{E}_0$ is the electric for the undeformed geometry and x is the amplitude of motion of for a particular mechanical mode. The finite element simulation computes the electric field for the two cases of undisplaced and marginally displaced geometry and then calculates the field variation using a numerical derivative. The steps of the FEM simulation are summarized in the following way:

1. The mechanical mode shape is simulated (without any charges or electrostatic interaction).

2. A charge distribution is defined on the resonator.

3. The electric field is simulated at two positions of the mechanical resonator: first with no displacement and second displaced by the amplitude Δx on the mode shape of interest.

4. The field variation, $\vec{g}$ is computed numerically based on the two simulations $\vec{g} = \frac{\vec{E}(\Delta x) - \vec{E}(\Delta x = 0)}{\Delta x}$.

5. NCF damping is computed using the volume integral formula

$$\gamma_{\rm NCF} = \frac{\epsilon_0}{\Omega_m} \int d^3 r \epsilon_r'' |\vec{g}|^2 \tag{55}$$

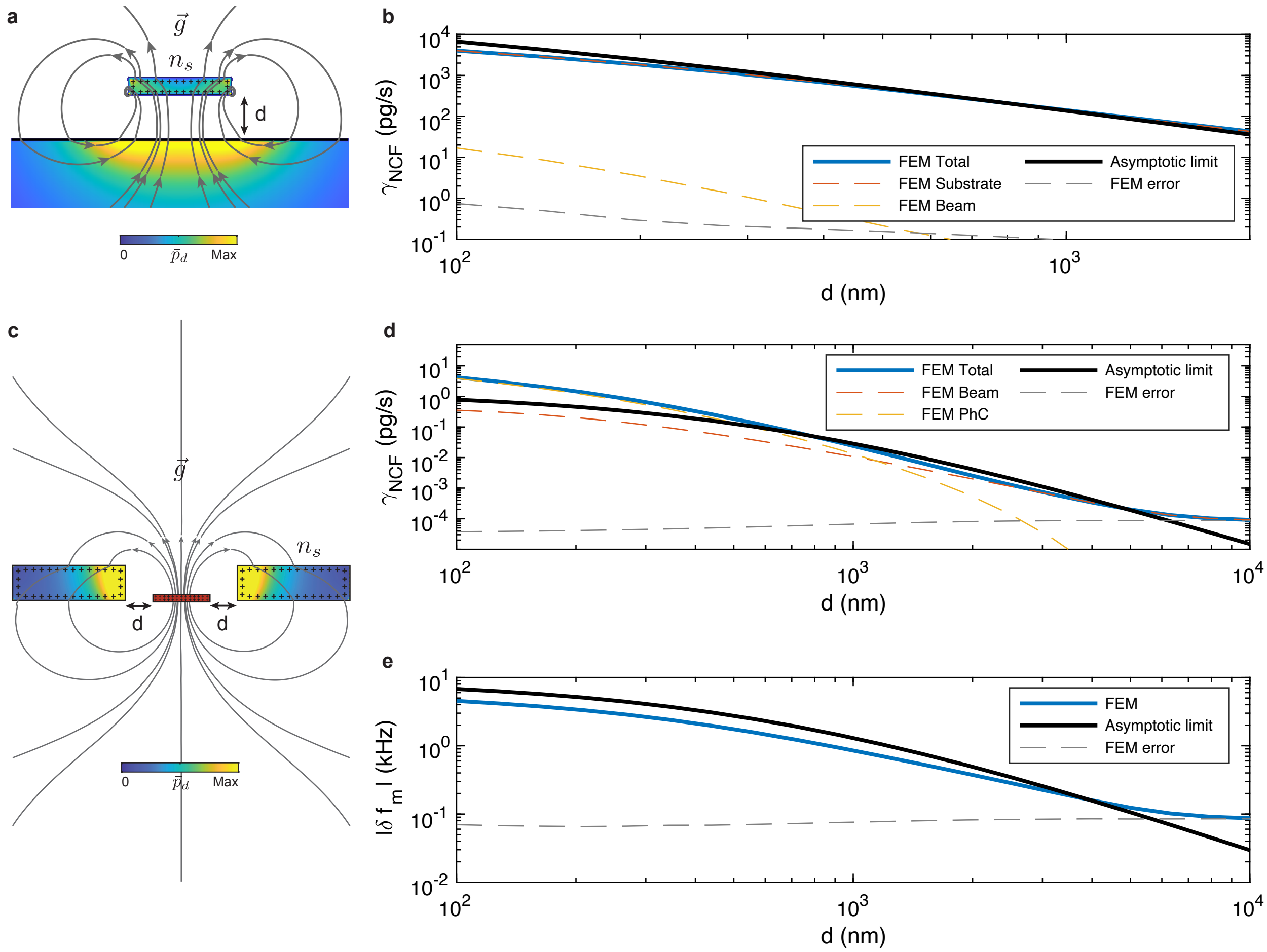

FIG. 6. **Asymptotic behavior of the FEM models a** FEM simulation of the rectangular string above a SiO_2 substrate. All the surfaces of the string are charged with a uniform density. The simulation volume is bounded by sufficiently far zero-potential boundaries (not shown). **b** NCF damping coefficient and its constituent components vs. gap size, obtained from the simulation in **a** using Eq. (60). Dashed gray line: the numerical error of the FEM simulation, equal to the value obtained for $\gamma_{\mathrm{NCF}}^{\mathrm{beam}}$ when the dielectric substrate is removed. **c** FEM simulation of the nano-optomechanical system. All surfaces are charged with a uniform charge density. **d** Same components as **b**, computed for the simulation in **c**. **e** Electrostatic frequency of the string obtained for the simulation in **c** and using Eq. (**??**).

To account for the relative motion and to calculate the loss within the moving bodies, the simulation has to be done on a mesh that deforms alongside the geometry. We use the *moving mesh* feature in COMSOL Multiphysics.

In most physical systems, the geometry is composed of discrete bodies, each with a

constant dielectric constant. In such systems we can rewrite Eq. (55) as

$$\gamma_{\mathrm{NCF}} = \frac{1}{\Omega_m} \sum_i 2 \tan \delta_i W_i \tag{56}$$

where

$$W_i = \frac{1}{2} \epsilon_0 \epsilon_{r,i} \int_{V_i} d^3 r |\vec{g}|^2, \tag{57}$$

is the electrostatic energy (generated by $\vec{g}$) within the body with index i, volume V_i, relative permittivity $\epsilon_{r,i}$ and dielectric loss tangent of $\tan \delta_i$.

Both physical systems that we have discussed in this paper, to a good approximation, have the 1D translational symmetry. The numerical calculations can be done in the 2D cross section of the systems and using the method of Appendix III NCF damping coefficient can be written as

$$\gamma_{\mathrm{NCF}} = \frac{\int_{L_{\mathrm{int}}} dz U_{\parallel}(z)^2}{\Omega_m} \sum_i 2 \tan \delta_i W_{\perp,i}, \tag{58}$$

where

$$W_{\perp,i} = \frac{1}{2} \epsilon_0 \epsilon_{r,i} \int_{S_i} d^2 r |\vec{g}_{\perp}|^2, \tag{59}$$

is the integration of $g_{\perp}$ over the cross section of the i-th body and L_{int} is the length along which the mechanical mode interacts with the dielectrics.

A. Uniform beams above a dielectric surface

As discussed in Appendix III, for this system $L_{\mathrm{int}} = L_{\mathrm{beam}}$ and γ_{NCF} is given by the sum of two components: dissipation within the SiO_2 substrate and the dissipation within the Si_3N_4 beam itself.

$$\gamma_{\mathrm{NCF}} = \gamma_{\mathrm{NCF}}^{\mathrm{beam}} + \gamma_{\mathrm{NCF}}^{\mathrm{substrate}}, \tag{60}$$

where

$$\gamma_{\mathrm{NCF}}^{\mathrm{i}} = \frac{m_{\mathrm{eff}}}{\rho_{m,l} \Omega_m} 2 W_i \tan \delta_i. \tag{61}$$

As shown in Fig. 6(a), we simulate a Si_3N_4 100×400 nm beam, uniformly charged on its surface, suspended above a SiO_2 substrate. We show γ_{NCF}, as well as its two components for different gaps in Fig. 6(b). To benchmark this simulation, we compare its asymptotic behavior for large gaps, to an infinitesimally thin beam with equal linear charge density. We also show the analytical value for the thin beam in Fig. 6(b) and observe good convergence.

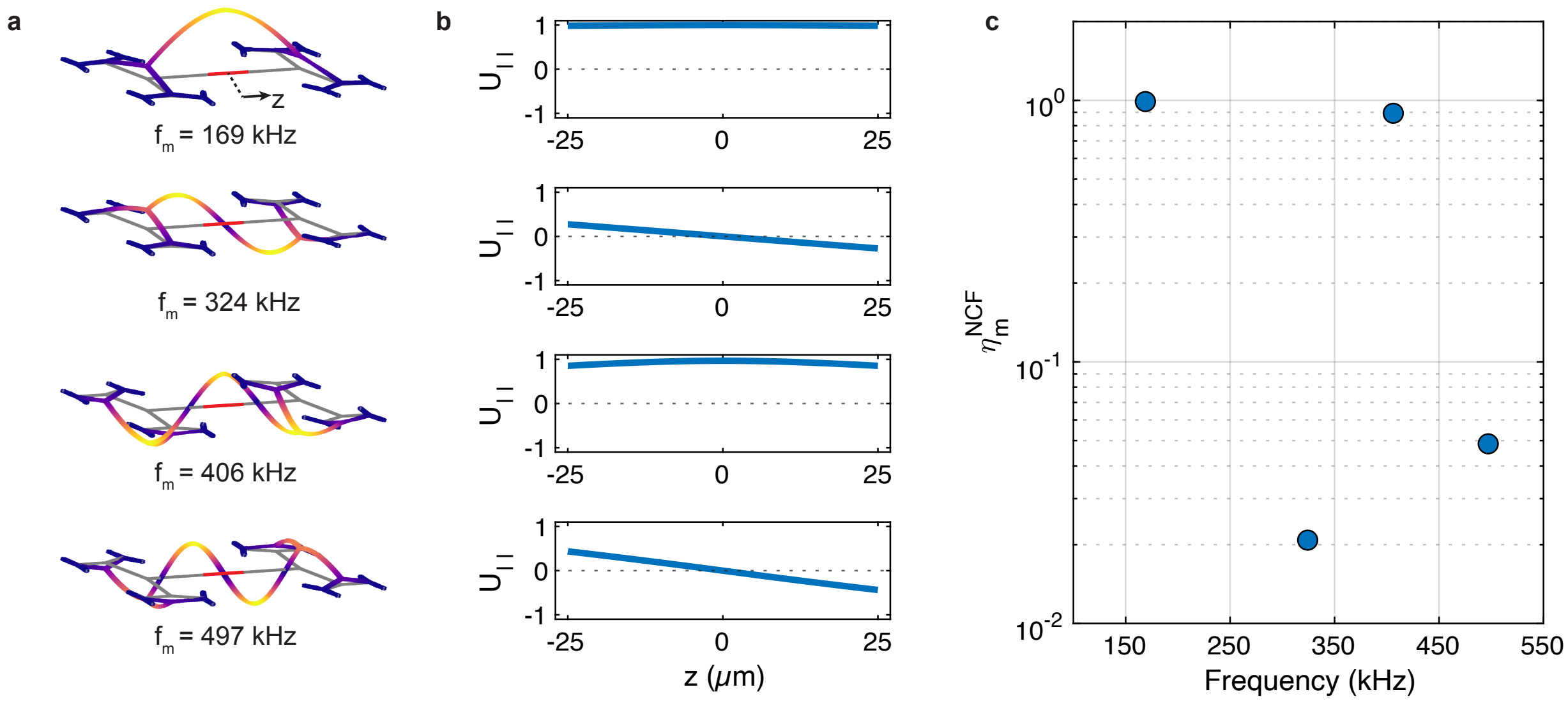

FIG. 7. **Contribution of different modes of the binary-tree resonators to NCF a** FEM simulations of the mode shapes of the first 4 OP modes of the resonator used in the main text to study the dependence on the mode shape. **b** Mode shape along the red line marked in **a** for different modes. **c** Mode contribution to NCF, η_m^{NCF}, computed from the mode shapes in **b** using Eq. (64).

We note that the convergence can be improved by enlarging the simulation region, which is resource-consuming for large gaps.

In the main text in Section II, to obtain the surface charge densities we have the effective linear density obtained from the fits. The relation between this effective density and the surface density for uniform distribution is obtained by comparing Eq. (60) and the analytical result of the thin string which yields,

$$\rho_{e,s}^2 = \frac{{\rho_{e,l}^{\mathrm{eff}}}^2 \epsilon'_{r,\mathrm{sub}}}{8\pi\epsilon_0 d^2(1+\epsilon'_{r,\mathrm{sub}})^2} \frac{\tan\delta_{\mathrm{sub}}}{\tan\delta_{\mathrm{sub}} W_{\mathrm{sub}} + \tan\delta_{\mathrm{beam}} W_{\mathrm{beam}}}, \tag{62}$$

where in the FEM simulations for computing W_i we have assumed $\rho_{e,s} = 1$.

B. Integrated nano-optomechanical system

For this system, neglecting the holes of the photonic crystals, the interaction length equals the length of the PhC, $L_{\mathrm{int}} = L_{\mathrm{PhC}}$. To use the approximations of Appendix III, we need two conditions: (i) to have $\vec{E}_0$ (i.e. the electric field at the equilibrium position of the resonator)

to be z-independent and (ii) to have the mechanical mode to be separable to transverse and longitudinal components. Since the gap in these devices (d <600 nm) is much smaller than the interaction length ($L_{\mathbf{int}} = L_{\mathrm{PhC}}$ ∼50 µm) the first condition is met within the interaction region. In the binary-tree resonators used in this paper, the flexural mechanical modes within the interaction length are nearly one-dimensional functions. Hence the second condition is also met and we can reduce the problem to 2D and write the NCF damping coefficient as

$$\gamma_{\mathrm{NCF}} = \frac{\eta_m^{\mathrm{int}}}{\Omega_m} L_{\mathrm{cav}} \sum_i 2 \tan \delta_i W_{\perp,i}, \tag{63}$$

where

$$\eta_m^{\mathrm{int}} = \frac{1}{L_{\mathrm{cav}}} \int_{L_{\mathrm{cav}}} dz |U_{\parallel}(z)|^2 \tag{64}$$

is the contribution of the mechanical mode (indexed with m) to the NCF dissipation within the cavity. η_m^{int} is computed from the mechanical mode simulation and is between 0 and 1. An example of computation of η_m^{int} is shown in Fig. 7. Similarly to the uniform beams, we can write γ_{NCF} for this system as

$$\gamma_{\mathrm{NCF}} = \gamma_{\mathrm{NCF}}^{\mathrm{beam}} + \gamma_{\mathrm{NCF}}^{\mathrm{PhC}}, \tag{65}$$

corresponding to the dissipation within the beam and the PhC cavities. These components are computed from our FEM model shown in Fig. 6(c). Similar to the main text, we have assumed uniform charge distribution on all the surfaces. We show the total NCF damping coefficient and its constituent components in Fig. 6(b). To compute the asymptotic limit at far distances, we model the PhC cavities as cylinders exposed the uniform electric generated by the beam where the field within the dielectric cylinder acquires a factor $\frac{2}{1+\epsilon_r^{-1}}$. The deviation of the FEM value from the asymptotic limit is due to the finite size of the simulation space which leads to an error shown in the same figure. This deviation can be reduced by augmenting the simulation space.

Our FEM simulation can be used to compute the frequency shift in the integrated samples induced by the electrostatic force. When the volume of the resonator is negligible, we can compute the spring constant using by computing the force exerted on the charges by the field variation. In this case, for a small displacement of the mode $\vec{U}_m$ with the amplitude x, the resonator is exposed to a force volume density of $\vec{f}_r = \rho_e \vec{g} x$. The point-like equivalent of this force for this mode is given by $F_r = \int d^3 r \vec{U}_m \cdot \vec{f}_r$ which gives $F_r = x \int d^3 r \rho_e \vec{U}_m \cdot \vec{g}$.

Now we can define the electrostatic-induced spring constant as

$$k_{\mathrm{es}} = \int d^3r \rho_e \vec{U}_m \cdot \vec{g}. \tag{66}$$

When reducing to 2D, we obtain

$$k_{\mathrm{es}} = -\int_{L_{\mathrm{cav}}} dz |U_{\parallel}(z)|^2 \int_{S_r} d^2r \rho_e \vec{U}_{\perp} \cdot \vec{g}, \tag{67}$$

where we have neglected the force exerted on the resonator outside the interaction region. The mode shape in 2D is only a unit vector $\vec{e}_{\perp}$. Using the simulated $\vec{g}_{\perp}$ with constant surface density $\rho_{e,s}$ on the resonator, we can compute the frequency shiftconstant

$$\delta\Omega_m = -\rho_{e,s} \frac{\eta_m^{\mathrm{int}}}{2m_{\mathrm{eff}}\Omega_m^2} L_{\mathrm{cav}} \int_{\partial S_i} dr \vec{e}_{\perp} \cdot \vec{g}_{\perp}. \tag{68}$$

This quantity is shown in Fig. 6(e). The asymptotic limit for this case is when the PhCs and string are so far that they can be approximated as lines of charge for which we have an analytical result

$$\delta\Omega_m = -\frac{\eta_m^{\mathrm{int}}}{2m_{\mathrm{eff}}\Omega_m} \frac{\rho_{e,l}^{\mathrm{beam}} \rho_{e,l}^{\mathrm{PhC}}}{\pi \epsilon_0 d^2}, \tag{69}$$

where $\rho_{e,l}^i$ is the charge per unit length for the string and PhCs. This result is also shown in Fig. 6(e), showing a good agreement with the FEM result. The agreement can be improved by augmenting the simulation volume.

V. FABRICATION PROCESSES

A. Uniform strings on oxide samples

The process flow for fabrication of these devices is shown in Fig. 8(a). An initial layer of *wet* oxide is grown on the Si $< 100 >$ substrate through wet thermal oxidation, serving both as a dielectric body for the NCF measurements and as an etch-stop layer during fabrication. A sacrificial polySi layer ($\approx$ 600 nm) and a high-stress ($\approx$ 1.1 GPa) stoichiometric Si_3N_4 device layer ($\approx$ 200 nm) are then deposited via low-pressure chemical vapor deposition (LPCVD). The devices are patterned using optical lithography with a maskless aligner, followed by dry etching of both the device and the sacrificial layers using fluorine chemistry (SF_6). This plasma etching process likely induces charging in the devices, leading to the noncontact friction effects described earlier. The well-controlled deposition rate of polySi

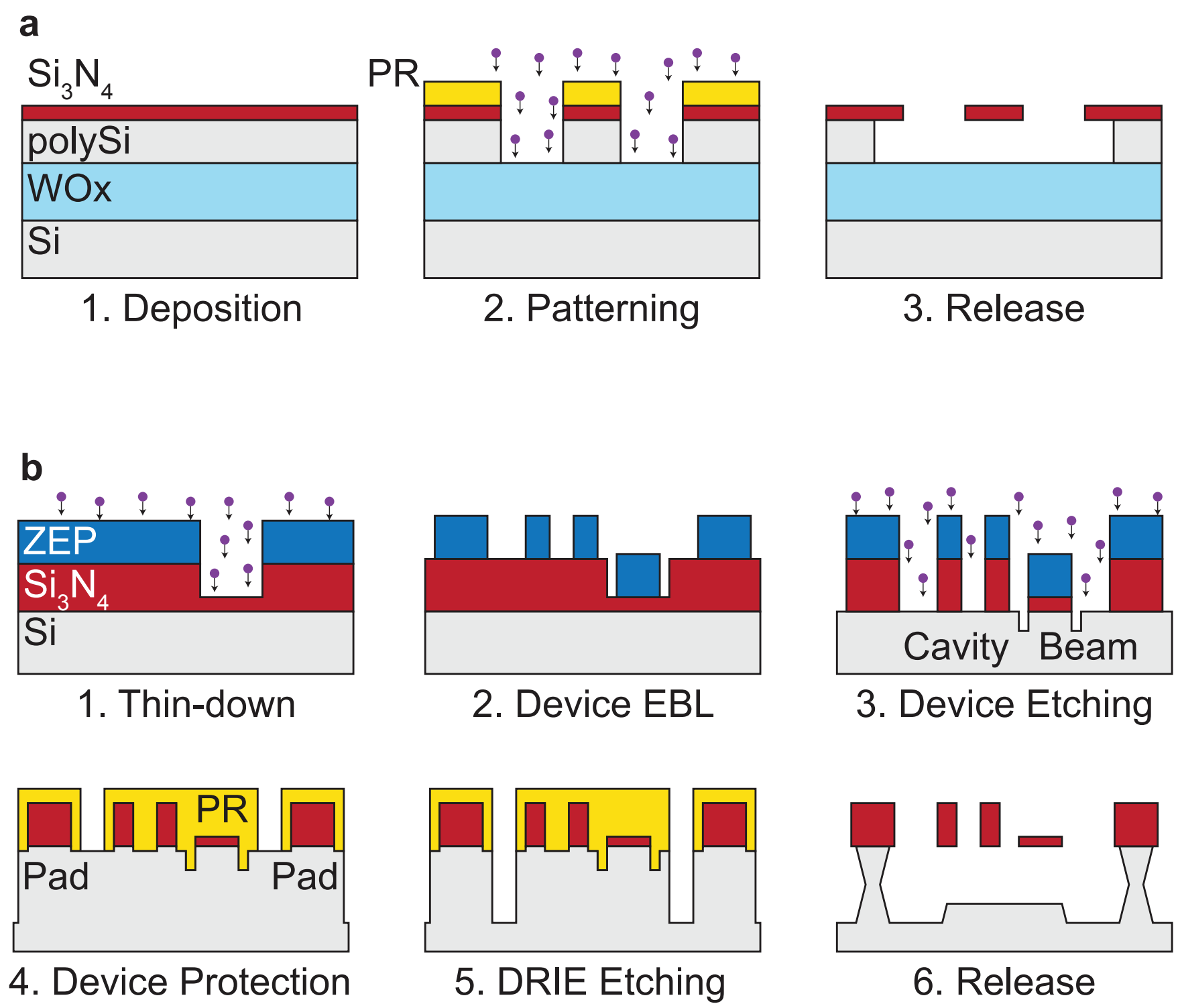

FIG. 8. **Fabrication process flows.** **a** Uniform strings on oxide samples. The initial stack consists of *wet* SiO_2 (WOx, 4μm), polySi (600 nm), and stoichiometric high-stress Si_3N_4 (200 nm). Nanobeams are patterned in the Si_3N_4 using optical photolithography and suspended through an initial vertical dry etching and finally release through isotropic etching with XeF_2. **b** Integrated nano-optomechanical devices. A 250 nm-thick stoichiometric Si_3N_4 layer is locally thinned down to below 40 nm in selected areas using e-beam lithography (EBL) and ZEP as the resist followed by dry etching. Nanobeams are then patterned in the thinned regions, while photonic crystal (PhC) cavities and supporting pads are defined in the thicker areas, also via EBL and dry etching. The structures are subsequently protected with photoresist (PR) and released and suspended using a combination of dry (DRIE etching) and wet etching.

LPCVD, combined with the high selectivity ($\approx$ 20:1) of the etching process, ensures precise control of the gap size—a key factor in the findings presented in this paper. Finally, the wafers are diced, and chip-scale release is performed using XeF_2 isotropic etching. For these patterns, the XeF_2 selectivity for polySi over Si_3N_4 is approximately 330:1, largely depending on the exposed polySi area. The etching of polySi generates radical byproducts in the form of SiF_x, which in turn attack Si_3N_4 [7]. Nonetheless, the selectivity remains sufficient to

suspend the structures with reasonable reproducibility. The survival yield of these devices strongly depends on their length and width, reaching unity for beams wider than 2 μm and increasing as length decreases for narrower beams. The lower yield observed in narrower beams is attributed to their reduced stiffness, making them more susceptible to collapse due to electrical charging effects [8].

B. Integrated nano-optomechanical devices

The process flow for fabrication of these devices is shown in Fig. 8(b). The fabrication process begins on a Si $< 100 >$ substrate with a 250 nm-thick high-stress stoichiometric Si_3N_4 layer deposited via low-pressure chemical vapor deposition (LPCVD), sourced from Hahn-Schickard-Gesellschaft. Nanobeam channels are first patterned and locally thinned to below 40 nm using e-beam lithography and fluorine-based dry etching. A second e-beam lithography step defines the nanobeams, photonic crystal cavities, and supporting pads for the clamping points, which are then etched using fluorine plasma. Both plasma processes can contribute to electrical charging of the Si_3N_4 structures. Following patterning, the devices are protected with photoresist, and deep reactive ion etching (DRIE) is performed to facilitate release. At this stage, the wafers are diced, and chip-scale release is achieved through potassium hydroxide (KOH) etching, hydrochloric acid (HCl) neutralization, and critical-point drying (CPD). A selectivity on the order of $10^5 : 1$ is required to successfully release the 40 nm-thick nanobeams. To investigate the possible causes of eventual surface charges on the samples, devices have sometimes been exposed to a 1 minute-long buffered oxide etch right before the CPD step. This is the case, for example, of Chip 3 in Fig. **??**(e). The survival yield primarily depends on the gap size, i.e., the distance between the PhC cavity and the nanobeam. For gaps exceeding 600 nm, the survival yield is above 80%, whereas for gaps of 200 nm or smaller, it drops below 20%.

VI. SUPPLEMENTARY DATA FOR THE WIDTH DEPENDENCE

Throughout the main text, we assumed uniform charge surface densities on all the surfaces of the strings. While this assumption is sufficient to obtain average/order of magnitude values, there is no reason that the charge distribution, at least over the cross section of the

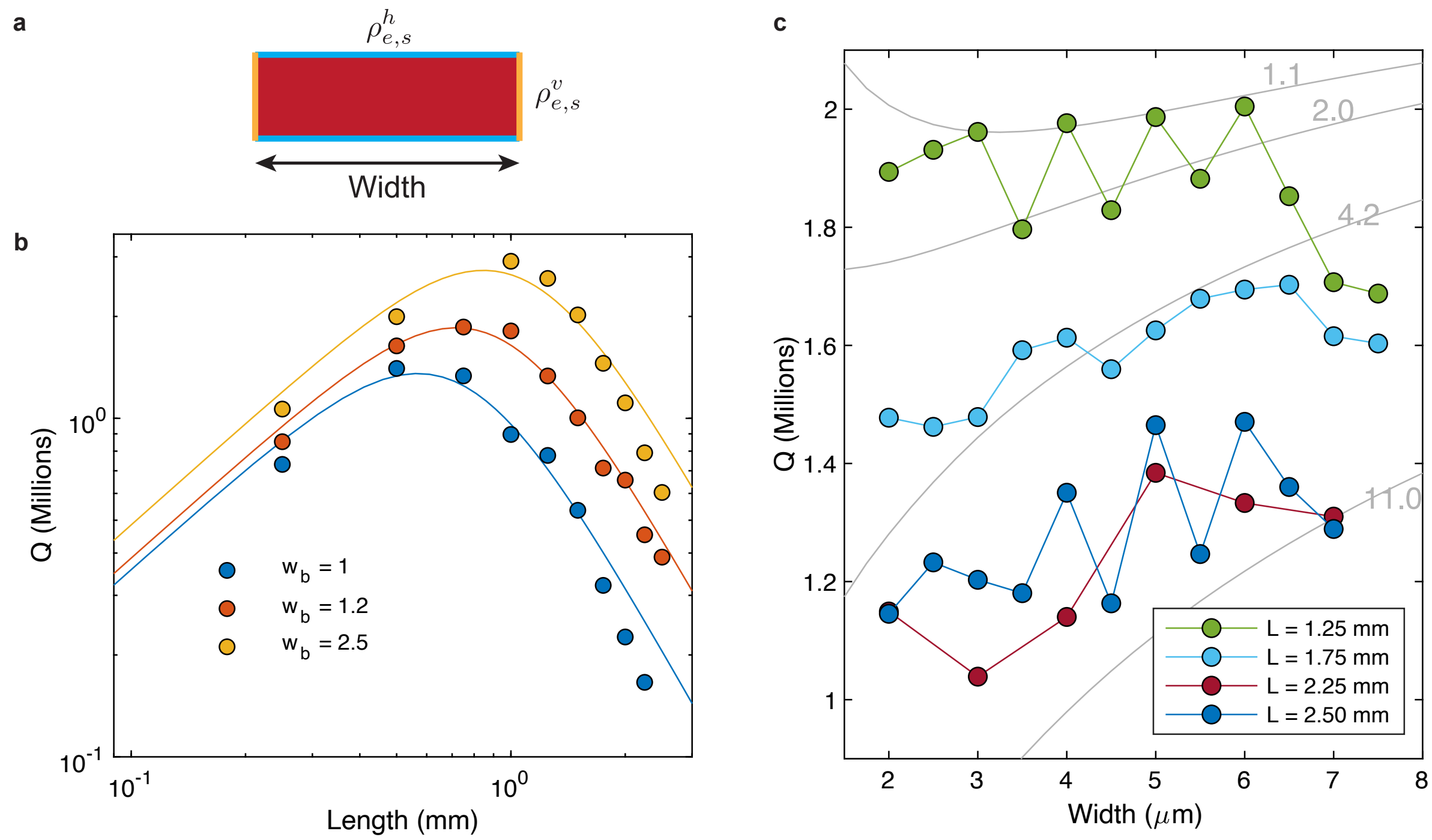

FIG. 9. **Dependence of the Q of the uniform strings above a substrate on their width** **a** Cross section of a string showing different surface charge densities on the horizontal (blue) and vertical (yellow) surfaces. **b** Q of the fundamental mode as a function of the string lengths for three different widths. The solid lines correspond to fits using the model used in the main text. **c** Q of the fundamental mode as a function of the string widths for three different lengths. The gray solid lines correspond to the FEM simulations only for visual comparison. The number written on each line is the vertical to horizontal distributions ratio, $\rho^v_{e,s}/\rho^h_{e,s}$.

devices, is uniform. For example. As illustrated in Fig. 9(a), the charge distribution on the horizontal and vertical surfaces of the strings ($\rho^h_{e,s}$ and $\rho^v_{e,s}$ respectively) can be different. This can be due to the fact that the vertical surfaces have been subjected to plasma etching (used for patterning the devices) which can introduce surface defects. The charge density difference manifests itself as the dependence of NCF on the width of the strings. Fig. 9(b) shows a measurement of the Q for fundamental modes of strings above the SiO_2 substrate with different lengths, for three families of devices with different widths. The increasing Q for wider strings can be explained by the toy model of section II and the horizontal/vertical density difference. If the vertical density is much larger than the horizontal one ($\rho^v_{e,s} \gg \rho^h_{e,s}$), wider beams effectively have the same linear charge density but they have a higher mass,

resulting in higher Q.

To study in more detail, we fabricate devices with fixed lengths and varying widths in the 2-7 µm range and measure the Q for their fundamental modes. The results for 4 length families are shown in Fig. 9(c). As expected, the longer strings have lower Qs, confirming the significant contribution of NCF in these devices. We also observe an overall increasing dependence on the width. Since the widths of these devices becomes bigger than the string-substrate gap (i.e. 500 nm) we cannot use the toy model and use the FEM simulations to study the width dependence. We also show these results in Fig. 9(c), for different values of $\rho^v_{e,s}/\rho^h_{e,s}$ ratios. We observe that for $\rho^v_{e,s}/\rho^h_{e,s} > 2$, the dependence of Q on width becomes monotonically increasing. The experimental data and the FEM results are superimposed in Fig. 9(c) for visual comparison. Although the data quality and the agreement with the model are not very consistent, the qualitative agreement for the trends suggests that the charge density on the vertical sidewalls is higher, which could be explained by the surface damage from plasma etching.

VII. SIGNATURES OF SLOW CHARGE DYNAMICS

In amorphous materials and particularly glass (i.e. SiO_2), slow dynamics of trapped charges and tunneling events can give rise to a non-vanishing charge mobility. Such dynamics have been observed in both SiO_2 and Si_3N_4 in microelectromechanical systems (MEMS) [9] and also in glass vacuum chambers used for experiments with Rydberg atoms [10]. Here we discuss two observations that can be potentially explained by charge fluctuations.

A. Slow drifts of the NCF-limited damping in strings

Throughout our experiments, for devices with NCF-limited mechanical Q, we have observed slow drifts of the measured Qs. From the devices presented in section 3 of the main text, we pick one of the devices whose Q is dominated by NCF. This device is from Chip 1 and its fundamental mode has the frequency of 100 kHz. Via repeated ringdown measurements, we measure the damping rate of this string for several hours in minute intervals. Fig. 10(a) shows the outcome of these measurements, showing variations up to 30% over time. In Fig. 10(b) we show the power spectral density of the variations of the measured

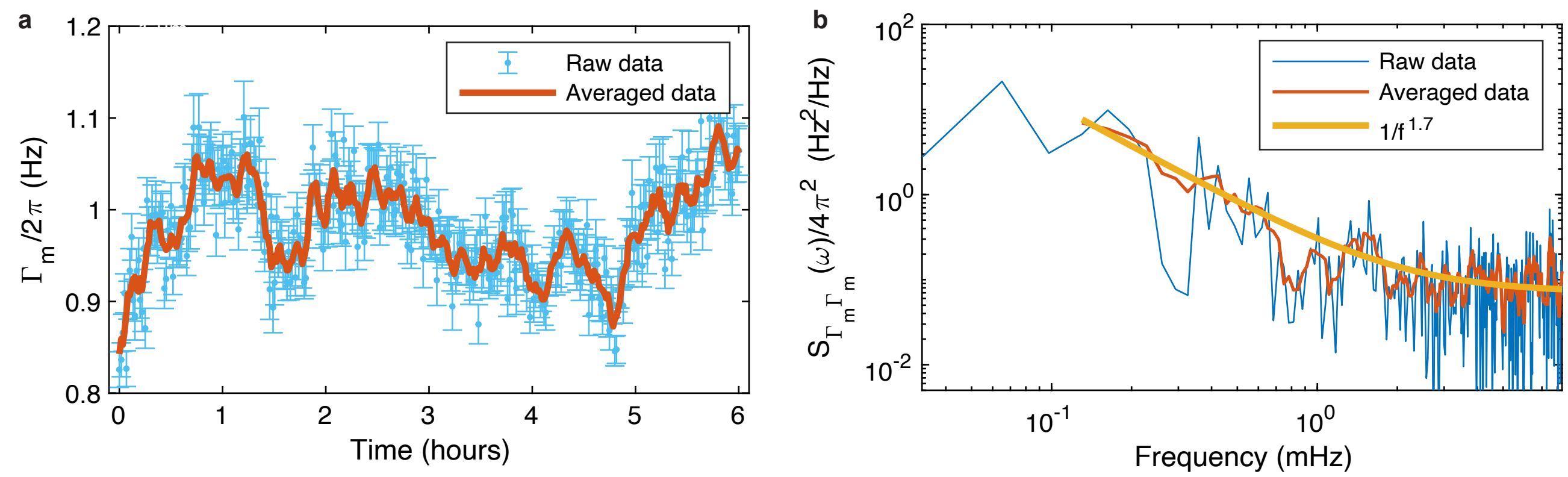

FIG. 10. **Slow temporal variations of NCF. a** Measurement record of damping rate of the fundamental mode of a 2.5 mm string from chip 1 of Fig. 2 of the main text over 6 hours in 1 minute intervals. Blue markers: result of an exponential fit to each ringdown measurement with error bar corresponding to the fitting 95% confidence interval. Orange line: A moving average over the data. **b** Power spectral density (PSD) of the measured damping rate in **a**. Blue line: PSD of the raw data. Orange line: PSD of the moving average. Yellow line: A power law fit to the averaged data.

damping rates. At low frequencies, we fit a power law scaling of $S_{\Gamma_m\Gamma_m}(\omega) \propto \omega^{-1.7}$. Similar to the flicker noise in electronics, these power law variations are typically a signature of trapping dynamics (for example see chapter 8 of Ref. [11]). To conclude that the observed fluctuations are due to charge fluctuations and the interaction with the substrate, the data here has to be compared with Γ_m fluctuations of a fully released (i.e. no earby substrate) string of similar dimensions. As we do not have such a device we cannot make this concrete comparison. However, since the damping rate of this device is dominated by NCF by a large margine (the NCF-limited Q is about 2 orders of magnitude smaller than the bending-limited one), we can argue that the observed fluctuations are due to fluctuations in NCF, which is most likely due to charge dynamics.

B. Mechanical frequency fluctuations

Another signature of the slow dynamics is observed in the mechanical frequency fluctuations of the binary-tree strings in the nano-optomechanical devices. Using an established technique [12], we measure the frequency fluctuations of the mechanical modes of these

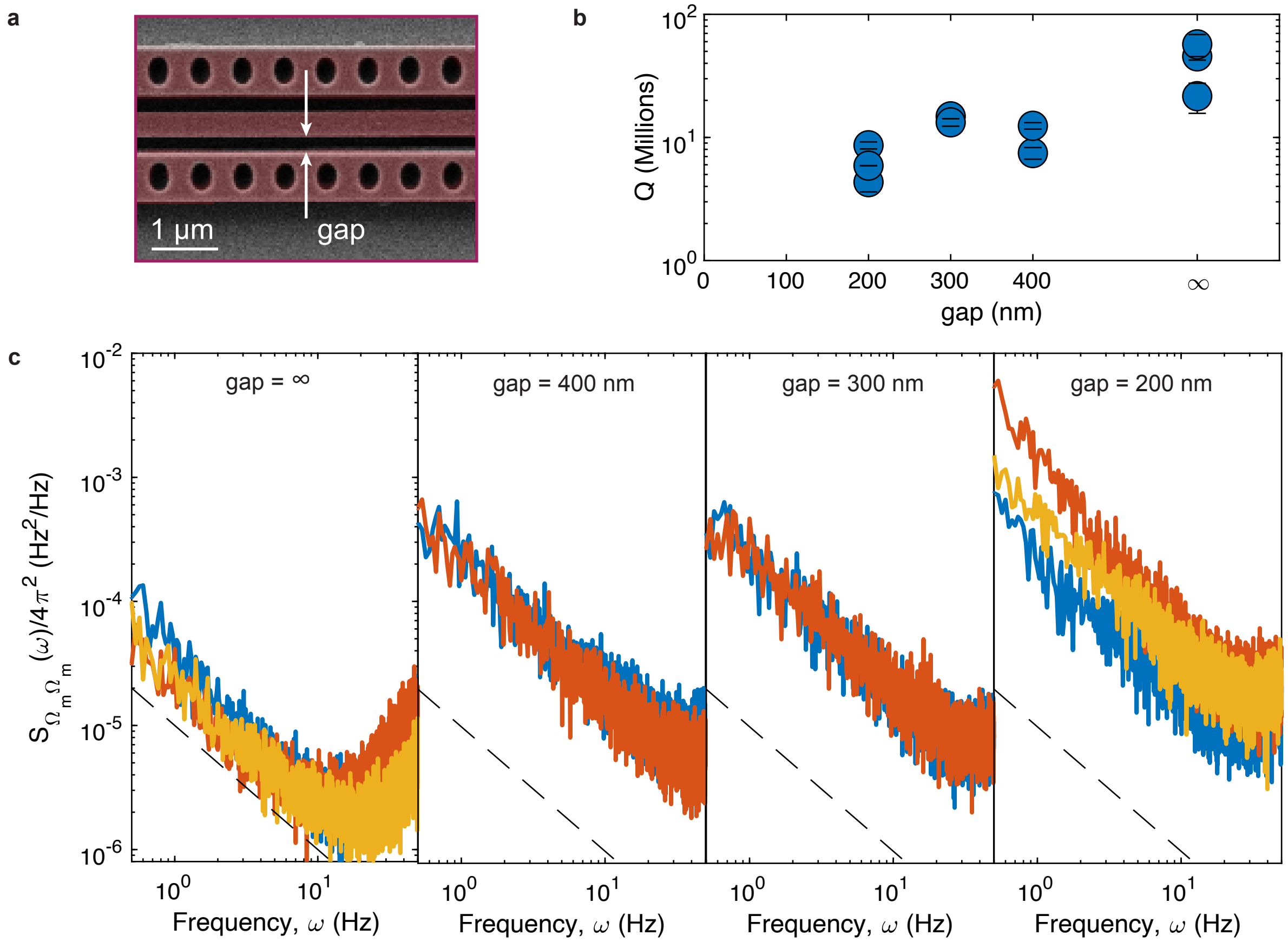

FIG. 11. **Gap dependent mechanical frequency noise.** **a** false colored SEM of the integrated device (same as Fig.3(b) of the main text). **b** Measured Q of reference and integrated binary-tree devices. **c** Power spectral density of the mechanical frequency fluctuations of the 10 devices in **b**. Dashed line: a guide to the eye for visualizing the $1/\omega$ scaling.

devices using our free-space interferometer. In this technique, the mechanical mode is continuously and resonantly driven by a piezoelectric actuator, driven by a local electronic oscillator. The displacement measurement signal obtained from the interferometer is electronically demodulated using the same local oscillator, which allows phase detection at the frequency of the mechanical mode. The detected phase is fed to a phase-locked loop which phase-locks the local oscillator to the mechanical mode that eventually yields a real-time record of the frequency fluctuations of the mechanical mode. This procedure is implemented using the built-in functionalities of a Zurich Instruments lock-in amplifier. We have studied the mechanical frequency fluctuations using 3 reference (gap $= \infty$) and multiple integrated devices with gaps of 200, 300 and 400 nm). Measurement of the quality factors of the fun-

damental modes of these devices at $\Omega_m = 180\,\text{kHz}$ (shown in Fig. 11(b)) reveals that the integrated devices are indeed affected by NCF. In Fig. 11(c) we show the power spectral density of the measured frequency fluctuations for these 6 devices. The frequency noise, $S_{\Omega_m\Omega_m}(\omega)$ at frequencies above 20 Hz (i.e. $\omega/2\pi > 20\,\text{Hz}$), is dominated by imprecision (detection) noise in the displacement measurement. For lower frequencies the noise corresponds to the frequency fluctuations of the mode. For the reference devices (gap $= \infty$), we observe that all the 3 devices closely follow a flicker-type $1/\omega$ noise with the same magnitudes. This flicker frequency noise has been repeatedly observed in nanomechanical resonators (for example see [13] and [14] for Si_3N_4 resonators) and is typically referred to as the intrinsic frequency noise and is explained by the reorientation dynamics of defects in the solid body of the resonator [15]. Interestingly, for the integrated devices, we observe at least 10 times higher level of the $1/\omega$ noise. We believe that these excess fluctuations are due to the interaction of the mechanical resonator with the defects or charge traps within the Si_3N_4 photonic crystal cavities. For other ensembles of devices, although we have not observed a clear scaling with the gap size, we have consistently observed that the integrated devices exhibit a higher level of the $1/\omega$ noise.

VIII. GEOMETRY PARAMETERS FOR THE BINARY-TREE RESONATORS

Fig. 12 shows the schematic of a segment of a binary-tree resonator alongside the quantities that parametrize its geometry. These parameters are described in Table I. These parameters in addition to the Si_3N_4 film parameters (thickness and stress) are used to simulate the mechanical mode using COMSOL and compute the properties (Frequency, Q and effective mass) of the first few mechanical modes. We also computes the NCF interaction factor given by Eq. (64) (See Fig. 7). The geometry parameters for different devices used in the experiments are shown in Table II

IX. OVERVIEW OF FORCE SENSITIVITY AND DISSIPATION OF NANOMECHANICAL RESONATORS.

In Fig. 13, we compare the sensitivity of selected nanomechanical resonances. In Fig. 13(a) thermal limited force sensitivity, $S_F^{\text{th}} = 4k_B T m_{\text{eff}}\Gamma$ is shown versus the resonance fre-

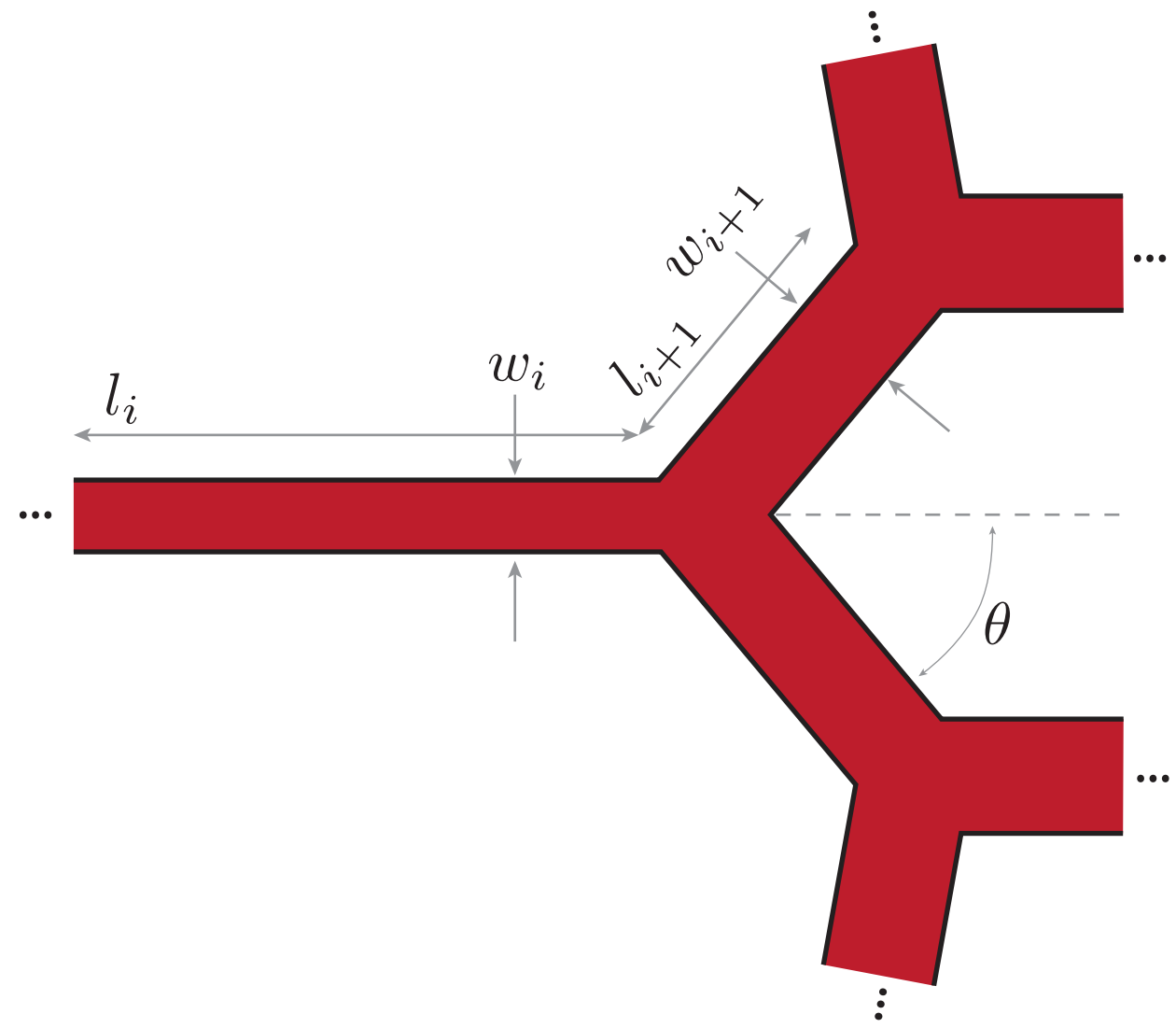

FIG. 12. **Geometry parametrization of the binary-tree resonators** The illustration shows a branching point in the resonator. Parameters are described in Table I. For more details see Ref [16].

Symbol	Description
l_0	Length of the central segment
r_l	Length expansion ratio l_{i+1}/l_i
w_0	Width of the central branch
r_w	Width expansion ratio w_{i+1}/w_i
θ	Branching angle
N	Number of branchin generations
w_p	Width of the interaction region

TABLE I. Geometry parameters of the binary-tree resonators

quency, Ω_m, and in Fig. 13(b) for the same resonators we compare the dissipation coefficient, $\gamma = m_{\text{eff}}\Gamma_m$, versus the effective spring constant $k_{\text{eff}} = m_{\text{eff}}\Omega_m^2$.

The resonators grouped as "Functionalized Si_3N_4" correspond to Si_3N_4 high-Q resonators that are functionalized either with optical microcavities, nano-magnets or micro-pillars and magnetic tips. The "EPFL" group corresponds to the binary-tree resonator used in this work and the polygon resontor from Ref. [6] which is state of the art of ultracoherent Si_3N_4 nanomechanics at room temperature in terms of force sensitivity. The projection to 100 mK

Experiment	Run #	Chip #	l_0 (µm)	r_l	w_0 (nm)	r_w	θ	N	w_p (nm)
Thermal force	1	1	250	0.59	210	1.1	82	2	420
Gap sweep	1	1	250	0.59	210	1.1	82	2	430
Mode shape dependence	2	1	250	0.60	210	1.27	79	3	430
Frequency dependence	3	1	250	0.6	210	1.3	83	2	430
Frequency dependence	3	2	200	0.65	210	1.18	79	3	430
Frequency dependence	4	3	150	0.63	210	1.2	81	2	430

TABLE II. Parameters table of different resonators used in the experiments

in this group correponds to only changing T in $S_F^{\mathrm{th}} = 4k_B T m_{\mathrm{eff}} \Gamma$ from 300 K to 100 mK and no improvement in the quality factor is considered. Two cryogenic AFM cantilevers, a silicon nanowire and a carbon nanotube resonator are also included for comparison.

X. ATTEMPTS FOR MITIGATION OF NCF

A. In situ thermal annealing

It has been experimentally shown that thermal annealing of microfabricated devices can modify the charge density on them [17]. We have done thermal annealing on the nano-optomechanical devices under vacuum. First, the samples are annealed at 80 degrees for about 1 hour under vacuum and the quality factors are remeasured. As shown in Fig. 14, while still far from the reference values, we observe a significant improvement in the Qs. We have performed a second annealing experiment on the same chip at 120 degrees for about 1 hour, upon which we observe reduction of the Qs even below the pre-bake values. We cannot draw a strong conclusion from these experiments and more systematic experiments are required to understand the impact of thermal annealing on the charge densities.

B. Ultraviolet annealing

It has also been shown that UV irradiation can modify the charge density in Si_3N_4 [18, 19]. We have tested this method also on a nano-optomechanical device. We have used a 250 nm UV LED (thorlabs LED250J) with above 1 mW power. We illuminated the device at ambient

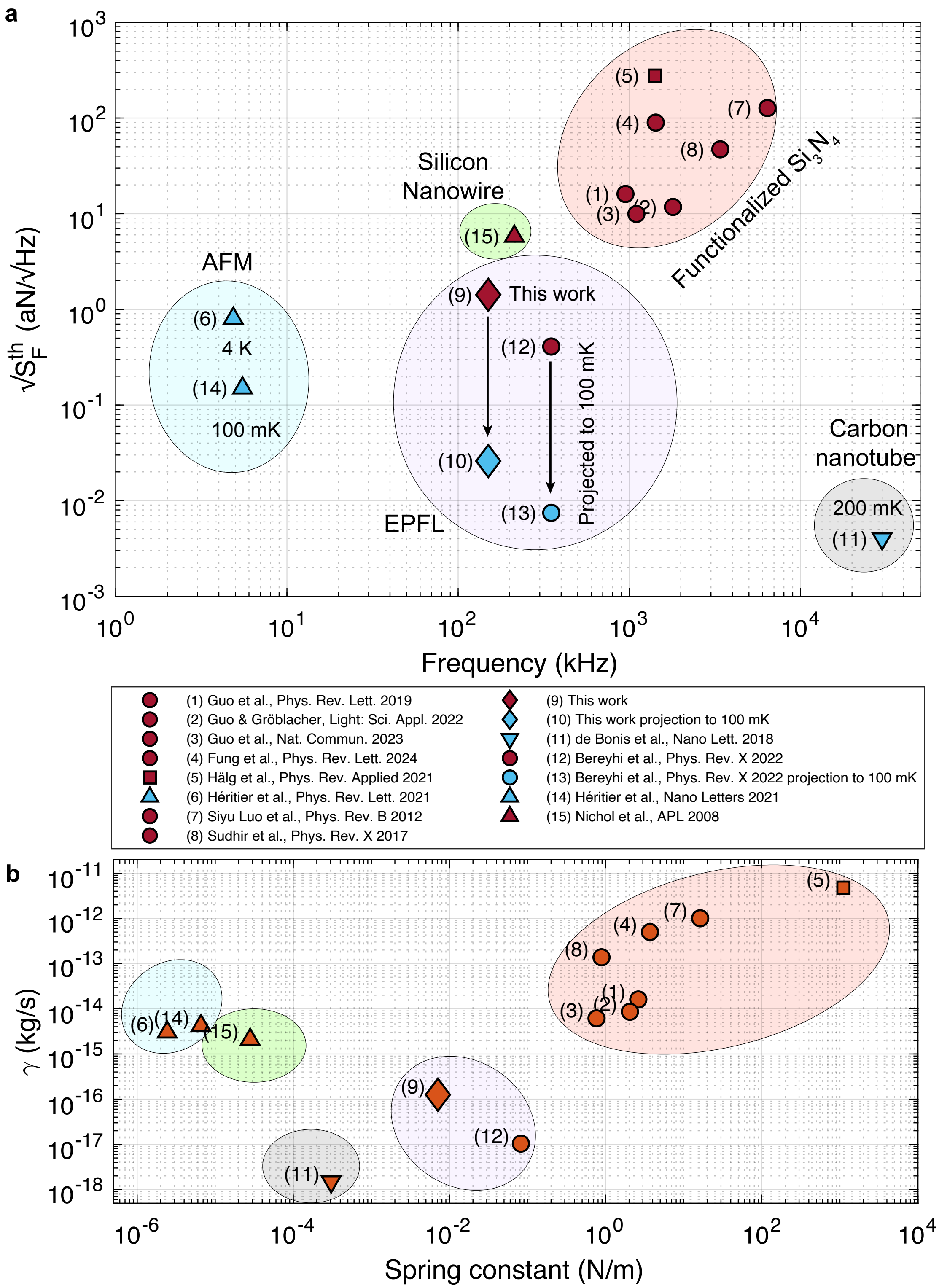

FIG. 13. **Overview of nanomechanical force sensors.a** Thermal limited force sensitivity versus mechanical frequency. **b** Dissipation coefficient versus the spring constant. In both panels red markers correspond to room temperature and blue circles to cryogenic temperature with the temperature written next to the markers.

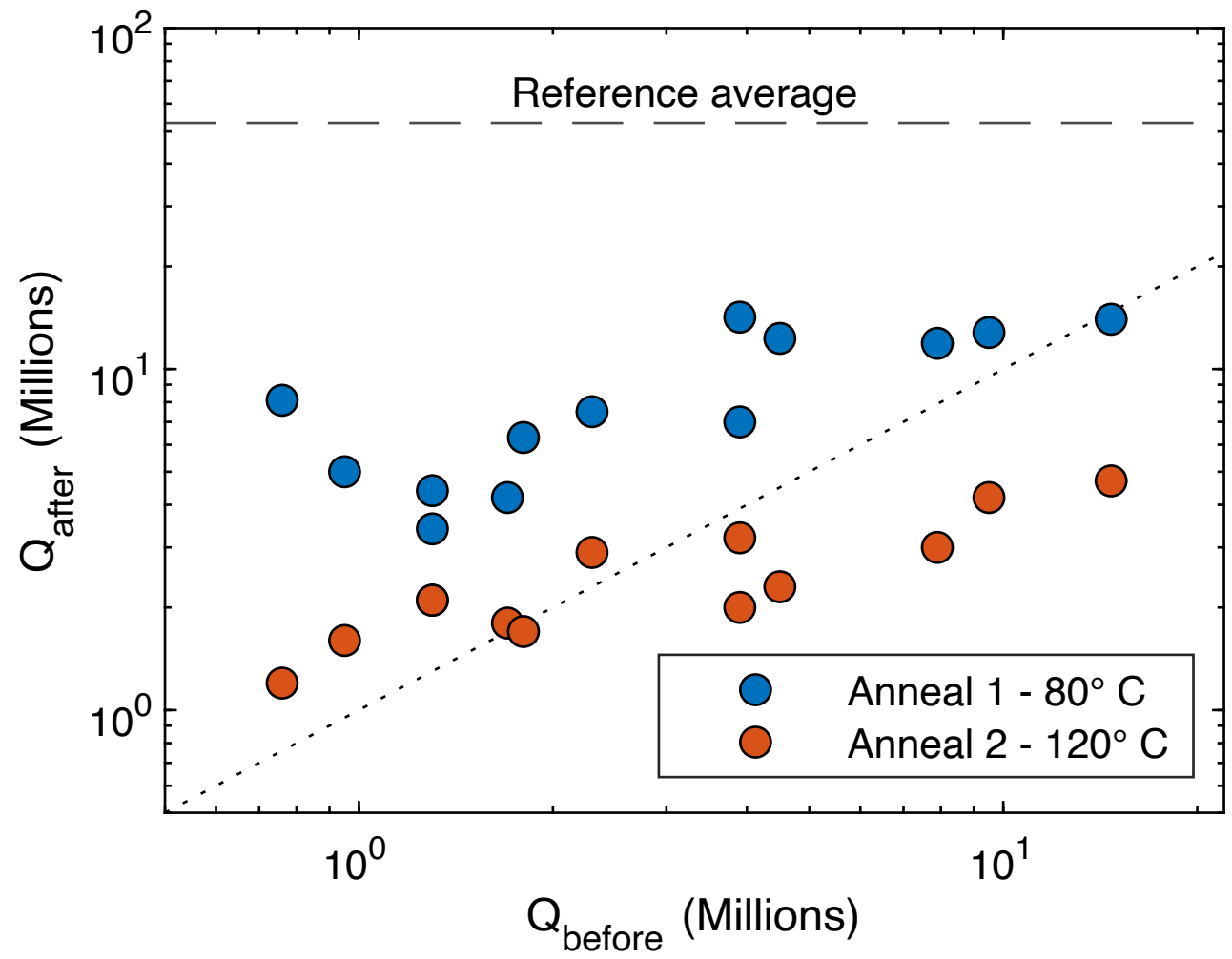

FIG. 14. **Modification of the Q upon thermal annealing.** Circles correspond to the measured quality factor after thermal annealing against the pre-bake value. The dashed line shows the average value for the reference (no PhC cavity) devices and the dotted line is an $y = x$ line for aiding the comparison.

pressure for about 5 minutes and measured the quality factor immediately afterwards. We have observed no significant improvement. The previous studies have shown that much higher power levels are required to make a significant difference [19].

---

[1] S. M. Yazdanian, J. A. Marohn, and R. F. Loring, Dielectric fluctuations in force microscopy: Noncontact friction and frequency jitter, The Journal of Chemical Physics **128**, 224706 (2008).

[2] S. Kuehn, R. F. Loring, and J. A. Marohn, Dielectric Fluctuations and the Origins of Non-contact Friction, Physical Review Letters **96**, 156103 (2006), publisher: American Physical Society.

[3] S. Kuehn, J. A. Marohn, and R. F. Loring, Noncontact Dielectric Friction, The Journal of Physical Chemistry B **110**, 14525 (2006).

[4] A. H. Ghadimi, S. A. Fedorov, N. J. Engelsen, M. J. Bereyhi, R. Schilling, D. J. Wilson, and T. J. Kippenberg, Elastic strain engineering for ultralow mechanical dissipation, Science **360**, 764 (2018).

[5] M. J. Bereyhi, A. Beccari, R. Groth, S. A. Fedorov, A. Arabmoheghi, T. J. Kippenberg, and N. J. Engelsen, Hierarchical tensile structures with ultralow mechanical dissipation, Nature Communications **13**, 3097 (2022).

[6] M. J. Bereyhi, A. Arabmoheghi, A. Beccari, S. A. Fedorov, G. Huang, T. J. Kippenberg, and N. J. Engelsen, Perimeter modes of nanomechanical resonators exhibit quality factors exceeding 10^9 at room temperature, Physical Review X **12**, 021036 (2022).

[7] D. Drysdale, *Vapour phase HF and XeF2 etching methods with improved selectivity for MEMS manufacturing*, Thesis, Heriot-Watt University (2015).

[8] A. Cupertino, D. Shin, L. Guo, P. G. Steeneken, M. A. Bessa, and R. A. Norte, Centimeter-scale nanomechanical resonators with low dissipation, Nature Communications **15**, 4255 (2024).

[9] J. Wibbeler, G. Pfeifer, and M. Hietschold, Parasitic charging of dielectric surfaces in capacitive microelectromechanical systems (MEMS), Sensors and Actuators A: Physical **71**, 74 (1998).

[10] S. Saskin, *Building Quantum Systems with Ytterbium Rydberg Arrays*, Ph.D. thesis, Princeton U. (2021).

[11] Y. Tsividis and C. McAndrew, *Operation and Modeling of the MOS Transistor* (Oxford University Press, 2011) google-Books-ID: m52JEQAAQBAJ.

[12] T. R. Albrecht, P. Grütter, D. Horne, and D. Rugar, Frequency modulation detection using high-Q cantilevers for enhanced force microscope sensitivity, Journal of Applied Physics **69**, 668 (1991).

[13] K. Y. Fong, W. H. P. Pernice, and H. X. Tang, Frequency and phase noise of ultrahigh Q silicon nitride nanomechanical resonators, Physical Review B **85**, 161410 (2012), publisher: American Physical Society.

[14] B. Kharbanda, A. Arabmoheghi, L. Catalini, M. Bereyhi, G. Benga, A. Zicoschi, C. L. Degen, T. J. Kippenberg, A. Eichler, and N. J. Engelsen, On-Chip Frequency Noise Cancellation in Nanomechanical Resonators using Cavity Optomechanics (2025), arXiv:2508.03301 [physics].

[15] A. N. Cleland and M. L. Roukes, Noise processes in nanomechanical resonators, Journal of Applied Physics **92**, 2758 (2002).

[16] S. Fedorov, A. Beccari, N. Engelsen, and T. Kippenberg, Fractal-like Mechanical Resonators with a Soft-Clamped Fundamental Mode, Physical Review Letters **124**, 025502 (2020).

[17] M. Héritier, R. Pachlatko, Y. Tao, J. M. Abendroth, C. L. Degen, and A. Eichler, Spatial Correlation between Fluctuating and Static Fields over Metal and Dielectric Substrates, Physical

Review Letters **127**, 216101 (2021).

[18] V. Sharma, C. Tracy, D. Schroder, M. Flores, B. Dauksher, and S. Bowden, Study and manipulation of charges present in silicon nitride films, in *2013 IEEE 39th Photovoltaic Specialists Conference (PVSC)* (2013) pp. 1288–1293.

[19] D. T. Krick, P. M. Lenahan, and J. Kanicki, Nature of the dominant deep trap in amorphous silicon nitride, Physical Review B **38**, 8226 (1988).
