# Supplementary material for: Non-contact friction in ultracoherent nanomechanical resonators near dielectric materials
Source: Nat Phys. 2026 Jul 7;22(9):1506–12. doi: 10.1038/s41567-026-03350-z (PMC13558075; doi:10.1038/s41567-026-03350-z)
Supplement: Supplementary file 2 [file 41567_2026_3350_MOESM2_ESM.pdf]

nature portfolio

Peer Review File

Noncontact friction in ultracoherent nanomechanical resonators near dielectric materials

Corresponding Author: Professor Tobias Kippenberg

Parts of this Peer Review File have been redacted as indicated to remove third-party material.

This file contains all reviewer reports in order by version, followed by all author rebuttals in order by version.

Attachments originally included by the reviewers as part of their assessment can be found at the end of this file.

Version 0:

Reviewer comments:

Reviewer #1

(Remarks to the Author)
Please see attached file.

Reviewer #2

(Remarks to the Author)
The manuscript "Noncontact friction in ultracoherent nanomechanical resonators near dielectric materials" describes non-contact friction effects in nanomechanical resonators designed to have extremely high mechanical quality factors. The authors concentrate on doubly-clamped beam geometries and similar geometries in which Q can be increased via the application of strain and the careful design of clamping points and mechanical mode shapes. The introduction focuses on using these devices, which the authors call 'ultracoherent mechanical devices', in sensitive force sensing applications. For this task, they emphasize the importance of reducing mechanical dissipation in order to reduce the ultimate limit to this kind of sensitive, thermal force noise. In the paper they then consider that despite the extremely low room-temperature dissipations achieved for such isolated devices so far, in any measurement applications, non-contact friction, rather than dissipation intrinsic to the resonator, will constitute the dominant mechanical dissipation.

The paper is interesting and makes a valid point that non-contact friction must be considered in any useful application of ultracoherent resonators. Although such considerations are new in this sub-field, non-contact friction is well-known in the force microscopy literature and treated in references 27 and 28, as well as other sources including Kuehn et al. PRL 96 156103 (2006), which the authors should consider citing. From this point of view, the level of novelty of the work is questionable. For example, I find on page 22, claiming that non-contact friction is a novel dissipation mechanism is over the top. Also the claim on line 64 that the loss mechanism is unexpected is misplaced, given what is known about dielectric loss from force microscopy. What can be claimed is that the authors, for the first time, fully consider this mechanism for the geometries of ultracoherent resonators. Certainly such considerations are long overdue in this sub-field. As the authors point out in line 48 "virtually all applications that require low mechanical dissipation rely on short-range interactions". So after many published papers showing record low mechanical dissipations for isolated mechanical resonators, it certainly is time to be realistic about what happens to that dissipation in the context of a measurement.

The paper is well-written, the figures are clear, and the methodology appears valid. The paper also includes and informative methods and supporting material section. Nevertheless, the conclusions are not particularly enlightening: "the presence of static charges on nanomechanical resonators gives rise to non-contact friction when they are placed near other bodies, hindering their performance in tasks that require high quality factors". This was clear in previous work on non-contact friction, mostly in singly-clamped cantilevers. Unfortunately, the paper does not provide insight on how to reduce non-contact friction in ultracoherent resonators or how to reduce the trapped charges or device imperfections that lead to it. Samples processed with metal or other coating to shield electric fields are not tested. Other methods of changing charge trapped charge configurations, such as exposure to ionizing sources are also not attempted. As a result, it is difficult to say what new physical insight the manuscript provides.

For these reasons, I suggest that the authors submit the work to a more specialized journal such as APL or PRApp.

Before publication anywhere, I would suggest that the authors also address the following points:

1) The authors clearly point our that dissipation, in their definition Q/\omega, is the limiting factor in force measurements. My naive understanding of dissipation dilution is that one can increase Q by applying tension to a clamped mechanical oscillator. Stress is applied to increase Q, by increasing the stored mechanical energy, while leaving losses unchanged. This implies that while Q should increase proportionally with stress, so should the resonant frequency \omega. This would then leave the dissipation and resulting force sensitivity unchanged. Is this incorrect? If so, why? Some intuitive explanation of these points would help clarify what can be achieved by dissipation dilution for readers outside the field.

2) I don't understand (16) below line 420 and (17) below line 425. (16) appears to be different that relevant equation in reference 12, (21), which include yeild strain. In (17) there appears to be a factor of 1/Q_{int} missing: should the intrinsic Q not go into this limit?

3) On line 20, the authors claim a "new regime of sensitivity" in the aN/Hz^(-1/2) range. Although this is a new range for room-temperature mechanical resonators, it is certainly not a new range for mechanical force sensors at low temperature, with many such devices showing aN/Hz^(-1/2) force sensitivity and some even in the 10s of zN/Hz^(-1/2), e.g. suspended carbon nanotubes in the Bachtold group (ICFO) in 2013.

Reviewer #3

(Remarks to the Author)
A. Summary of the key results

The authors measure the non-contact friction experienced by ultracoherent mechanical resonators near surfaces. The dependence of non-contract friction on frequency (i.e. oscillator mode) and distance is consistent with a loss mechanism in which static charges on the mechanical resonator interact with dielectric fluctuations in nearby surface. Three other plausible mechanisms are considered and ruled out. The measured friction data was fit to obtain charge densities and loss tangents, giving physically reasonable results.

B. Originality and significance

1. This is a seminal paper and should be published immediately with minor revisions.

2. The ultracoherent mechanical resonators studied here are among the most most force-sensitive objects ever brought into close proximity to a surface. Thus the measured non-contact frictions are milestone measurements.

3. This paper is significant because there is tremendous interest is using ultracoherent mechanical resonators in sensitive force measurements, like magnetic resonance force microscopy and electric force microscopy. This paper shows that force noise due to charges on the resonator interacting with dielectric fluctuations in the samples is a potent source of force noise. This paper suggests that the force sensitivity could be improved by reducing the static charge on the mechanical resonator.

C. Data and methodology

1. The derivations in the Supporting Information are nicely described, and are needed to understand the equations used in the manuscript. Enough information is given to reproduce the theory.

2. It would be helpful to the student if they authors could compare and contrast their theory with prior theory in S. M. Yazdanian et al. J. Chem. Phys. (2008) 128:224706, Ref. 28.

D. Appropriate use of statistics and treatment of uncertainties

Experimental uncertainties are handled appropriately.

E. Conclusions

The conclusions are sound and supported by the data.

F. Suggested improvements

1. Page 2, introductory paragraph. The introductory paragraph focusses on Q. But in many applications, the figure of merit is the dissipation $\Gamma = k /(\omega Q)$, with k the spring constant, ω the resonance frequency, and Q the quality factor. In Fig. 1(c) is would be useful if the authors could report the associated spring constant k for each of the three designs. Then the reader would have enough information to compute Γ and S_F^{th}, the power spectral density of thermal force fluctuations.

2. Page 3, line 42. Using Γ [1/s] for the damping rate instead of the dissipation constant $\Gamma = k /(\omega Q)$

[N s/m] differs from the canonical notation used in friction studies; for example, in Stipe PRL (2001), Reference 27. To avoid confusion, the authors should consider adoption Stipe's notation. They should at least point out that their notation differs from prior notation.

3. Page 4, line 71. The authors state "This phenomenon, also known as noncontact friction (NCF), has only been observed in atomic force microscopes (AFMs) cantilevers [27–29] at nanometer-scale tip-sample distances." The authors should cite the important paper by S. Kuehn et al., Phys. Rev. Lett. (2006) 96:156103, doi:10.1103/PhysRevLett.96.156103. In both Stipe 2001, Ref. 27, and Kuehn 2006, non-contact friction is apparent at distance greater than 200 nm. Similar results are seen in J. G. Longenecker et al., ACS Nano (2012) 11(6): 9637, doi:10.1021/nn3030628, where non-contact friction is apparent out to 300 nm tip-sample separation for a high-compliance cantilever over a copper microwire. So the authors should consider using more precise language than "nanometer-scale tip-sample distances".

4. Pages 6 and 7, lines 105 through 115, regarding the Fig. 1(c) calculations. To convert between the predicted quality factor Q and the fundamental underlying calculated quantity γ_{NCF}, one needs to know the spring constant k and resonance frequency ω. Could the authors please report k and ω and report and plot the associated γ_{NCF}? It is important to report γ_{NCF} so that other (future) authors can compare their measured dissipation to the measurements and calculations reported here.

5. Page 7, line 133. Before "with E and" there is "h/L". I do not understad why the ratio h/L is present in the comma-separated list on line 133. Could the authors please explain?

6. Page 8, line 145. The authors state "By using the literature values for SiO2 permittivity and loss tangent, we extract ...". Please give a citation and report the permittivity and loss tangent actually used in the calculation.

7. Page 8, lines 146, 147, and 166. In line 166, the areal charge density for Chip 1 is reported at 8.3e9 e/cm^2, which corresponds to 8.3e1 e/um^2. Given the width of 1.5 um and thickness of 0.227 um of the beam, the circumference is 3.45 um. I would thus expect a linear charge density for Chip 1 of 83 e/um^2 x 3.45 um = 286 e/um. This estimated linear charge density is in reasonable agreement with the 180 e/um reported on line 146. The authors should point out this agreement, and carry out a similar calculation for Chip 2.

8. These are impressive measurements because the sample-induced change in γ being measured here is very small. As a point of comparison, to help the reader appreciate the significance of this work, the authors could compare the sample-related changes in γ detected here to prior measurements of non-contact friction by Stipe, Kuehn, and experiments cited in the Braakman review (see below).

G. References

1. The way Refs. 16 and 17 are cited, the reader would think the references involve a magnetic resonance force microscope measurement. This is not the case. These are impressive references involve measurements of ultracoherent membranes near surfaces, but are not magnetic resonance force microscope measurements. The authors should cite additional papers in which force sensors near a surface are used to detected magnetic resonance.

2. The authors should cite the important paper by S. Kuehn et al., Phys. Rev. Lett. (2006) 96:156103, "Dielectric Fluctuations and the Origins of Noncontact Friction", doi:10.1103/PhysRevLett.96.156103. This is the first paper where, to this reviewer's knowledge, non-contact friction arising from dielectric fluctuations was first demonstrated experimentally and described theoretically.

3. Another important class of ultracoherent oscillator that the authors should cite are the silicon nanowires introduced by J. M. Nichol et al., Appl. Phys. Lett. (2008) 93: 193110, doi:10.1063/1.3025305, and reviewed by F. R. Braakman, Nanotechnology (2019) 30:332001, doi:10.1088/1361-6528/ab19cf. These nanowire oscillators have Γ values as low as 2e-15 kg/s. Can the authors compare the nanowire Γ to those of the oscillators reported here?

G. Clarity and context

1. Overall, this is a very well written paper.

2. In the abstract, replace "Here we show: it does." with "Here we show that it does."

3. Page 18, line 156. Replace "(see Methods for a discussion)." with "(See Methods ...)".

4. Page 21, line 387. Replace "for the modes with an anit-node" with "for the modes with an anti-node" (I think).

5. Page 22, line 406. Replace "vacuum friction. Van der Waals friction" with "vacuum friction, van der Waals friction".

Version 1:

Reviewer comments:

Reviewer #1

(Remarks to the Author)
Dear Authors,

Thank you very much for your constructive reply to my earlier comments and questions. I agree with your answer and I am happy to see these answers also reflected in the manuscript. Therefore, I recommend to accept the manuscript for publication.

Best regards,

Reviewer #2

(Remarks to the Author)
The revised manuscript "Noncontact friction in ultracoherent nanomechanical resonators near dielectric materials" is clearly written and the experiments and modelling appear careful and technically strong. The authors have responded conscientiously to the referees' technical requests, and the presentation has improved substantially. In that sense, I consider the work technically publishable.

My remaining concern is primarily one of framing and scope: the manuscript sometimes presents the central physical mechanism as unexpected or as a newly identified dissipation channel, whereas the underlying phenomenon (dielectric-loss–mediated noncontact friction from static charges) is well established in the AFM/force-microscopy literature. The value of the present work, in my view, is that it brings this known mechanism into the ultracoherent/dissipation-dilution nanomechanics context, quantifies its impact for state-of-the-art resonators and integrated nanophotonic geometries, and provides a modelling framework and design guidance that will be useful for that community. That is an important contribution, but it is different from a claim of discovering an unexpected dissipation mechanism.

In their response to my point that the paper lacks the novelty required of a Nature Physics article, the authors write, “what makes our work interesting for readers is the cross-fertilization of ideas in the fields of atomic force microscopes and nano-/opto-mechanics: how a loss mechanism, new to one system (i.e. optomechanical systems), turns out to unexpectedly be due to a phenomenon known in a completely different system operating in a very different regime (i.e. atomic force microscopes).” While I certainly agree that pointing out the importance of disregarded but very relevant effects to a community of researchers is important, the fact that a small sub-community has disregarded them until now, hardly makes them new or unexpected. And calling this work an example of cross-fertilization between “completely different system[s]” is an exaggeration. To someone outside of condensed matter physics, as much of the readership of Nature Physics undoubtedly is, both optomechanical systems and AFMs are sub-micron-scale mechanical systems made of similar materials subject to physics in the same limits. My own suggestion to the authors is to simply make these points clear in the paper, rather than using language which gives the false impression that something new has been discovered. The authors have already taken most of this out, although ‘unexpected loss mechanism’ remains on line 64 of page 4.

In terms of significance, as the authors point out in their lengthy reply, the paper makes several important technical contributions including, measuring NCF with high precision, confirmation in observations on ‘ultracoherent resonators’ of the effects of NCF, design level insight into how to optimize force sensitivity in view of NCF., and perhaps most importantly detailed theoretical and numerical modelling of NCF. However, simulating and experimentally confirming the importance of dielectric loss in these mechanical resonators does not constitute a new physical discovery. As the authors themselves recount in their response, this was established long ago in AFM in systems that are essentially the same, except for minor technical differences (size, geometry, purpose). The difference in inter-body distances pointed out by the authors is specious because it is largely offset by a difference in resonator size and interaction area, which are also involved in these effects. In fact, two referees point out the same seminal papers for the authors to cite in their work (especially Yazdanian, Keuhn, and Stipe) on this loss mechanism, showing that the underlying physics is well-established. It was understood and described in these papers in a very general sense and anyone with knowledge of this literature would expect the same mechanisms to play important roles as ‘ultracoherent resonators’ are brought near surfaces. In this sense, what is described is not new; it is, however, valuable to clearly point out to a community that for too long has ignored these points.

Ultimately, whether the work meets the particular breadth/novelty threshold for Nature Physics (as opposed to a more specialized venue) is an editorial judgement; my view is that its strongest contribution is the quantitative translation and design-relevant modelling of a known dissipation process into the ultracoherent nanomechanics setting, rather than the discovery of a new or surprising mechanism.

Reviewer #3

(Remarks to the Author)
A. Summary of the key results

The revised manuscript is suitable for publication in Nature Physics.

The reviews were reasonable. The authors have responded thoroughly and thoughtfully to all reviewer comments.

B. Originality and significance

As the third reviewer, I agree with all but two of the criticisms of the first two reviewers.

I disagree with Reviewer 1 that temperature-dependent measurements are needed for a decisive test. The paper's comparison between theory and experiment is already innovative and conclusive. The editor should appreciate that the authors are measuring non-contract friction in new regime of low loss, with a precision that is orders of magnitude better than all prior room-temperature measurements. Variable temperature measurements require a new apparatus and are expensive and painful; it is not reasonable to require them at this stage.

Reviewer 2 raised concerns about the significance of the work. The authors have done an excellent job addressing these concerns in both the reviewer-response document and the revised manuscript. The revised manuscript describes the work in a way that makes it clear the work is a significant advance. I therefore disagree with Reviewer 2's assessment that the manuscript is more appropriate for "a more specialized journal". Ultrasensitive force measurements are central to physics and this paper presents milestone results in force detection. The results deserve to be published in a premier journal.

The new sections on "Finite electrical conductivity" (manuscript) "D. Effect of finite conductivity" (supplementary information) are significant additions and strengthen the conclusions of the manuscript: the observed non-contact frictions is not due to conductive losses.

E. Conclusions

1. The new leading sentence of the conclusion is a big improvement that will help the general reader understand the significance of this work.

2. The new last two sentences are reasonable.

F. Suggested improvements

1. In Fig. 6 of the reviewer-response document, and Fig. 5 in the revised supplementary information, I believe that the spring constant labels in the plot should have units of N/m, not N m. I believe this mislabeling is a typographical error.

2. If the reviewer-response document is going to be published, there are a few typographical errors to fix. Throughout, replace (a) "Stippe" with "Stipe" and (b) "margine" with "margins" or "margin".

Open Access This Peer Review File is licensed under a Creative Commons Attribution 4.0 International License, which permits use, sharing, adaptation, distribution and reproduction in any medium or format, as long as you give appropriate credit to the original author(s) and the source, provide a link to the Creative Commons license, and indicate if changes were made.
In cases where reviewers are anonymous, credit should be given to 'Anonymous Referee' and the source.

The images or other third party material in this Peer Review File are included in the article's Creative Commons license, unless indicated otherwise in a credit line to the material. If material is not included in the article's Creative Commons license and your intended use is not permitted by statutory regulation or exceeds the permitted use, you will need to obtain permission directly from the copyright holder.
To view a copy of this license, visit https://creativecommons.org/licenses/by/4.0/

Response to the reviewers' comments for:
Noncontact friction in ultracoherent nanomechanical resonators near dielectric materials

Amirali Arabmoheghi,[1,*] Alessio Zicoschi,[1] Guillermo Arregui,[1] Mohammad J. Bereyhi,[1] Yi Xia,[1] Nils J. Engelsen,[1,2] and Tobias J. Kippenberg[1,†]

[1] *Swiss Federal Institute of Technology Lausanne (EPFL), CH-1015 Lausanne, Switzerland*

[2] *Department of Microtechnology and Nanoscience (MC2), Chalmers University of Technology, SE-412 96 Göteborg, Sweden*

(Dated: December 16, 2025)

The points raised by the reviewers are addressed in the following. The original text by the reviewers is written in *italic blue* and our reply is written in black text. The amendments to the manuscript are also noted in red.

I. REVIEWER 1

The authors present a compelling experimental and theoretical study identifying non-contact friction (NCF) as a new dissipation channel limiting the quality factor of ultracoherent Si_3N_4 nanomechanical resonators placed near dielectric materials. The work is timely and relevant to the rapidly expanding field of quantum nanomechanics, where understanding loss mechanisms is critical to achieving fundamental sensitivity limits. The experimental data are of high quality and supported by rigorous analytical and FEM modelling. The paper is generally well written and provides valuable insights into how trapped charges and dielectric losses can influence resonator coherence.

Nevertheless, several aspects deserve further consideration or clarification before the work can be accepted in Nature Physics:

We thank the reviewer for their positive assessment of our work and the constructive suggestions that are discussed below.

1. ***Assumption of static charges.***

   The analysis assumes fixed (time-independent) surface or volume charge densities on the resonators and dielectric substrates. While this is a reasonable first approximation, amorphous dielectrics such as Si_3N_4 and SiO_2 are known to host charge traps that allow slow hopping or tunnelling processes. Such dynamics can give rise to charge diffusion and Coulomb drag between neighboring defects, producing frequency-dependent permittivity and dissipation that may mimic or enhance the observed $1/\Omega$ scaling. A discussion of whether the inferred loss tangent and effective charge densities are consistent with charge- transport-limited relaxation would strengthen the physical interpretation. The authors could, for example, model the effect of finite charge mobility ($\sigma \neq 0$) on Eq. (2) and show how it modifies γ_{NCF} versus frequency. This would clarify whether

* amirali.arabmoheghi@epfl.ch
† tobias.kippenberg@epfl.ch

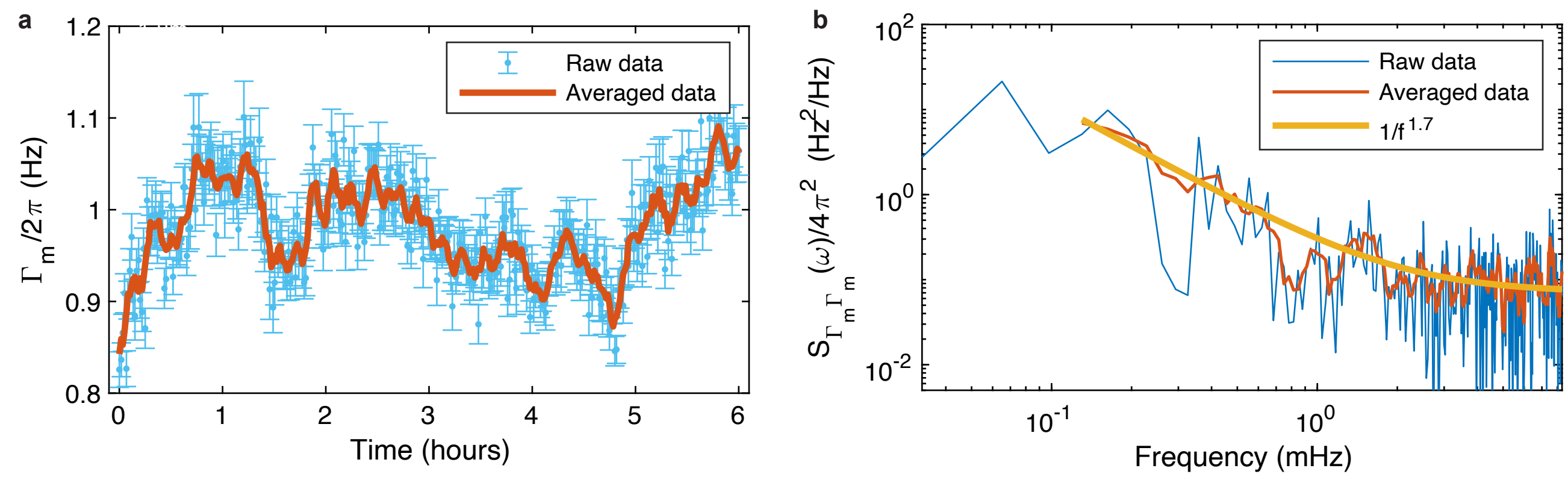

FIG. 1. **Slow temporal variations of NCF.** **a** Measurement record of damping rate of the fundamental mode of a 2.5 mm string from chip 1 of Fig. 2 of the main text over 6 hours in 1 minute intervals. Blue markers: result of an exponential fit to each ringdown measurement with error bar corresponding to the fitting error. Orange line: A moving average over the data. **b** Power spectral density (PSD) of the measured damping rate in **a**. Blue line: PSD of the raw data. Orange line: PSD of the moving average. Yellow line: A power law fit to the averaged data.

the observed damping originates purely from static electrostatics or from correlated hopping and dielectric relaxation processes.

We thank the reviewer for raising an interesting point. As they correctly mention, in our paper we study NCF arising from motion of mechanical resonators, that are charged with *static* charge densities, near dielectrics with only dielectric (i.e. polarization) losses. We have considered no charge mobility as all the materials used in our experiments are good insulators. However, as the reviewer correctly points out, in amorphous materials and particularly glass (i.e. SiO_2), slow dynamics of trapped charges and tunneling events can give rise to a non-vanishing charge mobility [1]. Having charge mobility (or conductivity) can also contribute to NCF. We can readily generalize our model to include a finite charge mobility as another loss channel. We discuss finite charge mobility further in detail in response to the second point raised by the reviewer.

Our data shows that while the slow dynamics of charge traps does not affect the dependence of NCF on the mechanical modes' frequencies, these phenomena come into play at much slower time scales. Throughout our experiments, for devices with

NCF-limited mechanical Q, we have observed slow drifts of the measured Qs. From the devices presented in section 3 of the main text, we pick one of the devices whose Q is dominated by NCF. This device is from Chip 1 and its fundamental mode has the frequency of 100 kHz. Via repeated ringdown measurements, we measure the damping rate of this string for several hours in minute intervals. Fig. 1(a) shows the outcome of these measurements, showing variations up to 30% over time. In Fig. 1(b) we show the power spectral density of the variations of the measured damping rates. At low frequencies, we fit a power law scaling of $S_{\Gamma_m\Gamma_m}(\omega) \propto \omega^{-1.7}$. Similar to the flicker noise in electronics, these power law variations are typically a signature of trapping dynamics (for example see chapter 8 of Ref. [2]). To conclude that the observed fluctuations are due to charge fluctuations and the interaction with the substrate, the data here has to be compared with Γ_m fluctuations of a fully released (i.e. no earby substrate) string of similar dimensions. As we do not have such a device we cannot make this concrete comparison. However, since the damping rate of this device is dominated by NCF by a large margine (the NCF-limited Q is about 2 orders of magnitude smaller than the bending-limited one), we can argue that the observed fluctuations are due to fluctuations in NCF, which is most likely due to charge dynamics.

Another signature of the slow dynamics is observed in the mechanical frequency fluctuations of the binary-tree strings in the nano-optomechanical devices. Using an established technique [3], we measure the frequency fluctuations of the mechanical modes of these devices using our free-space interferometer. In this technique, the mechanical mode is continuously and resonantly driven by a piezoelectric actuator, driven by a local electronic oscillator. The displacement measurement signal obtained from the interferometer is electronically demodulated using the same local oscillator, which allows phase detection at the frequency of the mechanical mode. The detected phase is fed to a phase-locked loop which phase-locks the local oscillator to the mechanical mode that eventually yields a real-time record of the frequency fluctuations of the mechanical mode. This procedure is implemented using the built-in functionalities of a Zurich Instruments lock-in amplifier. We have studied the mechanical frequency fluctuations using 3 reference (gap = ∞) and multiple integrated devices with gaps of 200, 300 and 400 nm). Measurement of the quality factors of the fundamental modes of these devices at $\Omega_m = 180$ kHz (shown in Fig. 2(b)) reveals that the integrated devices are

indeed affected by NCF. In Fig. 2(c) we show the power spectral density of the measured frequency fluctuations for these 6 devices. The frequency noise, $S_{\Omega_m\Omega_m}(\omega)$ at frequencies above 20 Hz (i.e. $\omega/2\pi > 20\,\text{Hz}$), is dominated by imprecision (detection) noise in the displacement measurement. For lower frequencies the noise corresponds to the frequency fluctuations of the mode. For the reference devices (gap $= \infty$), we observe that all the 3 devices closely follow a flicker-type $1/\omega$ noise with the same magnitudes. This flicker frequency noise has been repeatedly observed in nanomechanical resonators (for example see [4] and [5] for Si_3N_4 resonators) and is typically referred to as the intrinsic frequency noise and is explained by the reorientation dynamics of defects in the solid body of the resonator [6]. Interestingly, for the integrated devices, we observe at least 10 times higher level of the $1/\omega$ noise. We believe that these excess fluctuations are due to the interaction of the mechanical resonator with the defects or charge traps within the Si_3N_4 photonic crystal cavities. For other ensembles of devices, although we have not observed a clear scaling with the gap size, we have consistently observed that the integrated devices exhibit a higher level of the $1/\omega$ noise.

In summary, we believe that slow defect dynamics present in the amorphous materials used in our experiments do not contribute to NCF as they do not introduce significant dissipation at the frequency of the mechanical modes. However observation of flicker-type variations of the NCF-limited damping rates in the strings suspended above SiO_2 and frequency fluctuations in the nano-optomechanical devices testify the presence of such dynamics at sub-hertz frequency scales.

Amendments to the manuscript

These two observation alongside the figures are added to the supplementary information under a new section entitled “SIGNATURES OF SLOW CHARGE DYNAMICS”.

2. ***Role of Coulomb drag in dielectric films.***

The native oxide and Si_3N_4 layers possibly contain mobile ionic or electronic carriers that can be displaced by the resonator's oscillating field. Such Coulomb-drag-type effects would manifest as temperature- and frequency-dependent dielectric losses. The manuscript currently attributes all dissipation to static polarization loss, but consid-

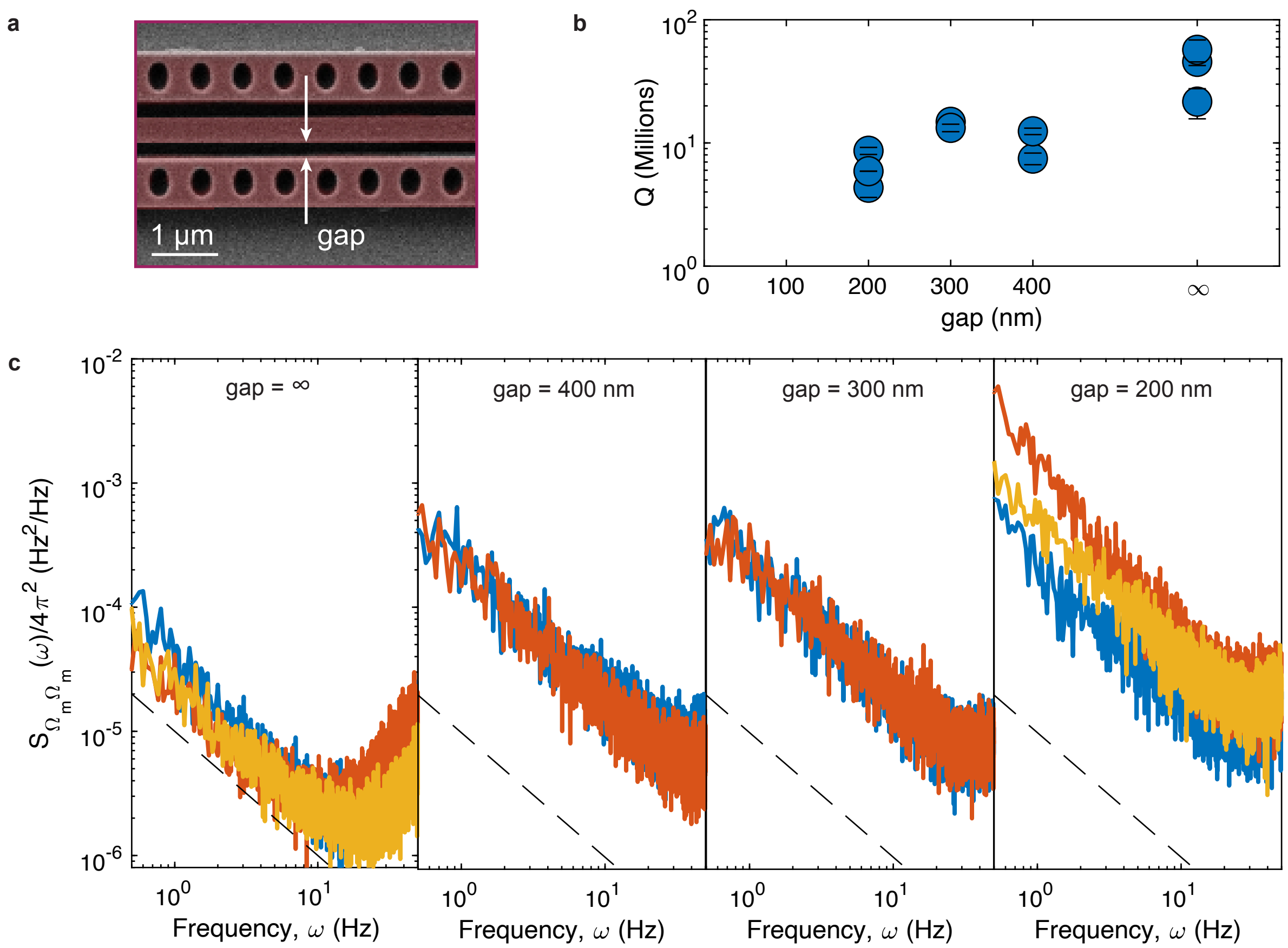

FIG. 2. **Gap dependent mechanical frequency noise.** **a** false colored SEM of the integrated device (same as Fig.3(b) of the main text). **b** Measured Q of reference and integrated binary-tree devices. **c** Power spectral density of the mechanical frequency fluctuations of the 10 devices in **b**. Dashed line: a guide to the eye for visualizing the $1/\omega$ scaling.

eration of these drag contributions is important to discriminate between defect-related and intrinsic dielectric mechanisms.

We thank the reviewer for their comments. Generally, studying further the origin of dissipation within the dielectrics surrounding the resonators is an interesting quest. In fact in the community of atomic force microscopes (AFMs) many different mechanisms beyond polarization loss have been proposed and observed. These mechanisms include electrical dissipation [7], magnetic dissipation [8, 9] and myriad near-field thermal phenomena reviewed in Ref [10]. We indeed concur with the reviewer's previous comment that for the materials used in our work (i.e. Si_3N_4 and SiO_2), beyond the polarization loss, the next approximation would be conductive losses. No matter what

is the conduction mechanism (e.g. charge traps or Coulomb drag), as the reviewer has suggested in the previous comment, we can model the charge mobility by assuming a finite conductivity in the materials ($\sigma \neq 0$). To model the NCF coefficient arising from the finite conductivity, $\gamma_{\mathrm{NCF}}^{\mathrm{cond}}$, we can follow a similar procedure as the one we have used for dielectric losses. As described in detail in our supplementary information, for a harmonic electric field, in the case of dielectric loss, the period-averaged dissipated power density is given by $\bar{p}_d(\vec{r}) = 2\omega\epsilon_0\epsilon_r''(\vec{r},\omega)|\vec{E}(\vec{r},\omega)|^2$. For the case of conductive losses, the dissipated power density is given by $\bar{p}_d^{\mathrm{cond}}(\vec{r}) = 2\sigma(\vec{r},\omega)|\vec{E}(\vec{r},\omega)|^2$, where $\sigma(\vec{r},\omega)|$ is the conductivity. This dissipation gives rise to the NCF coefficient

$$\gamma_{\mathrm{NCF}}^{\mathrm{cond}} = \int d^3r\, |\vec{g}(\vec{r},\vec{r};\vec{U}_m(\vec{r}))|^2 \frac{\sigma(\vec{r},\Omega_m)}{\Omega_m^2}. \tag{1}$$

We have added an SI section describing this derivation in detail. As opposed to the $1/\Omega_m$ frequency scaling of Eq. (2) of the main text, which is also consistent with our observations, NCF due to conduction has the frequency scaling of $1/\Omega_m^2$. As a result, unless the conductivity scales strictly linearly with frequency, i.e. $\sigma \propto \omega$, in our frequency range of interest, the observed scaling cannot be consistent with conductivity-dominated NCF. In previous measurements of the conductivities of SiO_2 and stoichiometric Si_3N_4 [11, 12], we do not find such linear frequency scalings. Moreover, the values reported for the conductivities are in the $10^{-14}\ \Omega^{-1}\mathrm{m}^{-1}$ range. At our mechanical frequencies, these values would amount to loss tangents ($\tan\delta = \sigma/\epsilon_0\epsilon_r\Omega_m$) on the order of 10^{-9} which is far bellow what we have estimated. We have included these arguments in our methods section for ruling out conductive losses as the origin of the observed NCF in our methods section.

In summary, losses due to mobile charges give rise to a frequency scaling different than $1/\Omega_m$ for NCF. Consequently, the only way in which conduction losses can explain the $1/\Omega_m$ scaling is that the conductivity scales linearly with frequency for both SiO_2 and Si_3N_4. Otherwise, our model involving static charges in conjunction with dielectric losses remains the best model for explaining our observations.

Amendments to the manuscript

As per the suggestion of the reviewer, we have added a new SI section which demonstrates this derivation, its validity regime and the ensuing correction to the Eq. (2) of the main text. We have also added a new subsection to the discussion of other possible loss mechanisms in our methods section where we argue why conduction cannot explain our observations.

3. ***Temperature-dependent measurements.***

The entire data set is taken at room temperature. Measuring Q versus temperature (for instance between 10 K and 300 K) could decisively test whether the relevant charges are trapped electrons, ionic species, or surface adsorbates. A decrease of NCF with decreasing T would indicate thermally activated hopping, whereas persistence of the same scaling would support a purely electrostatic origin. Even a single low-temperature data point on the same devices would reinforce the conclusions.

We thank the reviewer for suggesting low temperature measurements as they can potentially be interesting. As the reviewer is aware, cryogenic experiments are time consuming are not straightforward to carry out. We therefore consider such measurements beyond the scope of our work at this time. However, we are in the process of building an experimental setup for performing cryogenic optomechanics experiments with our nano-optomechanical system. Studying low temperature behavior of NCF in our system will be an important part of those experiments. Should we accomplish the construction of our cryogenic setup in a timely manner, we are more than happy to provide data from temperature-dependent measurement. Nevertheless, as we have strong evidence that NCF originates from the the dielectric loss of (in our case) amorphous materials, we expect to observe two-level-system-like temperature dependence at low temperatures [13]. As an example, for Si_3N_4, measurements using superconducting circuits at GHz frequencies [14] confirm this behavior.

In summary, this is a technically impressive and potentially field-defining work, but further discussion of charge dynamics and temperature dependence is needed to unambiguously identify the microscopic origin of the non-contact friction. I recommend major revision,

focusing on the physical nature (static vs. mobile) of the charges and possible experimental validation through temperature-dependent Q-factor measurements.

We appreciate the reviewer's recognition of our work and their constructive suggestions. We hope that the reviewer finds our arguments and experimental evidence insightful. We concur with the general assessment of the reviewer that a more profound understanding of the nature of the charges or the losses within the dielectrics can be interesting. However, we believe that such studies require experimental platforms that are particularly designed for these tasks. As we have mentioned in our manuscript, we believe that our work already goes beyond identifying NCF as a limiting factor for ultracoherent nanomechanical resonators and introduces tools for probing material properties with a new regime of sensitivity. We believe that our experimental and theoretical techniques indeed permit experiments that allow for a deeper dive into the domain of material science and can be interesting future directions.

II. REVIEWER 2

The manuscript "Noncontact friction in ultracoherent nanomechanical resonators near dielectric materials" describes non-contact friction effects in nanomechanical resonators designed to have extremely high mechanical quality factors. The authors concentrate on doubly-clamped beam geometries and similar geometries in which Q can be increased via the application of strain and the careful design of clamping points and mechanical mode shapes. The introduction focuses on using these devices, which the authors call 'ultracoherent mechanical devices', in sensitive force sensing applications. For this task, they emphasize the importance of reducing mechanical dissipation in order to reduce the ultimate limit to this kind of sensitive, thermal force noise. In the paper they then consider that despite the extremely low room-temperature dissipations achieved for such isolated devices so far, in any measurement applications, non-contact friction, rather than dissipation intrinsic to the resonator, will constitute the dominant mechanical dissipation.

We thank the reviewer for considering our work. The raised concerns are addressed in the following.

The paper is interesting and makes a valid point that non-contact friction must be considered in any useful application of ultracoherent resonators. Although such considerations are

new in this sub-field, non-contact friction is well-known in the force microscopy literature and treated in references 27 and 28, as well as other sources including Kuehn et al. PRL 96 156103 (2006), which the authors should consider citing. From this point of view, the level of novelty of the work is questionable. For example, I find on page 22, claiming that non-contact friction is a novel dissipation mechanism is over the top. Also the claim on line 64 that the loss mechanism is unexpected is misplaced, given what is known about dielectric loss from force microscopy. What can be claimed is that the authors, for the first time, fully consider this mechanism for the geometries of ultracoherent resonators. Certainly such considerations are long overdue in this sub-field. As the authors point out in line 48 "virtually all applications that require low mechanical dissipation rely on short-range interactions". So after many published papers showing record low mechanical dissipations for isolated mechanical resonators, it certainly is time to be realistic about what happens to that dissipation in the context of a measurement.

We appreciate the reviewer's careful assessment of our work from a line-by-line to a high level and also suggesting references. We also regret to see that some of our claims appear misleading to them.

Throughout our manuscript, we have made it clear that we are adapting many concepts from the findings of the AFM community. As the title suggests, our manuscript reports the observation of noncontact friction (a term coined by the AFM community), in nanomechanical resonators suspended near other objects. There is no doubt that the essence of dielectric-loss-induced mechanical dissipation is well known in the AFM community. However, we believe what makes our work interesting for readers is the *cross-fertilization* of ideas in the fields of atomic force microscopes and nano-/opto-mechanics: how a loss mechanism, *new* to one system (i.e. optomechanical systems), turns out to *unexpectedly* be due to a phenomenon known in a completely different system operating in a very different regime (i.e. atomic force microscopes). Interesting advances in science are often of this nature, but the connection between two fields or concepts that seems trivial or obvious in retrospective, is never initially clear.

A good example is the observation of NCF in AFM systems itself. Studying unidentified loss mechanisms in any physical system is always challenging and often involves misinterpretations as there are typically many sources of loss with dramatically different natures. Atomic-force microscopes were designed to probe sample properties via measuring the shifts

in resonance frequency of a cantilever due to atomic forces. As the cantilevers were improved to have higher quality factors, experiments observed modification of the cantilever's damping rate as well. In retrospective it is not surprising that an apparatus that was designed to measure surface properties using *dispersive* effects, when sufficiently sensitive, eventually measures *dissipative* effects as well. However, this narrative completely ignores the historical nuances and the methodologies used to actually understand NCF. Already in 1991, it was clear that moving a charged high-Q cantilever close to a sample leads to changes in the mechanical quality factor [7]. Despite this notion, after nearly 10 years very similar observations where misinterpreted to be originating from purely thermal fluctuations of the electromagnetic field in the short tip-sample gap [15]. It was not until the work of Stippe et al. in 2001, [16] and eventually the works of Kuehn et al. [17] and Yazdanian et al. [18] in 2006 and 2008 that the connection to dielectric losses was made and NCF in AFM systems was fully understood. In the 2000s, everyone was obviously aware of dielectric dissipation but identifying it as the origin of NCF was the important contribution of the authors mentioned above.

A short recap of the literature of the optomechanics and suspended nanomechanics communities is equally helpful to have a better assessment of the novelty of our work. In particular understanding why observation of NCF in a system like the one in our work was not at all foreseen. Silicon nitride nanomechanical resonators have been central to research in optomechanics and nanomechanics for at least 20 years and, despite known to have surface charges [19], there is not a single mention—to the best of knowledge—of the possible adverse effects of the trapped charges, let alone predicting the occurrence of NCF. In particular in systems with Si_3N_4 strings suspended close to SiO_2 micro-disks/toroids [20, 21] and Si_3N_4 photonic crystal cavities [22], NCF is neither observed nor discussed. Even a very recent observation of reduction of the Q of a high-Q string in a near-field optomechanical system [23] was poorly understood and ascribed to a mechanism that we show does not play a role (see line 390 of our methods section). We believe the reduction observed in that work was most likely due to NCF. Considering these narratives from groups that have years of experience in this field, we believe that it is not *misplaced* or *over the top* to say that observation of NCF was indeed unexpected and novel for string resonators. We would argue that even for an AFM expert who is familiar with NCF and its dependence on the frequency and distance, predicting its observation *a priori* for a millimeter scale resonator

would have been difficult given that the resonator is not *intentionally* charged, has a resonance frequency about 50 times larger than a typical AFM cantilever and is 0.6 μm far from a tiny photonic crystal cavity (as opposed to AFM systems that are tens of nanometers above a *semi-infinite* sample). Furthermore, in the case of ultracoherent strings with record value quality factors [24–28], for low-frequency modes that are expected to have higher Q in none of the cited works they actually reach the simulation values. In Figure 1(c) in the main text, we show that this disagreement is also most likely due to NCF with the substrate that is not hundreds of nanometers, but tens of micrometers away. It is indeed not over the top to say that this finding was unexpected.

Based on the above, we believe that our claim of novelty of this mechanism in the domain of ultracoherent nanomechanical oscillators is well supported. However, we understand how the sentence in the abstract in line 22 could be misinterpreted as a global claim. In the resubmitted manuscript the word novel has been omitted from this sentence.

Amendments to the manuscript

- As per the suggestion of the reviewer, we have cited the paper Kuehn et al. PRL 96 156103 (2006) in the introduction in line 73.

- In the sentence in line 22, in the abstract: *We report a novel dissipation mechanism that occurs in ultracoherent nanomechanical oscillators caused by the presence of nearby dielectrics.*, the word *novel* is omitted.

The paper is well-written, the figures are clear, and the methodology appears valid. The paper also includes and informative methods and supporting material section. Nevertheless, the conclusions are not particularly enlightening: "the presence of static charges on nanomechanical resonators gives rise to non-contact friction when they are placed near other bodies, hindering their performance in tasks that require high quality factors". This was clear in previous work on non-contact friction, mostly in singly-clamped cantilevers. Unfortunately, the paper does not provide insight on how to reduce non-contact friction in ultracoherent resonators or how to reduce the trapped charges or device imperfections that lead to it. Samples processed with metal or other coating to shield electric fields are not tested. Other methods of changing charge trapped charge configurations, such as exposure to ionizing sources are also

not attempted. As a result, it is difficult to say what new physical insight the manuscript provides.

We thank the reviewer for endorsing our methodology and communication. We would like to reiterate some of our main achievements that could help the reviewer to see the physical insights contained in our work.

- **Measuring NCF with a new precision level**

  Our work demonstrates measurement of NCF dissipation at the level of 10^{-16} kg/s, which is 2 to 3 orders of magnitude lower than what has been reported by Stippe et al. and Kuehn et al. [16, 17]. From a purely experimental point of view, especially given the distances at which we have measured these values, this is a new regime for measurement of NCF, which enables further capacities for studying material properties (see the reply to Reviewer 1). This is emphasized in our conclusions.

- **Theoretical and numerical modeling**

  The theoretical modeling of NCF in the AFM community [17, 18] and also trapped ion systems [29], to the best of our knowledge, has never gone beyond a point-like charge in one dimensional motion above a semi-infinite substrate. In our work, we extend the theory to arbitrary charge distributions on structures with arbitrary geometries and mechanical modes with generic loss mechanisms. We have used these methods to rigorously model NCF in two different physical systems, both theoretically and numerically which has enabled us to extract *microscopic* properties such as surface charge densities only from *macroscopic* observations such as quality factor measurements. We would also like to highlight that the manuscript provides a practical recipe for estimating NCF using a common FEM solver in the nanomechanics and related research communities.

- **Insights about trapped charges**

  With the ever increasing interest for scalable systems for quantum science and technology, many research directions are shifting towards miniaturization of systems, that where originally developed in bulk systems, on micro-fabricated chips. However, the focus on designing systems that implement a certain Hamiltonian with strong interaction often distracts us from attention to the common challenges of fabrication and

miniaturization such as surface charges. As an example mentioned earlier, the presence of charges on the surface of Si_3N_4 was known since the 80s but in the communities of photonics, nanomechanics and optomechanics who use Si_3N_4 across tens of different platforms the issues related to charges are not deeply discussed. We believe our work exemplifies meticulous treatment of such problems that typically are considered less *fundamental* by experts in AMO physics, in a language that is accessible for a broad audience and can be insightful across different disciplines.

- **Cross-platform and reproducible observations**

  We believe another aspect of our work that can be enlightening for the readers is how paying attention to *imperfections* across many samples can lead to uncovering hidden physics. A lot of experiments that require micro-fabricated devices with exceptional performances (for example ultracoherent nanomechanics or superconducting qubits with long lifetimes), often rely on one *hero* sample and all the other tens of *subpar* samples are discarded or ignored. For example, in our nano-optomechanical devices in Fig. 3(e), for the device with $d = 600$ nm with the highest Q that is less than a factor 2 lower than the expected value, the Q reduction could have been ignored and the device could be used for the optomechanics experiments. However, carefully characterizing all the 77 devices in this figure prompted the whole study that lead to uncovering interesting physics hidden in our system. In addition, a similar approach in an apparently different platform (i.e. uniform strings above SiO_2) led to observations and results that are consistent with the other platform. We believe, in this capacity, our work can be inspiring to a broad range of audience who use micro-fabricated devices.

- **Creative methodology and data analysis**

  We believe the practical and technical methods used in our work to make the best use of the capabilities of our platforms can also be insightful for a broader audience. For example, early in our studies, upon discussions with AFM experts, we were encouraged to build experiments that allow for in situ tuning of the gap. However, this would take the complexity of our device fabrication and experimental setups to another level. Instead, we found that the $1/\Omega_m$ frequency dependence is a rather unique aspect of NCF physics that, despite being known in the AFM community, has not received much

attention compared to gap scalings in previous works. This is despite the fact that, as apparent from Eq. (2), the geometrical dependence of NCF can be very complicated and it is often challenging to calibrate experimentally with good precision, while the $1/\Omega_m$ scaling is rather universal and a more experimentally accessible quantity. We believe that this aspect of our work, which encourages creativity, will be inspiring for the readers.

- **Trade-off between NCF and dissipation-dilution**

  We would like to emphasize that our work provides a key design-level physical insight that is directly relevant for future generations of ultracoherent resonators: it reveals a fundamental trade-off between dissipation-dilution-limited losses and non-contact-friction-limited losses. Because these two dissipation mechanisms exhibit opposite scalings with the mechanical frequency, their competition necessarily gives rise to an optimal operating frequency at which the total mechanical dissipation is minimized. The most desirable mitigation strategy for NCF is indeed reducing the charge densities. However, finding an effective recipe requires dedicated studies. The trade-off discussed in our work, determines the regime of sensitivity for ultracoherent nanomechanical resonators that is achievable without relying on discharging.

Following the reviewer's comment, we have modified the conclusion paragraph in a way that it better reflects some of the key insights in our work, in particular highlighting the 2-3 orders of magnitude lower amount of NCF measured here in comparison with cantilevers.

Amendments to the manuscript

We have modified the conclusion paragraph for better clarity.

Regarding mitigation strategies, it is also important to note that our work is the first to introduce a method for estimation of NCF in arbitrary nanomechanical resonators. To find an effective mitigation strategy for NCF in these systems, one needs to be able to estimate it first. We believe our work takes the necessary first step towards this goal. Nevertheless, two mitigation strategies are tested and discussed in the last section of our supplementary information. We have employed two commonly used annealing techniques, thermal and UV, that has been shown to be effective in the past for *purifying* silicon nitride. We have

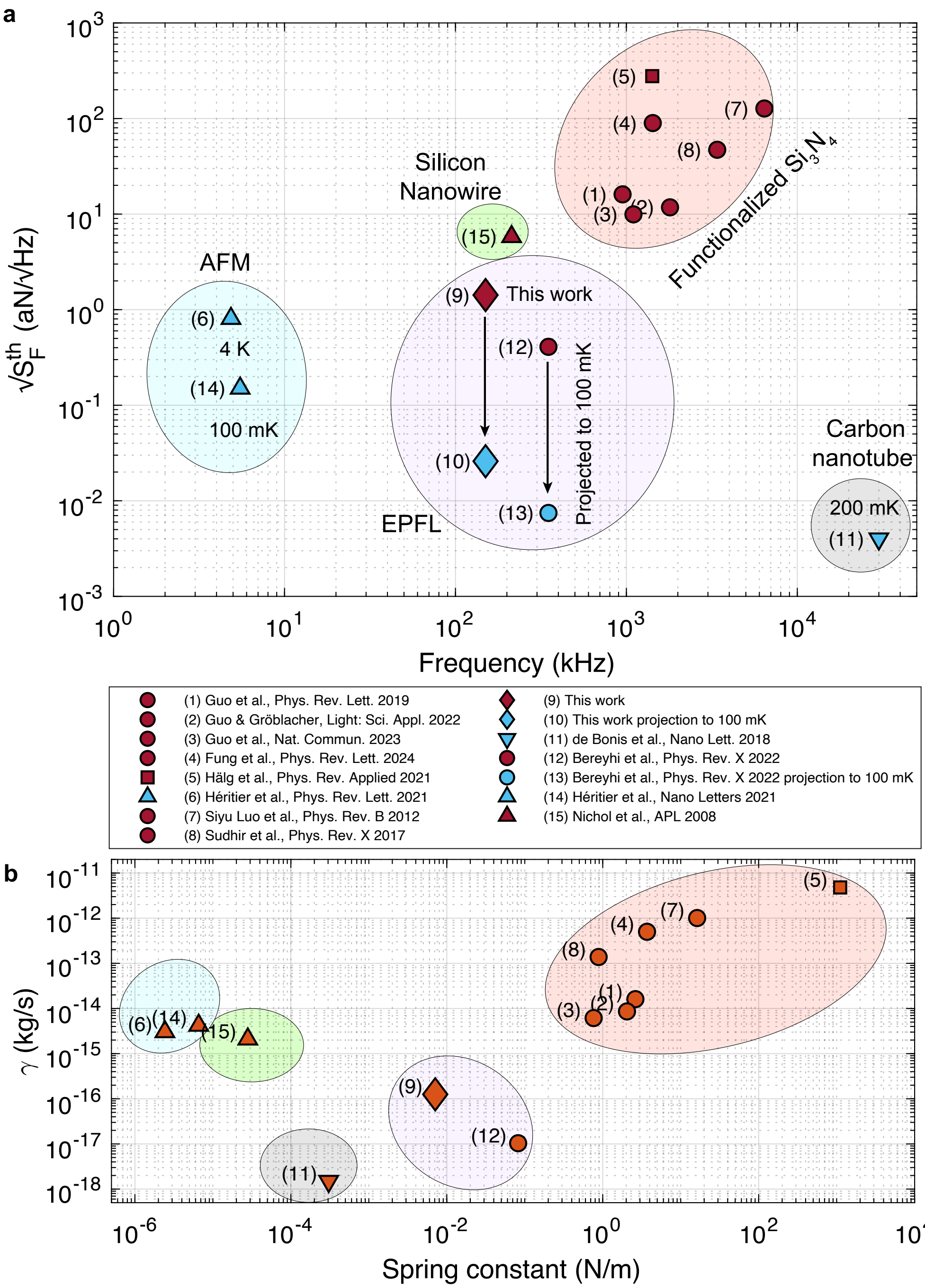

FIG. 3. **Overview of nanomechanical force sensors.** Red markers correspond to room temperature and blue circles to cryogenic temperature with the temperature written next to the markers.

observed no significant improvements.

The use of metallic coating is not considered for multiple reasons. First of all, it is not guaranteed that a metallic coating would necessarily mitigate NCF as in that case other effects such as non-uniformitty of the electric potential in the gap due to voltage patches

can give rise to dissipation [16]. We further note that some of the seminal observations of NCF [16, 17] employ metal coated cantilever tips interacting with metallic samples, and in the absence of any potential applied between the tip and the sample. Second of all, metallic coatings would significantly hinder the intrinsic performance of our devices. A metallic coating that effectively screens the electric field has to be smooth with a good coverage. Different deposition techniques on dielectrics (atomic layer deposition, sputtering or evaporation) require at least a few nanometers of deposition to provide such a good coverage [30–33]. This required metal thicknesses already represent a substantial added mass for the ultra-thin (20 nm) ultracoherent SiN nanomechanical resonators we present in Fig. 3 in the main text: a 5 nm Au layer increases the areal mass by $\sim 160\%$, corresponding to a $\sim 40\%$ reduction in resonance frequency. More critically, it is known that for high-Q silicon nitride resonators, the intrinsic loss (i.e. Q_{int}) is dominated by the surface properties [34]. Based on what has been measured for intrinsic quality factor of gold [35], introduction of a few nanometer thick layer would completely dominate the losses and limit the quality factor of our devices to $Q \sim 10^4 - 10^5$ and therefore prevent the observation of NCF.

For these reasons, I suggest that the authors submit the work to a more specialized journal such as APL or PRApp.

We hope that we have convinced the reviewer that our manuscript goes beyond a technical contribution and discusses physics that can appeal to a broad audience. We hope that we have also made a point that mitigation of NCF in these devices is not a straightforward task and requires dedicated studies.

Before publication anywhere, I would suggest that the authors also address the following points:

1) The authors clearly point out that dissipation, in their definition Q/ω, is the limiting factor in force measurements. My naive understanding of dissipation dilution is that one can increase Q by applying tension to a clamped mechanical oscillator. Stress is applied to increase Q, by increasing the stored mechanical energy, while leaving losses unchanged. This implies that while Q should increase proportionally with stress, so should the resonant frequency ω. This would then leave the dissipation and resulting force sensitivity unchanged. Is this incorrect? If so, why? Some intuitive explanation of these points would help clarify what can be achieved by dissipation dilution for readers outside the field.

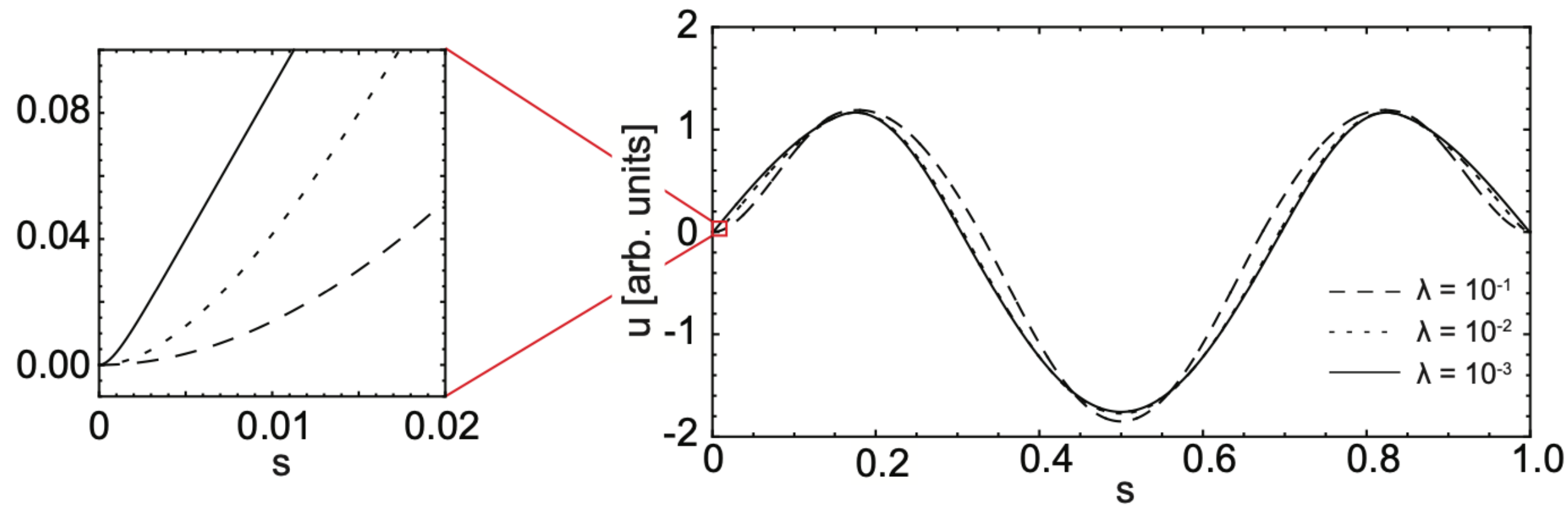

FIG. 4. **Modification of the mode shape by applying tension (adapted from Ref [36]).** Shapes of third order flexural modes of beams assuming the values of $\lambda = 10^{-1}, 10^{-2}, 10^{-3}$. The zoomed-in region shows the mode in the clamping regions, which illustrates an increase in mode curvature around clamps with the reduction of λ.

We are happy to elaborate more on this good question brought up by the reviewer. The statement that the introduction of the stress only modifies the resonance frequency of the modes and leaves the losses unchanged is not correct in general. For a given mechanical mode, the dissipated energy per oscillation depends on the mode shape and the mode shape can be dramatically modified when tension is applied [36]. For example, for a doubly clamped beam, considering a fixed length, as shown in Fig. 4, the mode curvature (which determines the bending loss) depends on $\lambda = \sqrt{\frac{E}{12\sigma}}\frac{h}{L}$, which depends on the stress σ. To demonstrate the impact of the modification of the mode shape on the damping rate we use FEM simulations to compute it for a doubly clamped beam. In Fig. 5 we show the frequency and damping rate of the fundamental mode of a uniform string with length 250 µm as a function of the built-in stress. When the stress is too low, the elasticity properties are determined by the Young's modulus of the material and hence neither the frequency nor the damping rate depend on the stress. As stress increases, as expected the resonance frequency increases. Similar to what is shown in Fig. 4 the bending distribution along the mode changes, concentrating the loss more on the clamping points. As shown in Fig. 5(d), the damping rate is initially *diluted* and then it saturates to a constant value. The saturation occurs when the applied tension dominates the elastic dynamics and the mode shape asymptotes to a sinusoidal form $\sin \pi x/L$. In this regime, the derivative of the mode shapes that determines the losses of the mode does not depend on the stress and further enhancement of the Q for higher stress

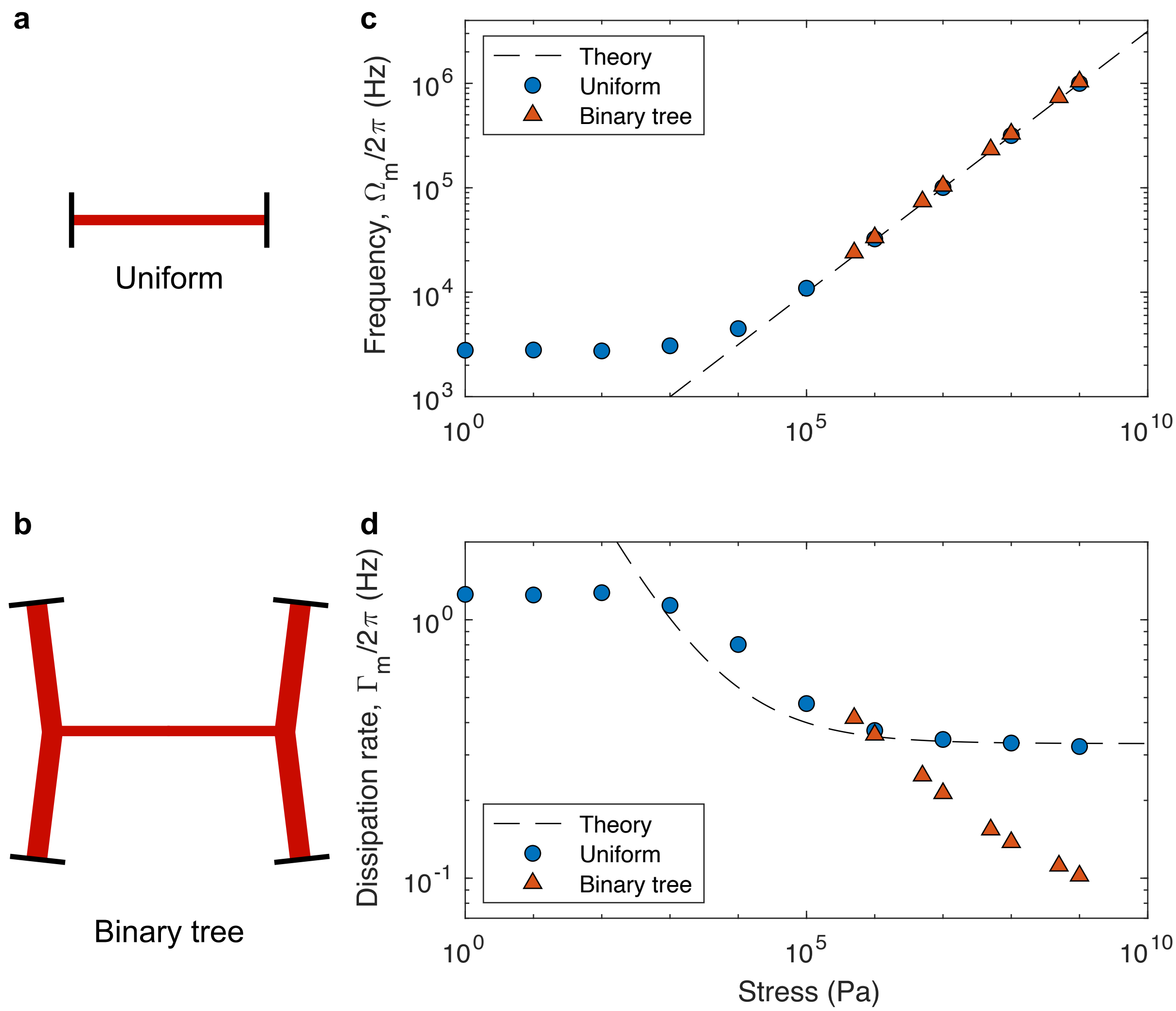

FIG. 5. **Dependence of dissipation on the stress.** **a** Schematic of a uniform doubly-clamped string. **b** Schematic of a binary-tree resonator with one branching generation. **c** Resonance frequency of the fundamental modes of the uniform (blue circles) and binary-tree (orange circles) strings as a function of the initial stress, obtained from FEM simulations. The dashed line corresponds to the dissipation-dilution theory at the high-stress limit. **d** Dissipation rate of the fundamental modes of the uniform (blue circles) and binary-tree (orange circles) strings as a function of the initial stress, obtained from FEM simulations. The dashed line corresponds to the dissipation-dilution theory at the high-stress limit.

is only due to the increase of the resonance frequency. This is the regime were the intuitive picture of the reviewer is valid. Nevertheless, for fixed resonance frequency, higher stress leads to higher quality factors. The dependence of the dissipation on the stress is more

dramatic for soft-clamped modes—modes with reduced derivative at the boundaries. For these modes, the contribution of the bending at the clamping points to the losses is reduced and the distributed bending along the mode becomes a dominant factor. This component has a higher order dependence on the stress due to the innate geometric nonlinearity of the strain [36], which results in further reduction of the dissipation for higher stress. For a simple demonstration of this effect, we simulate a binary-tree resonator shown in Fig. 5(b) whose geometry is designed in a way that for $\sigma = 1$ GPa, its fundamental mode (which is soft-clamped) has the same frequency as the fundamental mode of the 250 µm long uniform beam. As shown in Fig. 5(c,d), increasing the stress increases the frequency and at the same time reduces the dissipation rate. This is true even if this mode is only moderately soft-clamped due to the single branching generation.

Dissipation-dilution is generally a nonlinear effect and providing intuitive pictures is not always easy and especially not the main focus of this manuscript. The reviewer can refer to Ref. [37] where some toy models are introduced that are helpful for having a better intuitive picture of the phenomenon.

2) I don't understand (16) below line 420 and (17) below line 425. (16) appears to be different that relevant equation in reference 12, (21), which include yield strain. In (17) there appears to be a factor of $1/Q_{int}$ missing: should the intrinsic Q not go into this limit?

We thank the reviewer for their careful assessment. Q_{int} is indeed missing due to a typo.

Amendments to the manuscript

The missing Q_{int} in Eq. (17) in the methods section is added.

3) On line 20, the authors claim a "new regime of sensitivity" in the $\mathrm{aN}/\sqrt{\mathrm{Hz}}$ range. Although this is a new range for room-temperature mechanical resonators, it is certainly not a new range for mechanical force sensors at low temperature, with many such devices showing $\mathrm{aN}/\sqrt{\mathrm{Hz}}$ force sensitivity and some even in the 10s of $\mathrm{zN}/\sqrt{\mathrm{Hz}}$, e.g. suspended carbon nanotubes in the Bachtold group (ICFO) in 2013.

We thank the reviewer for bringing up this discussion as we believe it is about one of the most important aspects of our work. For all the state-of-the-art nanomechanical sensors, the force sensitivity is limited by the thermomechanical noise which its power spectral density

is given by

$$S_F^{\text{th}} = 4k_B T m \Gamma_m, \tag{2}$$

where m and Γ_m are the effective mass and the dissipation rate of the mechanical mode respectively and T is the temperature of the bath with which the mode is thermalized. Since S_F^{th} is directly proportional to the bath temperature, a fair comparison between different sensors, that truly reflects the device abilities, must be done at the same temperature. For a clear comparison, we have prepared a review of force sensitivities of different platforms both at room and cryogenic temperatures in Fig. 3. In this graph, the devices cited in the introduction are grouped as "Functionalized Si_3N_4", two state-of-the-art AFM cantilevers are grouped as "AFM", The "EPFL" group includes the present work and our previous work [26] which has the lowest room temperature S_F^{th}. We have also included a carbon nanotube sensor as a reference [38]. One can clearly see that the devices developed by us at room temperature are already on par with the cryogenic AFM cantilevers. For a fair comparison, we have included a projection of our devices if they are operated at 100 mK temperature. This projection only involves changing the temperature in Eq. 2 from 300 K to 100 mK and does not assume any enhancement of the quality factor (despite having been observed for Si_3N_4 resonators [39]). In case of the carbon nanotube (CNT), the atomic dimensions of them make them too fragile for any practical force sensing application. In spite of that, we also see that despite the nearly 10^6 times difference in the mass, the projection of our devices to 100 mK already reaches the level of 10s of $\text{zN}/\sqrt{\text{Hz}}$ and below. Cooling our devices to 10 mK, and in addition having at least factor of 3 improvement in the Q that has been observed, makes the force sensitivity of our devices to even surpass that of the carbon nanotube. These projections, show that ultracoherent nanomechanical resonators truly exhibit a new regime of sensitivity in micro-fabricated devices. We have included this graph in the Supplementary Information of the resubmitted manuscript.

Amendments to the manuscript

Figure 3 is added to the Supplementary Information as a new section.

III. REVIEWER 3

A. Summary of the key results

The authors measure the non-contact friction experienced by ultracoherent mechanical resonators near surfaces. The dependence of non-contract friction on frequency (i.e. oscillator mode) and distance is consistent with a loss mechanism in which static charges on the mechanical resonator interact with dielectric fluctuations in nearby surface. Three other plausible mechanisms are considered and ruled out. The measured friction data was fit to obtain charge densities and loss tangents, giving physically reasonable results.

B. Originality and significance

1. *This is a seminal paper and should be published immediately with minor revisions.*

2. *The ultracoherent mechanical resonators studied here are among the most most force-sensitive objects ever brought into close proximity to a surface. Thus the measured non-contact frictions are milestone measurements.*

3. *This paper is significant because there is tremendous interest is using ultracoherent mechanical resonators in sensitive force measurements, like magnetic resonance force microscopy and electric force microscopy. This paper shows that force noise due to charges on the resonator interacting with dielectric fluctuations in the samples is a potent source of force noise. This paper suggests that the force sensitivity could be improved by reducing the static charge on the mechanical resonator.*

We would like to thank the reviewer for their detailed and positive evaluation of our manuscript. We appreciate their comment regarding the significance and the impact of our work.

C. Data and methodology

1. *The derivations in the Supporting Information are nicely described, and are needed to understand the equations used in the manuscript. Enough information is given to reproduce the theory.*

2. *It would be helpful to the student if they authors could compare and contrast their theory with prior theory in S. M. Yazdanian et al. J. Chem. Phys. (2008) 128:224706, Ref. 28.*

We thank the reviewer for evaluating our theoretical work in the supplementary information and suggesting the comparison to the work of Yazdanian et al.

In the works of Yazdanian et al. and Kuehn et al., the authors take an approach based on the fluctuation-dissipation theorem, where generally the damping rate introduced by a fluctuating force $F_\alpha(t)$, on a particle with mass m is given by $\Gamma_\alpha = S_{F_\alpha}(\omega)/4k_BT$, where $S_{F_\alpha}(\omega)$ is the power spectral density of $F_\alpha(t)$. In the case of the cantilevers, the fluctuating force is given by $qE(t)$, q is the charge of the cantilever and $E(t)$ is the electric field originating from the dielectric fluctuations of the surface nearby. This gives rise to Eq. 2.10 of Yazdanian et al.. The PSD of $E(t)$ is then computed using the fluctuation-dissipation theorem again (to be more exact, using the linear response theory of Kubo) which relates the linear response between the position of the point charge and the electric field (Eq. 3.2 of Yazdanian et al.) through Eq. 3.3. This approach eventually gives rise to the relation that relates the damping coefficient to the imaginary part of the linear response (see equations 3.7 and 3.13). In a way, for computing the damping coefficient, the use of fluctuation-dissipation theorem is unnecessary since the damping coefficient is technically *defined* as the imaginary part of the linear response. In our work in section I.D of the supplementary information, we have rigorously derived a general relation for the damping coefficient, based on computing the force acting on the resonator which does not require the use of the fluctuation-dissipation theorem. Yazdanian et al. also find the linear response via the *ad hoc* introduction of a "fictitious electric dipole". In our work, we have also shown that this introduction does not have to be *ad hoc* and it naturally emerges from displaced electrostatics.

The approach of fluctuation-dissipation theorem is necessary when the PSD of the fluctuating field is given by other physics such as blackbody radiation or stray electric fields. When dealing with electric fluctuations of purely thermal origin, these fluctuations are found using the stochastic electrodynamics theory (as detailed in the second part of Yazdanian et al., Ref. [40] and reviewed by Volokitin and Persson in [10]).

Amendments to the manuscript

To highlight the comparison between our work and the work of Yazdanian et al., we have added the following paragraph to the Supplementary Information, section I:
The previous theories developed for computing the NCF coefficient for AFM cantilevers, are based on modeling the interaction of a moving point charge with a lossy semi-infinite dielectric [17, 18]. The approach taken in these works is based on using the fluctuation-dissipation theorem to find the damping coefficient via finding the linear response between the position of the point charge and electric field induced by the dielectric [18]. In our work, we generalize these theories to arbitrary geometries and charge distributions and computing the damping coefficient through direct calculation of the dissipated energy.

D. Appropriate use of statistics and treatment of uncertainties

Experimental uncertainties are handled appropriately.

We thank the reviewer for endorsing our uncertainty estimation techniques.

E. Conclusions

The conclusions are sound and supported by the data.

We are happy to see that the reviewer has found our conclusions clear.

F. Suggested improvements

1. *Page 2, introductory paragraph. The introductory paragraph focusses on Q. But in many applications, the figure of merit is the dissipation $\Gamma = k/(\omega Q)$, with k the spring constant, ω the resonance frequency, and Q the quality factor. In Fig. 1(c) is would be useful if the authors could report the associated spring constant k for each of the three designs. Then the reader would have enough information to compute Γ and S_F^{th}, the power spectral density of thermal force fluctuations.*

   We thank the reviewer for this suggestion. In this work and all our previous works for the resonators and their high-Q modes we have always reported resonance frequency, damping rate and quality factor as we believe these are the quantities that can be

compared with no ambiguity. In contrast, for the effective mass and the spring constant there is always an ambiguity in their definition as they are reducing a distributed resonator to a point-like oscillator. For a resonator with mass density ρ_m, for a mode with frequency Ω_m and mode shape $U(\vec{x})$ the effective mass is defined based on equating the average kinetic energy of the mode with a point-like mass oscillating with the same frequency:

$$\int d\vec{x}\frac{1}{4}\rho_m\Omega_m^2|U(\vec{x})|^2 = \frac{1}{4}m_{\text{eff}}\Omega_m^2|U(\vec{x}_0)|^2, \tag{3}$$

where $|U(\vec{x}_0)|$ is the reference point where the effective mass is defined with respect to it. The choice of this reference point gives rise to the ambiguity. For example, the reviewer can see that for a simple doubly-clamped string with a mode shape of the form $\sin\frac{\pi x}{L}$, depending on where the reference point is chosen in the 0 to L range, Eq. 3 gives different values. The spring constant has the same issue as it similarly reduces the elastic energy to a point-like mass spring oscillator. For the AFM cantilevers this ambiguity is resolved as the entire community are concerned with the motion of the tip of the cantilever oscillating in its fundamental mode. However, in the broader communities of nanomechanics and optomechanics, where the mechanical resonators and mode shapes can be more complex, the definition of the effective mass and the spring constant are not always easily translatable among different platforms. While, resonance frequency, damping rate and quality factor are quantities that are defined for any harmonic mode of a resonator of any nature (not only mechanical) with no ambiguity. Especially quality factor, that is essentially the number of coherent oscillations that a mode makes before its energy gets damped, is a universal quantity that allows for the comparison of the *quality* of different resonators.

For these reasons, in this work we have also tried to use the unambiguous quantities wherever it is possible. An interesting theoretical observation is that for the case where the entire volume of the resonator contributes to NCF (i.e. the strings above substrates), the NCF-limited quality factor turns out to be independent of the effective mass (please see first section of the methods section and the supplementary information) and can be directly compared to the experimentally measured quality factor. Wherever the effective mass is used, we have made it clear that is it defined with respect to the maxima of the modes. With this definition, we have prepared the

overview plot in Fig. 3, added to the supplementary information, that shows damping coefficient versus the spring constant. We have also added the effective mass of the three designs in Fig. 1(c) to the figure caption so that the interested readers can obtain the desired quantities.

Amendments to the manuscript

In caption of Fig. 1 of the main text, we have added the effective mass of the three designs in panel (c).

2. *Page 3, line 42. Using Γ [1/s] for the damping rate instead of the dissipation constant $\Gamma = k/(\omega Q)$ [N s/m] differs from the canonical notation used in friction studies; for example, in Stipe PRL (2001), Reference 27. To avoid confusion, the authors should consider adoption Stipe's notation. They should at least point out that their notation differs from prior notation.*

As mentioned above, we would like the manuscript to be accessible for a broad audience. We have used Γ as the damping **rate** which is the convention across many communities (for example: optomechanics [41], trapped ions [42], quantum measurements [43] etc). In our manuscript, in line 42 it has been made clear that Γ_m refers to the damping rate and in line 101 that γ_{NCF} refers to the damping constant (as used by Yazdanian et al. [18]).

3. *Page 4, line 71. The authors state "This phenomenon, also known as noncontact friction (NCF), has only been observed in atomic force microscopes (AFMs) cantilevers [27–29] at nanometer-scale tip-sample distances." The authors should cite the important paper by S. Kuehn et al., Phys. Rev. Lett. (2006) 96:156103, doi:10.1103/PhysRevLett.96.156103. In both Stipe 2001, Ref. 27, and Kuehn 2006, non-contact friction is apparent at distance greater than 200 nm. Similar results are seen in J. G. Longenecker et al., ACS Nano (2012) 11(6): 9637, doi:10.1021/nn3030628, where non-contact friction is apparent out to 300 nm tip-sample separation for a high-compliance cantilever over a copper microwire. So the authors should consider using more precise language than "nanometer-scale tip-sample distances".*

We thank the reviewer for this constructive comment. The original intention of this sentence was not to point to the absolute sub-nanometer distances and more to emphasize the fact that typical inter-body distances in AFM systems are shorter than the one of optomechanical systems. For better clarity we have made the following modification.

Amendments to the manuscript

The sentences from line 68 to 73 are modified from

"*By identifying that the added loss is inversely proportional to the mechanical mode's frequency, we realize that it is due to an electrical interaction between the resonator and the dielectrics in its proximity (i.e. the optical cavity or the substrate). This phenomenon, also known as noncontact friction (NCF), has only been observed in atomic force microscopes (AFMs) cantilevers [16, 18, 44] at nanometer-scale tip-sample distances.*"

to

"*By identifying that the added loss is inversely proportional to the mechanical mode's frequency, we realize that it is due to an electrical interaction between the resonator and the dielectrics in its proximity (i.e. the optical cavity or the substrate) mediated by static charges. This phenomenon, also known as noncontact friction (NCF), has only been observed in atomic force microscope (AFM) cantilevers [16–18, 44], where a functionalized tip is brought in close distance from a sample to measure surface properties.*"

4. *Pages 6 and 7, lines 105 through 115, regarding the Fig. 1(c) calculations. To convert between the predicted quality factor Q and the fundamental underlying calculated quantity γ_{NCF}, one needs to know the spring constant k and resonance frequency ω. Could the authors please report k and ω and report and plot the associated γ_{NCF}? It is important to report γ_{NCF} so that other (future) authors can compare their measured dissipation to the measurements and calculations reported here.*

We thank the reviewer for this suggestion. We have prepared a new figure that for the

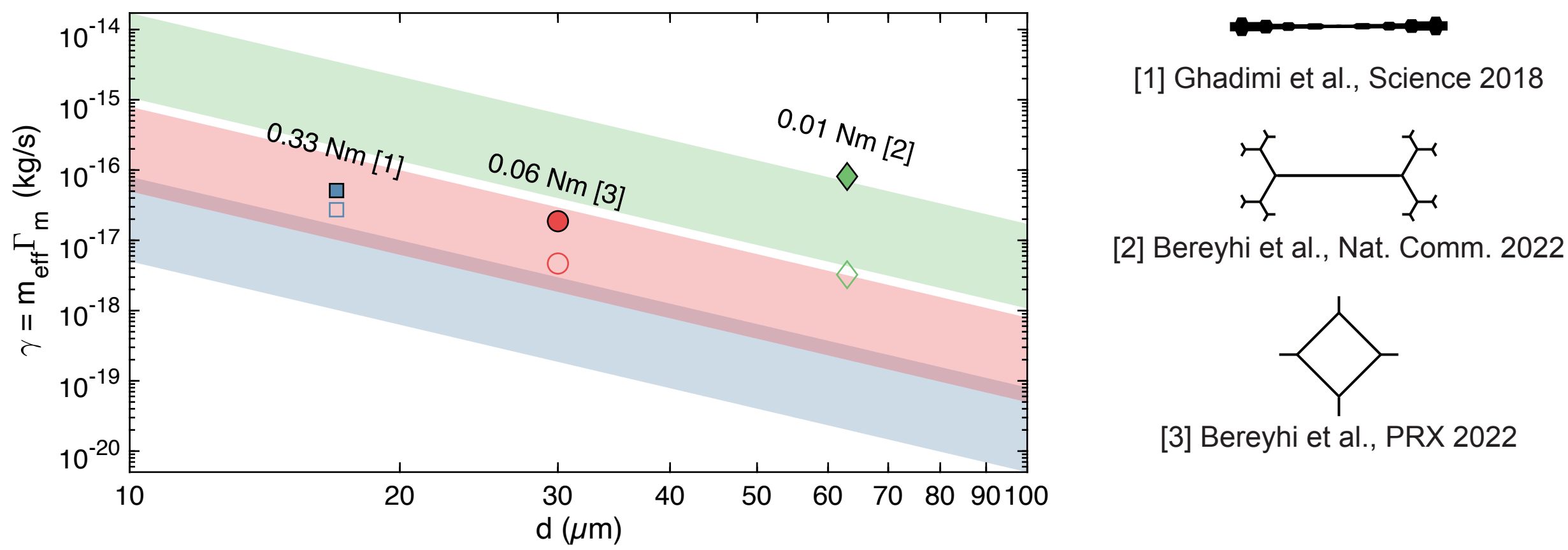

FIG. 6. **NCF in ultracoherent nanomechanical resonators above substrates** NCF damping induced by the substrate, limiting the quality factor of ultracoherent strings from Ref. [24–26]. The colors blue, red and green correspond to strained-engineered, hierarchical and polygon designs, shown in the right hand side. The ribbons correspond to the estimated NCF-induced damping coefficient for the spring constant (noted on the plot) and geometry of each design, suspended above a silicon substrate with a 4 nm native SiO_2 layer. The filled and empty markers correspond to the measured and simulated damping coefficient adapted from the references.

resonators shown in Fig. 1(c), we show that dissipation coefficient and spring constant. This figure is shown here in Fig. 6 and is added to the supplementary information.

Amendments to the manuscript

For the ease of comparison with previous and future works on friction measurements, we have added a different version of Fig. 1(c) of the manuscript to the Supplementary Information that shows dissipation coefficient and the spring constants of the ultracoherent resonators. This figure is here shown in Fig. 6.

5. *Page 7, line 133. Before "with E and" there is "h/L". I do not understad why the ratio h/L is present in the comma-separated list on line 133. Could the authors please explain?*

We thank the reviewer for noticing this typo as the comma should not be there. The expression is corrected in the resubmitted manuscript.

Amendments to the manuscript

In the main text in line 133 below Eq. 4, the expression for λ is reformatted to $\sqrt{\frac{E}{12\sigma}}\frac{h}{L}$.

6. *Page 8, line 145. The authors state "By using the literature values for SiO2 permittivity and loss tangent, we extract ...". Please give a citation and report the permittivity and loss tangent actually used in the calculation.*

The citation and the values are detailed in the Methods section in line 301.

Amendments to the manuscript

We have added a reference to the methods section in line 145.

7. *Page 8, lines 146, 147, and 166. In line 166, the areal charge density for Chip 1 is reported at 8.3e9 e/cm^2, which corresponds to 8.3e1 e/um^2. Given the width of 1.5 um and thickness of 0.227 um of the beam, the circumference is 3.45 um. I would thus expect a linear charge density for Chip 1 of 83 e/um^2 x 3.45 um = 286 e/um. This estimated linear charge density is in reasonable agreement with the 180 e/um reported on line 146. The authors should point out this agreement, and carry out a similar calculation for Chip 2.*

We thank the reviewer for noticing this numerical consistency. However, this conversion is not exactly correct. As explained in detail in section IV of the supplementary information, since the dimensions of the cross section of the string are comparable to the gap size, converting the surface charge densities to the linear one through multiplication by the circumference and then using Eq. (3) of the main text would not give the correct value for the NCF damping. Hence, one has to use the FEM simulation to obtain the correct values. However, the fact that the values we have obtained for the surface density using FEM modeling and what estimated by the reviewer are of the same order magnitude (as one would expect) shows the consistency of our FEM modeling methods.

8. *These are impressive measurements because the sample-induced change in γ being measured here is very small. As a point of comparison, to help the reader appreciate the*

significance of this work, the authors could compare the sample-related changes in γ detected here to prior measurements of non-contact friction by Stipe, Kuehn, and experiments cited in the Braakman review (see below).

We thank the reviewer this suggestion. We have added this comparison to our main text by modifying the last sentence of our conclusions section:

Amendments to the manuscript

We have modified our conclusions paragraph to highlight the fact that the level of NCF measured in this work is about 2-3 orders of magnitude lower than what has been previously measured.

G. References

1. *The way Refs. 16 and 17 are cited, the reader would think the references involve a magnetic resonance force microscope measurement. This is not the case. These are impressive references involve measurements of ultracoherent membranes near surfaces, but are not magnetic resonance force microscope measurements. The authors should cite additional papers in which force sensors near a surface are used to detected magnetic resonance.*

   We thank the reviewer for their careful reading. To clarify the citations, we have used the *scanning force microscopy* instead.

   Amendments to the manuscript

   In line 51, "*magnetic resonance force microscopy (MRFM)" is changed to* "scanning force microscopy*"*.

2. *The authors should cite the important paper by S. Kuehn et al., Phys. Rev. Lett. (2006) 96:156103, "Dielectric Fluctuations and the Origins of Noncontact Friction", doi:10.1103/PhysRevLett.96.156103. This is the first paper where, to this reviewer's*

knowledge, non-contact friction arising from dielectric fluctuations was first demonstrated experimentally and described theoretically.

We thank the reviewer for suggesting this seminal paper. A citation to this reference is added in line 73 in the introduction.

3. *Another important class of ultracoherent oscillator that the authors should cite are the silicon nanowires introduced by J. M. Nichol et al., Appl. Phys. Lett. (2008) 93: 193110, doi:10.1063/1.3025305, and reviewed by F. R. Braakman, Nanotechnology (2019) 30:332001, doi:10.1088/1361-6528/ab19cf. These nanowire oscillators have Γ values as low as 2e-15 kg/s. Can the authors compare the nanowire Γ to those of the oscillators reported here?*

We thank the reviewer for suggesting the references. We do not believe these oscillators belong to the ultracoherent family as what is meant by coherence typically refers to the coherence time (i.e. $\tau = Q/\Omega_m$). The binary-tree resonator of this work has $\tau \sim 500$ s and in previous works in the range of $\tau > 1$ hour [25, 26]. For the mentioned silicon nanowire this number is about $\tau = 48$ ms [45]. What brings the force sensitivity of these resonators down is their low mass which is in the 10^{-17} kg range, about 3 orders of magnitude lighter that Si_3N_4 strings.

We have prepared a new figure shown in Fig. 3, comparing the force sensitivity and dissipation for different nanomechanical resonators. We have included the suggested silicon nanowire reference to these plots as well.

G. Clarity and context

1. *Overall, this is a very well written paper.*

2. *In the abstract, replace "Here we show: it does." with "Here we show that it does."*

Since the sentence is grammatically correct we would like to keep the way it is currently written as it emphasizes the decisive conclusion of our work.

3. *Page 18, line 156. Replace "(see Methods for a discussion)." with "(See Methods ...)".*

4. *Page 21, line 387. Replace "for the modes with an anit-node" with "for the modes with an anti-node" (I think).*

5. *Page 22, line 406. Replace "vacuum friction. Van der Waals friction" with "vacuum friction, van der Waals friction".*

We would like to thank the reviewer for carefully reading the manuscript. All the typos mentioned are fixed.

Amendments to the manuscript

- In line 156, *see* is changed to *See.*
- In line 387 the typo is fixed: *anit-mode* to *anti-mode.*
- In line 406, the typo is fixed: *vacuum friction. Van der Waals friction* to *vacuum friction, van der Waals friction.*

---

[1] J. Wibbeler, G. Pfeifer, and M. Hietschold, Parasitic charging of dielectric surfaces in capacitive microelectromechanical systems (MEMS), Sensors and Actuators A: Physical **71**, 74 (1998).

[2] Y. Tsividis and C. McAndrew, *Operation and Modeling of the MOS Transistor* (Oxford University Press, 2011) google-Books-ID: m52JEQAAQBAJ.

[3] T. R. Albrecht, P. Grütter, D. Horne, and D. Rugar, Frequency modulation detection using high-Q cantilevers for enhanced force microscope sensitivity, Journal of Applied Physics **69**, 668 (1991).

[4] K. Y. Fong, W. H. P. Pernice, and H. X. Tang, Frequency and phase noise of ultrahigh Q silicon nitride nanomechanical resonators, Physical Review B **85**, 161410 (2012), publisher: American Physical Society.

[5] B. Kharbanda, A. Arabmoheghi, L. Catalini, M. Bereyhi, G. Benga, A. Zicoschi, C. L. Degen, T. J. Kippenberg, A. Eichler, and N. J. Engelsen, On-Chip Frequency Noise Cancellation in Nanomechanical Resonators using Cavity Optomechanics (2025), arXiv:2508.03301 [physics].

[6] A. N. Cleland and M. L. Roukes, Noise processes in nanomechanical resonators, Journal of Applied Physics **92**, 2758 (2002).

[7] W. Denk and D. W. Pohl, Local electrical dissipation imaged by scanning force microscopy, Applied Physics Letters **59**, 2171 (1991).

[8] P. Grütter, Y. Liu, P. LeBlanc, and U. Dürig, Magnetic dissipation force microscopy, Applied Physics Letters **71**, 279 (1997).

[9] B. Hoffmann, R. Houbertz, and U. Hartmann, Eddy current microscopy, Applied Physics A **66**, S409 (1998).

[10] A. I. Volokitin and B. N. J. Persson, Near-field radiative heat transfer and noncontact friction, Reviews of Modern Physics **79**, 1291 (2007).

[11] J. K. Srivastava, M. Prasad, and J. B. Wagner, Electrical Conductivity of Silicon Dioxide Thermally Grown on Silicon, Journal of The Electrochemical Society **132**, 955 (1985).

[12] M. J. Madou, *Fundamentals of Microfabrication: The Science of Miniaturization, Second Edition*, 2nd ed. (CRC Press, Boca Raton, 2018).

[13] W. A. Phillips, Two-level states in glasses, Reports on Progress in Physics **50**, 1657 (1987).

[14] S. Mittal, K. Adachi, N. Frattini, M. Urmey, S.-X. Lin, A. Emser, C. Metzger, L. Talamo, S. Dickson, D. Carlson, S. Papp, C. Regal, and K. Lehnert, Annealing reduces ${\mathrm{Si}}_{3}{\mathrm{N}}_{4}$ microwave-frequency dielectric loss in superconducting resonators, Physical Review Applied **21**, 054044 (2024).

[15] I. Dorofeyev, H. Fuchs, G. Wenning, and B. Gotsmann, Brownian Motion of Microscopic Solids under the Action of Fluctuating Electromagnetic Fields, Physical Review Letters **83**, 2402 (1999), publisher: American Physical Society.

[16] B. C. Stipe, H. J. Mamin, T. D. Stowe, T. W. Kenny, and D. Rugar, Noncontact Friction and Force Fluctuations between Closely Spaced Bodies, Physical Review Letters **87**, 096801 (2001).

[17] S. Kuehn, R. F. Loring, and J. A. Marohn, Dielectric Fluctuations and the Origins of Noncontact Friction, Physical Review Letters **96**, 156103 (2006), publisher: American Physical Society.

[18] S. M. Yazdanian, J. A. Marohn, and R. F. Loring, Dielectric fluctuations in force microscopy: Noncontact friction and frequency jitter, The Journal of Chemical Physics **128**, 224706 (2008).

[19] D. T. Krick, P. M. Lenahan, and J. Kanicki, Nature of the dominant deep trap in amorphous silicon nitride, Physical Review B **38**, 8226 (1988).

[20] G. Anetsberger, O. Arcizet, Q. P. Unterreithmeier, R. Rivière, A. Schliesser, E. M. Weig,

J. P. Kotthaus, and T. J. Kippenberg, Near-field cavity optomechanics with nanomechanical oscillators, Nature Physics **5**, 909 (2009).

[21] E. Gavartin, P. Verlot, and T. J. Kippenberg, A hybrid on-chip optomechanical transducer for ultrasensitive force measurements, Nature Nanotechnology **7**, 509 (2012).

[22] J. Guo, R. Norte, and S. Gröblacher, Feedback Cooling of a Room Temperature Mechanical Oscillator close to its Motional Ground State, Physical Review Letters **123**, 223602 (2019).

[23] J. Guo and S. Gröblacher, Integrated optical-readout of a high-Q mechanical out-of-plane mode, Light: Science & Applications **11**, 282 (2022).

[24] A. H. Ghadimi, S. A. Fedorov, N. J. Engelsen, M. J. Bereyhi, R. Schilling, D. J. Wilson, and T. J. Kippenberg, Elastic strain engineering for ultralow mechanical dissipation, Science **360**, 764 (2018).

[25] M. J. Bereyhi, A. Beccari, R. Groth, S. A. Fedorov, A. Arabmoheghi, T. J. Kippenberg, and N. J. Engelsen, Hierarchical tensile structures with ultralow mechanical dissipation, Nature Communications **13**, 3097 (2022).

[26] M. J. Bereyhi, A. Arabmoheghi, A. Beccari, S. A. Fedorov, G. Huang, T. J. Kippenberg, and N. J. Engelsen, Perimeter Modes of Nanomechanical Resonators Exhibit Quality Factors Exceeding ${10}^{9}$ at Room Temperature, Physical Review X **12**, 021036 (2022).

[27] D. Shin, A. Cupertino, M. H. J. de Jong, P. G. Steeneken, M. A. Bessa, and R. A. Norte, Spiderweb Nanomechanical Resonators via Bayesian Optimization: Inspired by Nature and Guided by Machine Learning, Advanced Materials **34**, 2106248 (2022).

[28] A. Cupertino, D. Shin, L. Guo, P. G. Steeneken, M. A. Bessa, and R. A. Norte, Centimeter-scale nanomechanical resonators with low dissipation, Nature Communications **15**, 4255 (2024).

[29] M. Kumph, C. Henkel, P. Rabl, M. Brownnutt, and R. Blatt, Electric-field noise above a thin dielectric layer on metal electrodes, New Journal of Physics **18**, 023020 (2016).

[30] S. Liang, M. Schwartzkopf, S. V. Roth, and P. Müller-Buschbaum, State of the art of ultra-thin gold layers: formation fundamentals and applications, Nanoscale Advances **4**, 2533 (2022), publisher: RSC.

[31] T. Andersson, The electrical properties of ultrathin gold films during and after their growth on glass, Journal of Physics D: Applied Physics **9**, 973 (1976).

[32] J. Hämäläinen, M. Ritala, and M. Leskelä, Atomic Layer Deposition of Noble Metals and Their Oxides, Chemistry of Materials **26**, 786 (2014), publisher: American Chemical Society.

[33] T. Aaltonen, M. Ritala, Y.-L. Tung, Y. Chi, K. Arstila, K. Meinander, and M. Leskelä, Atomic layer deposition of noble metals: Exploration of the low limit of the deposition temperature, Journal of Materials Research **19**, 3353 (2004).

[34] L. Villanueva and S. Schmid, Evidence of Surface Loss as Ubiquitous Limiting Damping Mechanism in SiN Micro- and Nanomechanical Resonators, Physical Review Letters **113**, 227201 (2014).

[35] M. J. Seitner, K. Gajo, and E. M. Weig, Damping of metallized bilayer nanomechanical resonators at room temperature, Applied Physics Letters **105**, 213101 (2014).

[36] S. A. Fedorov, N. J. Engelsen, A. H. Ghadimi, M. J. Bereyhi, R. Schilling, D. J. Wilson, and T. J. Kippenberg, Generalized dissipation dilution in strained mechanical resonators, Physical Review B **99**, 054107 (2019).

[37] S. Fedorov, *Mechanical resonators with high dissipation dilution in precision and quantum measurements*, Ph.D. thesis, EPFL (2021).

[38] S. L. de Bonis, C. Urgell, W. Yang, C. Samanta, A. Noury, J. Vergara-Cruz, Q. Dong, Y. Jin, and A. Bachtold, Ultrasensitive Displacement Noise Measurement of Carbon Nanotube Mechanical Resonators, Nano Letters **18**, 5324 (2018), publisher: American Chemical Society.

[39] M. Rossi, D. Mason, J. Chen, Y. Tsaturyan, and A. Schliesser, Measurement-based quantum control of mechanical motion, Nature **563**, 53 (2018).

[40] J. R. Zurita-Sánchez, J.-J. Greffet, and L. Novotny, Friction forces arising from fluctuating thermal fields, Physical Review A **69**, 022902 (2004).

[41] M. Aspelmeyer, T. J. Kippenberg, and F. Marquardt, Cavity optomechanics, Reviews of Modern Physics **86**, 1391 (2014).

[42] M. Brownnutt, M. Kumph, P. Rabl, and R. Blatt, Ion-trap measurements of electric-field noise near surfaces, Reviews of Modern Physics **87**, 1419 (2015).

[43] A. A. Clerk, M. H. Devoret, S. M. Girvin, F. Marquardt, and R. J. Schoelkopf, Introduction to quantum noise, measurement, and amplification, Reviews of Modern Physics **82**, 1155 (2010).

[44] M. Poggio and C. L. Degen, Force-detected nuclear magnetic resonance: recent advances and future challenges, Nanotechnology **21**, 342001 (2010).

[45] J. M. Nichol, E. R. Hemesath, L. J. Lauhon, and R. Budakian, Displacement detection of silicon nanowires by polarization-enhanced fiber-optic interferometry, Applied Physics Letters **93**, 193110 (2008).

Response to the reviewers' comments for:
Noncontact friction in ultracoherent nanomechanical resonators near dielectric materials

Amirali Arabmoheghi,[1,*] Alessio Zicoschi,[1] Guillermo Arregui,[1] Mohammad J. Bereyhi,[1] Yi Xia,[1] Nils J. Engelsen,[1,2] and Tobias J. Kippenberg[1,†]

[1]*Swiss Federal Institute of Technology Lausanne (EPFL), CH-1015 Lausanne, Switzerland*

[2]*Department of Microtechnology and Nanoscience (MC2), Chalmers University of Technology, SE-412 96 Göteborg, Sweden*

The points raised by the reviewers are addressed in the following. The original text by the reviewers is written in *italic blue* and our reply is written in black text. The amendments to the manuscript are also noted in red. Please note that the amendments might not be immediately visible in the published version of the manuscript as the final version of the manuscript is adapted to the format of Nature Physics.

I. REVIEWER 1

Dear Authors,

Thank you very much for your constructive reply to my earlier comments and questions. I agree with your answer and I am happy to see these answers also reflected in the manuscript. Therefore, I recommend to accept the manuscript for publication.

Best regards,

We thank the reviewer for the second positive evaluation of our work and for recommending the manuscript for publication. We are happy to see that the reviewer is satisfied with our responses and the modifications we have made to the manuscript.

II. REVIEWER 2

The revised manuscript "Noncontact friction in ultracoherent nanomechanical resonators near dielectric materials" is clearly written and the experiments and modelling appear careful and technically strong. The authors have responded conscientiously to the referees' technical requests, and the presentation has improved substantially. In that sense, I consider the work technically publishable.

My remaining concern is primarily one of framing and scope: the manuscript sometimes presents the central physical mechanism as unexpected or as a newly identified dissipation channel, whereas the underlying phenomenon (dielectric-loss–mediated noncontact friction from static charges) is well established in the AFM/force-microscopy literature. The value of the present work, in my view, is that it brings this known mechanism into the ultracoherent/dissipation-dilution nanomechanics context, quantifies its impact for state-of-the-art resonators and integrated nanophotonic geometries, and provides a modelling

* amirali.arabmoheghi@epfl.ch
† tobias.kippenberg@epfl.ch

framework and design guidance that will be useful for that community. That is an important contribution, but it is different from a claim of discovering an unexpected dissipation mechanism.

In their response to my point that the paper lacks the novelty required of a Nature Physics article, the authors write, "what makes our work interesting for readers is the cross-fertilization of ideas in the fields of atomic force microscopes and nano-/opto-mechanics: how a loss mechanism, new to one system (i.e. optomechanical systems), turns out to unexpectedly be due to a phenomenon known in a completely different system operating in a very different regime (i.e. atomic force microscopes)." While I certainly agree that pointing out the importance of disregarded but very relevant effects to a community of researchers is important, the fact that a small sub-community has disregarded them until now, hardly makes them new or unexpected. And calling this work an example of cross-fertilization between "completely different system[s]" is an exaggeration. To someone outside of condensed matter physics, as much of the readership of Nature Physics undoubtedly is, both optomechanical systems and AFMs are sub-micron-scale mechanical systems made of similar materials subject to physics in the same limits. My own suggestion to the authors is to simply make these points clear in the paper, rather than using language which gives the false impression that something new has been discovered. The authors have already taken most of this out, although 'unexpected loss mechanism' remains on line 64 of page 4.

In terms of significance, as the authors point out in their lengthy reply, the paper makes several important technical contributions including, measuring NCF with high precision, confirmation in observations on 'ultracoherent resonators' of the effects of NCF, design level insight into how to optimize force sensitivity in view of NCF., and perhaps most importantly detailed theoretical and numerical modelling of NCF. However, simulating and experimentally confirming the importance of dielectric loss in these mechanical resonators does not constitute a new physical discovery. As the authors themselves recount in their response, this was established long ago in AFM in systems that are essentially the same, except for minor technical differences (size, geometry, purpose). The difference in inter-body distances pointed out by the authors is specious because it is largely offset by a difference in resonator size and interaction area, which are also involved in these effects. In fact, two referees point out the same seminal papers for the authors to cite in their work (especially Yazdanian, Keuhn, and Stipe) on this loss mechanism, showing that the underlying physics

is well-established. It was understood and described in these papers in a very general sense and anyone with knowledge of this literature would expect the same mechanisms to play important roles as ‘ultracoherent resonators’ are brought near surfaces. In this sense, what is described is not new; it is, however, valuable to clearly point out to a community that for too long has ignored these points.

Ultimately, whether the work meets the particular breadth/novelty threshold for Nature Physics (as opposed to a more specialized venue) is an editorial judgement; my view is that its strongest contribution is the quantitative translation and design-relevant modelling of a known dissipation process into the ultracoherent nanomechanics setting, rather than the discovery of a new or surprising mechanism.

We thank the reviewer for the second evaluation of our work. Following their suggestion, we have removed the phrase "unexpected loss mechanism" from the manuscript.

III. REVIEWER 3

A. Summary of the key results

The revised manuscript is suitable for publication in Nature Physics.

The reviews were reasonable. The authors have responded thoroughly and thoughtfully to all reviewer comments.

B. Originality and significance

As the third reviewer, I agree with all but two of the criticisms of the first two reviewers.

I disagree with Reviewer 1 that temperature-dependent measurements are needed for a decisive test. The paper’s comparison between theory and experiment is already innovative and conclusive. The editor should appreciate that the authors are measuring non-contract friction in new regime of low loss, with a precision that is orders of magnitude better than all prior room-temperature measurements. Variable temperature measurements require a new apparatus and are expensive and painful; it is not reasonable to require them at this stage.

Reviewer 2 raised concerns about the significance of the work. The authors have done an excellent job addressing these concerns in both the reviewer-response document and the revised manuscript. The revised manuscript describes the work in a way that makes it clear the work is a significant advance. I therefore disagree with Reviewer 2’s assessment

that the manuscript is more appropriate for "a more specialized journal". Ultrasensitive force measurements are central to physics and this paper presents milestone results in force detection. The results deserve to be published in a premier journal.

The new sections on "Finite electrical conductivity" (manuscript) "D. Effect of finite conductivity" (supplementary information) are significant additions and strengthen the conclusions of the manuscript: the observed non-contact frictions is not due to conductive losses.

E. Conclusions

1. The new leading sentence of the conclusion is a big improvement that will help the general reader understand the significance of this work.

2. The new last two sentences are reasonable.

F. Suggested improvements

1. In Fig. 6 of the reviewer-response document, and Fig. 5 in the revised supplementary information, I believe that the spring constant labels in the plot should have units of N/m, not N m. I believe this mislabeling is a typographical error.

2. If the reviewer-response document is going to be published, there are a few typographical errors to fix. Throughout, replace (a) "Stippe" with "Stipe" and (b) "margine" with "margins" or "margin".

We thank the reviewer for their careful reading and for pointing out the typos. All the typos mentioned are fixed in the resubmitted manuscript and the reviewer-response document.
